# Calix[6]arene-Based [3]Rotaxanes as Prototypes for the Template Synthesis of Molecular Capsules

**DOI:** 10.3390/molecules28020595

**Published:** 2023-01-06

**Authors:** Federica Cester Bonati, Margherita Bazzoni, Caterina Baccini, Valeria Zanichelli, Guido Orlandini, Arturo Arduini, Gianpiero Cera, Andrea Secchi

**Affiliations:** Dipartimento di Scienze Chimiche, della Vita e della Sostenibilità Ambientale, Università di Parma, Parco Area delle Scienze 17/A, I-43124 Parma, Italy

**Keywords:** calix[6]arenes, [3]rotaxanes, NMR Spectroscopy, template synthesis, viologens

## Abstract

In this work, the ability of several bis-viologen axles to thread a series of heteroditopic tris(N-phenylureido)calix[6]arene wheels to give interwoven supramolecular complexes to the [3]pseudorotaxane type was studied. The unidirectionality of the threading process inside these nonsymmetric wheels allows the formation of highly preorganised [3]pseudorotaxane and [3]rotaxane species in which the macrocycles phenylureido moieties, functionalised with either ester, carboxylic, or hydroxymethyl groups, are facing each other. As verified by NMR and semiempirical computational studies, these latter compounds possess the correct spatial arrangement of their subcomponents, which could lead, in principle, upon proper bridging reaction, to the realisation of upper-to-upper molecular capsules that are based on calix[6]arene derivatives.

## 1. Introduction

One of the ultimate goals for contemporary chemists is to control the assembly and the functions of artificial nanosize structures, achieving the highest possible precision. The miniaturisation of the components and the availability of responsive devices are, in fact, critical issues for the development of modern nanotechnologies. The bottom-up approach provides the most promising strategy for controlling the aggregation at the nanometre level, which starts from nanoscale objects, namely atoms, ions, or molecules, to build up ordered nanostructures endowed with specific functions. Molecular capsules or cages are molecular scaffolds endowed with a nanosize cavity isolated from the bulk phase, which can host a complementary guest molecule [1,2,3,4,5,6,7]. These assemblies are of particular interest in the development of containers that can provide stability to highly reactive guests [8,9], such as phosphonium cations [10], cyclobutadiene [11], or even reactive elemental white phosphorus [12]. Capsular assemblies create a cavity in which the guest molecules frequently exhibit modifications as consequences of the confinement and, therefore, might be exploited as nanoscale “flasks” or reactors able to manipulate the physical and chemical properties of the trapped species [13,14]. A peculiar class of molecular capsules is one in which the hollow cavity is composed of two hemispherical or curved molecules. Rebek and colleagues synthesised several cavitand-based containers held together by a network of reversible interactions or covalent bridges. They explored the containers’ behaviour as hosts, supramolecular containers, and reactors [15,16]. Because of its preorganisation, calix[4]arene macrocycle has been an exploited building block for the synthesis of capsules in which the components are held together by noncovalent interactions [17], or they are covalently bound through the insertion of molecular bridges [18]. For the larger calix[6]arene macrocycle, the control of its large conformational flexibility remains a significant concern. Examples of noncovalent and covalent calix[6]arene-based capsules have been reported [19,20,21]. Our research group tackled the synthesis of molecular capsules via the hydrogen bond–guided self-assembly of tricarboxy [22,23] and triureido [24] calix[6]arene-based derivatives. We also reported on the formation and complexation properties of covalently linked double calix[6]arenes with imino and 1,4-phenylendiimino bridges [25], and the ability of a head-to-tail biscalix[6]arene to form a pseudorotaxane complex by threading a viologen salt was demonstrated [26]. However, synthesising covalently bound molecular capsules is often difficult because of the necessity of preorganising the capsule subcomponents in space before the final linking event. These challenging syntheses may thus benefit from a templating effect exerted by “ad hoc” complexed species capable of interacting with both molecular capsule subcomponents, as found, for example, in pseudorotaxanes and rotaxanes [27,28,29]. In these supramolecular complexes, the axial component and the macrocycles (wheels) are organised with the correct mutual geometrical arrangement because of the intermolecular recognition between the axle and the wheels.

**Figure 1:**
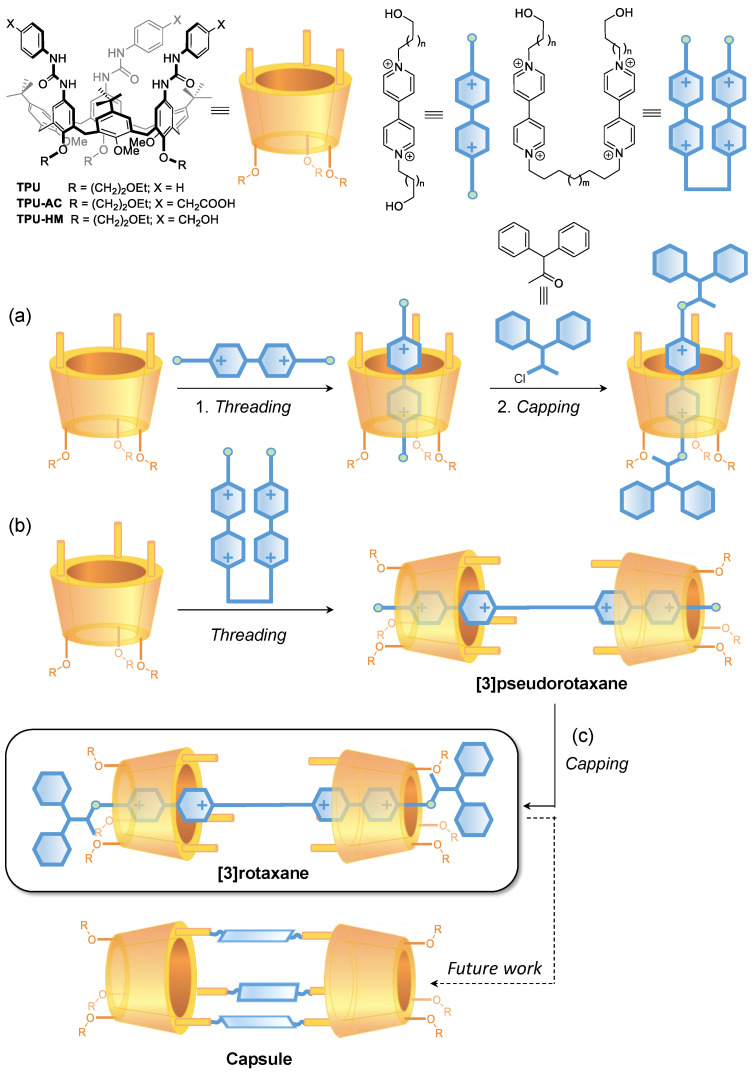
Schematic representation of calix[6]arene-based (**a**) [2]rotaxanes, (**b**) oriented upper-to-upper [3]pseudorotaxanes, and (**c**) [3]rotaxanes as prototypes for the synthesis of molecular capsules.

In the past two decades, we have exploited a series of tris(*N*-phenylureido) calix[6]arenes, such as **TPU** (see Figure 1), as hosts to synthesise molecular interlocked molecules (MIMs) [30]. It was discovered that electron-poor guests such as *N*,*N′*-dialkylviologen salts could thread the calix[6]arene annulus to give pseudorotaxanes complexes that, after thread capping (Figure 1a) or linking, would lead to the synthesis of [1]- and [2]rotaxanes and of [2]catenanes [31,32,33,34,35]. More enticing were the findings that in low polarity solvents, the threading process is governed by the functionality on the different rims of the calix[6]arene macrocycle. In particular, it occurs only from the macrocycle upper rim where the H-bond donating urea groups pivot the entrance of this ion pair into the aromatic cavity of the host [36]. Furthermore, because of the inherent asymmetry of the **TPU** cavity, introducing additional asymmetry elements in the thread, either as different stoppers or as lengths of the alkyl spacers, yields oriented [2]catenanes [33] and [2]rotaxanes [37]. On these premises, it is foreseen that the formation of the upper-to-upper bridged calix[6]arene capsules can benefit from the complexation of a bis-viologen axle capable of spatially orienting two suitably functionalised calix[6]arene wheels before their bridging. In the present study, we aim to evaluate the templating effect of a series of bis-viologen axles in promoting the formation of a series of upper-to-upper orientational [3]pseudorotaxanes (Figure 1b). In addition, the effect of the length of the alkyl chain separating two calix[6]arene wheels and the space between the stoppers and the macrocycles in the resulting [3]rotaxanes will also be tackled (Figure 1c). The method’s optimisation finally aims to synthesise new [3]rotaxanes in which the facing calix[6]arene upper rims are decorated with either carboxy or hydroxymethyl groups. The latter could potentially be employed as grafting points for the formation of covalent bridges between the two macrocycles to create upper-to-upper bridged capsules through a robust stepwise capping/clipping/stoppers removal/axle dethreading procedure.

## 2. Results

**Scheme 1:**
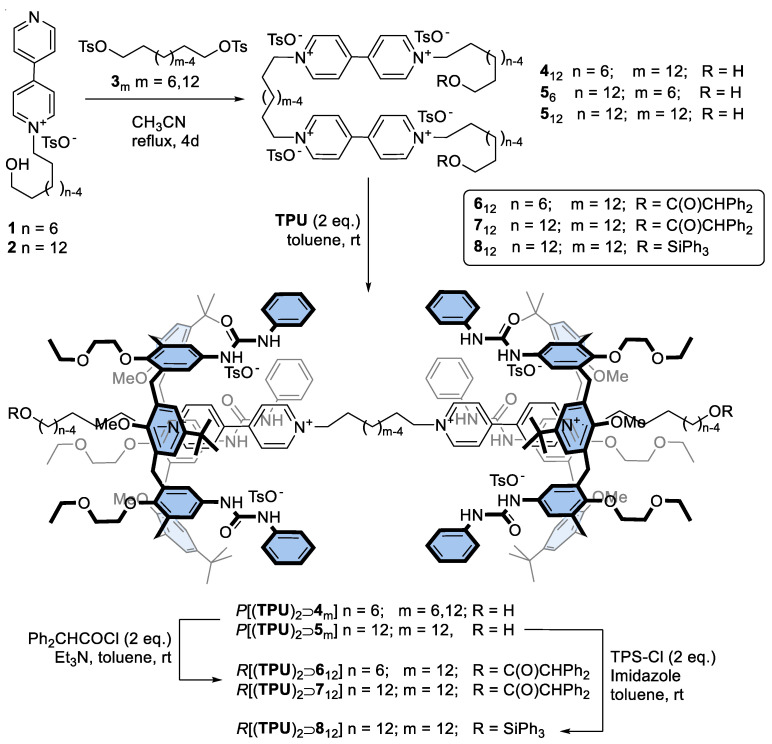
Synthesis of a series of calix[6]arene-based [3]rotaxanes using a threading/capping approach.

A series of axles of the types **4**_m_ (m = 12) and **5**_m_ (m = 6 and 12), where m indicates the length of the alkyl spacer between the viologen units, were prepared in good yields by reaction of the known pyridylpyridinium tosylates **1**-**2** with the corresponding ditosylates **3**_m_ in refluxing acetonitrile (see Scheme 1). Apart from the variable length of the internal spacer, these axles were also characterised by two external ω-hydroxyalkyl chains of different sizes: C6 for **4**_12_ and C12 for **5**_6,12_. A hydroxy group was present at the endings of these chains for the final capping reaction, eventually leading to the oriented [3]rotaxane formation. The variable length of the external chains was chosen to explore whether the formation of the [3]rotaxanes could be affected by steric hindrance between the axle stoppers and the macrocycle.

**Figure 2:**
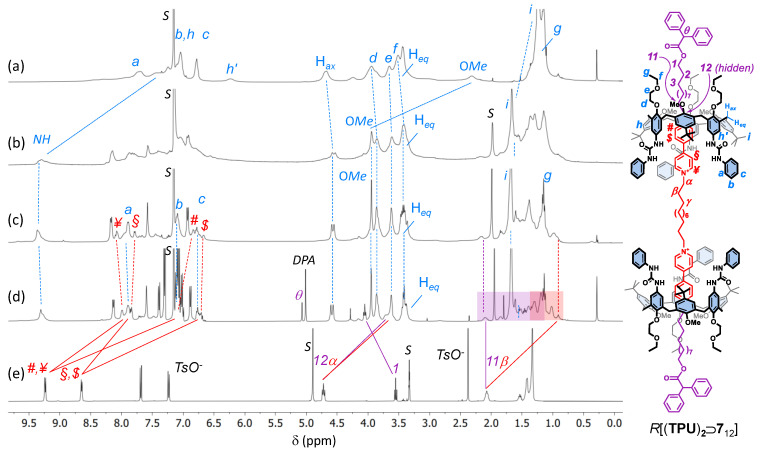
^1^H NMR stack plot (400 MHz, C_6_D_6_) of (**a**) **TPU**; (**b**) mixture of **5**_6_ with **TPU** (2 eq.); (**c**) [3]pseudorotaxane *P*[(**TPU**)**_2_**⊃**5**_12_]; (**d**) [3]rotaxane *R*[(**TPU**)**_2_**⊃**7**_12_] (the purple and orange boxes highlight the upfield-shifted resonances of the external and internal alkyl chains of the complexed dumbbell **7**_12_, while the resonance with the *DPA* label shows the methine proton of the diphenylacetate anions that have exchanged the tosylates upon axle stoppering); and (**e**) **5**_12_ (taken in CD_3_OD for solubility reasons). For the protons’ labels, see the sketch on the right (the tosylates have been omitted for more clarity).

The identity of the synthesised bis-viologen axles **4**_12_ and **5**_6,12_ was confirmed through a plethora of ^1^H NMR and ESI-MS measurements (see also Figures S1–S6 in Supporting Materials, SM). As an example, the ^1^H NMR spectrum of **5**_12_, taken in CD_3_OD (see Figure 2e), showed several diagnostic resonances, such as the multiplet centred at 4.70 ppm corresponding to the eight protons of the four methylene groups *α* and *12* (see the sketch in Figure 2 for the labelling) linked to the pyridinium moieties and a triplet at 3.53 ppm for the four protons of the two hydroxymethyl groups *1*. The two bipyridinium units resonate as two doublets, each integrating eight protons, at 9.22 (protons *¥*, *#*) and 8.63 ppm (*$*, *§*).

To evaluate the effect of the length of the axle components on the [3]pseudorotaxane formation, we used **TPU** as a wheel prototype (Scheme 1) [38]. Indeed, it is known that the threading of **TPU** with an asymmetric *N,N*′-dialkyl viologen axle in weakly polar solvents is unidirectional and occurs with the axle’s shortest alkyl chain through the macrocycle’s wider rim [39]. Therefore, the complexation reaction between **TPU** and any of the axles **4**_12_ and **5**_6,12_ would always lead to upper-to-upper [3]pseudorotaxane orientational isomers in which the two calix[6]arene upper rims are facing each other (see Scheme 1). To verify our hypotheses, we devised a simple NMR experiment: **5**_6_ (1 eq.) was suspended in a solution of **TPU** (2 eq.) in deuterated benzene. After stirring at room temperature for 24 h, the mixture appeared still heterogeneous and pale orangish. The solid suspension was filtered off, and the resulting homogeneous solution was submitted to NMR measurements. The corresponding ^1^H NMR spectrum (see Figure 2b) showed that the axle-wheel interaction restricted the fluxionality of **TPU** in C_6_D_6_ (see Figure 2a). A more significant proof of the wheels’ threading was given by the downfield shift, with sharpening, experienced by the signal of the methoxy groups of **TPU** (Δδ~1.4 ppm) and by the downfield shift of its NH signals (Δδ~2 ppm), which are engaged in H-bonding with the tosylates (cf Figure 2a,b, pale-blue continuous lines) [36].

More interesting results came from the threading experiments with the axles that had the longer internal C12 spacer **4**_12_ and **5**_12_. In both cases, the suspensions in benzene turned homogeneous and deep red after a few hours of stirring. This colour is usually a naked-eye sign of the pseudorotaxane formation because it derives from a charge transfer (CT) interaction between the π-rich cavity of **TPU** and the π-poor bipyridinium moiety of the axle. The formation of a [3]pseudorotaxane complex, labelled as *P*[(**TPU**)_2_⊃**5**_12_] in Scheme 1, was confirmed by the correct 1:2 ratio between the proton NMR signals assigned, on the basis of our previous studies on similar systems [38], to the threaded axle and the wheel (see Figure 2c and Figure S25, SM). Among these signals, it is worth noting an AX system (geminal coupling) of two doublets at 4.57 and 3.38 ppm, the latter partially overlapped by other intense resonances, for the bridging methylene protons of the macrocycle. Such a system confirms that the threaded wheels are adopting a *cone* conformation around the axle, as depicted in the sketch of Figure 2. The splitting of the resonances of the axle bis-pyridinium units instead witnessed the upper-to-upper orientation of the wheels. In the free **5**_12_, the two pairs of pyridinium protons labelled *¥*, # and *$*, *§* resonated as two undistinguishable doublets at 9.24 and 8.65 ppm, respectively (see Figure 2e). Upon inclusion, because of the asymmetry of the calix[6]arene cavity, they gave rise to four upfield-shifted broad resonances at 8.1, 7.8, 6.8, and 6.7 ppm, which were identified thanks to an HSQC experiment (see Figure S27, SM). The extent of each upfield shift is, however, different: the *ortho* (#) and *meta* (*$*) protons of the two pyridinium rings deeply engulfed in the cavities are more upfield shifted [−Δδ~2.1 (#) and 1.6 (*$*) ppm] than those of the “facing” pyridinium rings [−Δδ~1.2 (*¥*) and 0.75 (*§*) ppm]. A comparison of the resonances of the methylene groups directly linked to these pyridinium rings (*α* and *12*) showed that they underwent a minor upfield shift (−Δδ~1 ppm) and that their splitting was less significant (cf Figure 2d,e, red and purple lines). It is important to observe that the ^1^H NMR spectrum of the threading experiment having **5**_12_ as the axle showed narrower resonances than **5**_6_ (cf Figure 2b,c). This reduced fluxionality could thus be ascribed to a better geometrical fit of the two calix[6]arene wheels around the axle endowed with the longer C12 alkyl spacer. Taken together, these complexation experiments suggest that (*i*) the C6 internal spacer of **5**_6_ is too short for correct placement of the wheels around the two viologen units, and (*ii*) the threading of **4**_12_ and **5**_12_ selectively occurs, leading to upper-to-upper [3]pseudorotaxane orientational isomers, as depicted in Scheme 1 and Figure 2.

The synthesis of the interlocked species was then conducted by capping the protruding hydroxymethyl endings of the encapsulated axles **4**_12_ and **5**_12_ with bulky diphenylacetyl (DPA) stoppers in toluene, as previously reported (see Scheme 1) [37]. After chromatographic separation, [3]rotaxanes *R*[(**TPU**)_2_⊃**6**_12_] and *R*[(**TPU**)_2_⊃**7**_12_] were isolated in 15% and 21% of yield, respectively. As expected, the shorter C6 external alkyl spacer of **4**_12_ slightly reduced the success of the stoppering reaction for steric reasons (see Figures S29–S35, SM, for the characterisation of *R*[(**TPU**)_2_⊃**6**_12_]). The successful formation of the interlocked compounds was initially verified through HR-MS measurements. *R*[(**TPU**)_2_⊃**7**_12_] instead gave rise to a triply charged molecular ion (base peak at *m*/*z* = 1446.48779 with z = 3) (see Figure S41, SM). With respect to the ^1^H NMR spectra of their [3]pseudorotaxane precursors, those of *R*[(**TPU**)_2_⊃**7**_12_] (cf Figure 2c,d for *R*[(**TPU**)_2_⊃**7**_12_]) show (i) a general improvement of the signals’ resolution, (ii) a crowding of signals in the aromatic region, and (iii) the presence of a sharp signal at 5.02 ppm, labelled as *θ* in Figure 2d. In accordance with our previous studies on similar rotaxane systems [37,40], this signal was assigned to the methine proton of the stoppers introduced on the axle endings. The capping reaction also affects the resonance of the nearby methylene group (*1*). In the pseudorotaxane precursor, this group gives rise to an overlapped hidden triplet at 3.45 ppm, while in the [3]rotaxane, it is visible and downfield shifted (Δδ~0.5 ppm) at 4.05 ppm. Once again, the bis-pyridinium resonances of the capped thread (i.e., the dumbbell) in *R*[(**TPU**)_2_⊃**7**_12_] result largely upfield shifted compared with those of the free **5**_12_ (cf Figure 2d,e, red and purple solid lines). Thanks to a series of TOCSY experiments (see Figure S40, SM), we could also partially identify the resonances of the outer and internal alkyl spacers, starting from those of the methylene groups, labelled as *α* and *12* in Figure 2. According to the symmetric upper-to-upper arrangement of the calix[6]arene wheels of this interlocked structure, the resonances of the internal spacer, highlighted in the spectrum of Figure 2d with a shaded red box, experience a higher shielding effect than those of the external arms, highlighted with a shaded purple box. Similar NMR features were observed for *R*[(**TPU**)_2_⊃**6**_12_] (see Figures S29–S35, SM).

**Scheme 2:**
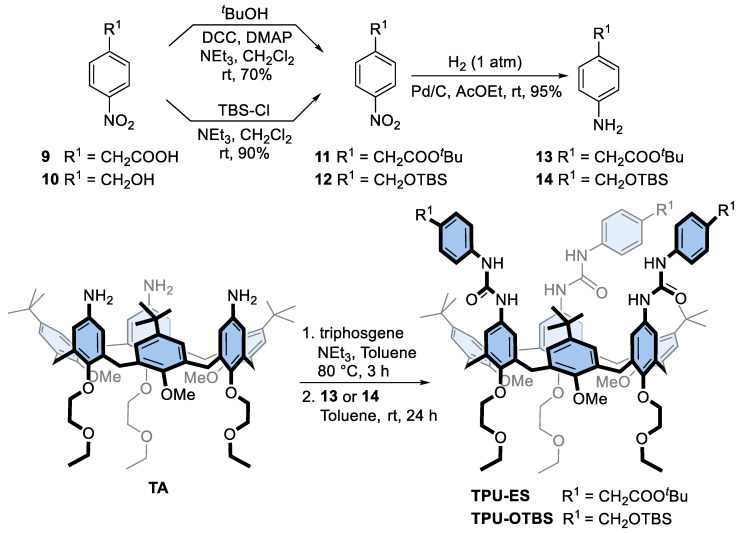
Synthesis of triphenylureido *p-*substituted calix[6]arene macrocycles **TPU-ES** and **TPU-OTBS**.

Prompted by the successful preparation of these interlocked structures*,* we planned to exploit the bis-viologen axles’ templating effect for the synthesis of upper-to-upper bridged calix[6]arene-based capsules, as schematised in Figure 1. First, however, it becomes mandatory to decorate the phenylurea units of **TPU** with functional groups that allow the linking of the macrocyclic subunits with bridges of proper length and rigidity. For this aim, we designed calix[6]arene wheels that present the carboxylic and hydroxymethyl groups onto their phenylureas, respectively protected by a *tert*-butyl ester (**TPU-ES**) and silyl ether (**TPU-OTBS**) (see Scheme 2). Indeed, these protecting groups can be removed by using conditions not affecting the ester functions of the DPA stoppers and the macrocycle urea moieties (vide infra). In particular, the ester groups can be reacted after deprotection and suitable activation with nucleophile bifunctional linkers, such as diols and diamines. In contrast, the hydroxymethyl ones can be reacted with activated dicarboxylic acids. Both wheels were prepared using the convergent synthesis depicted in Scheme 2.

First, the two amino derivatives **13** and **14**, bearing the carboxylic and hydroxymethyl-protected functionalities, were synthesised with an overall yield of 67% and 85%, respectively. Then, the known triamino calix[6]arene **TA** [24] was activated with triphosgene in toluene and reacted with either **13** or **14** to afford the target calix[6]arene derivatives **TPU-ES** and **TPU-OTBS** in 78% and 72% yields, respectively. Like **TPU**, the ^1^H NMR spectra of these calix[6]arene derivatives present several broad resonances. Nonetheless, those associated with the inserted functionalities are recognisable as two singlets at 3.40 and 1.44 ppm for **TPU-ES** (cf Figure 2a and Figure 4a) and as three singlets at 4.57, 0.91, and 0.05 ppm for **TPU-OTBS** (cf Figures S11 and S17, SM). ESI-MS measurements confirmed the identity of the novel macrocycles (see Figures S15 and S22, SM).

**Figure 3:**
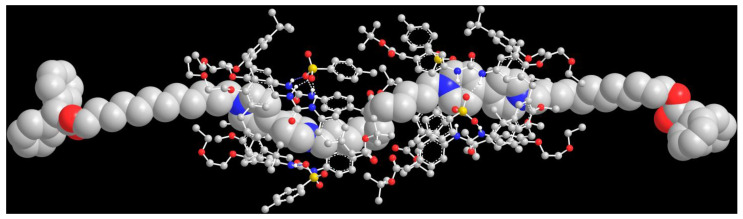
Perspective view of the minimised structure (PM7) of [3]rotaxane *R*[(**TPU-ES**)_2_⊃**7**_12_]. All hydrogen atoms, except those involved in H-bonding with the four tosylates, have been omitted for clarity (oxygen, red; sulphur, green; nitrogen, blue; the hydrogen bonds are highlighted with dashed lines). For clarity, dumbbell **7**_12_ has been depicted as a space-filling structure, while the two **TPU** macrocycles with a ball-and-stick representation.

To assess whether axle **5**_12_ could template the formation of the expected threaded species, we carried out some PM7 semiempirical calculations on a hypothetical [3]rotaxane built with two facing **TPU-ES** units and a DPA-stoppered axle (**7**_12_, see Scheme 1 for its structure) using the MOPAC 2016 software [40]. A view of the resulting minimised structure of *R*[(**TPU-ES**)_2_⊃**7**_12_] is depicted in Figure 3. It shows that the inner C12 alkyl unit of dumbbell **7**_12_ easily accommodates two facing units of **TPU-ES** without steric strain between the respective *p-*substituted phenylureas.

**Scheme 3:**
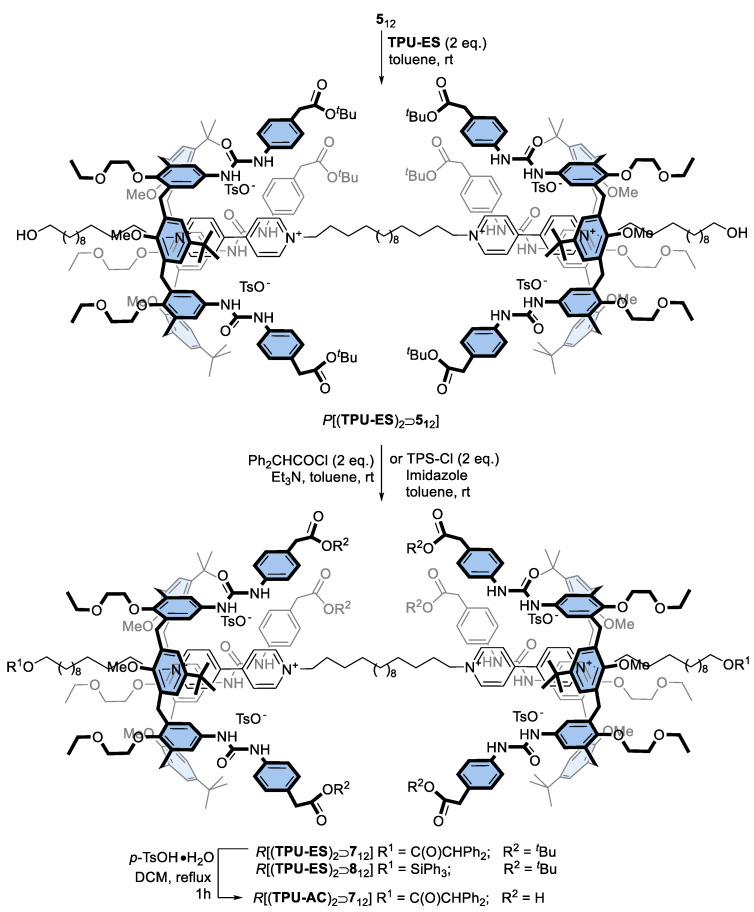
Synthesis of calix[6]arene-based [3]rotaxanes with *p-*functionalised phenylureas.

**Figure 4:**
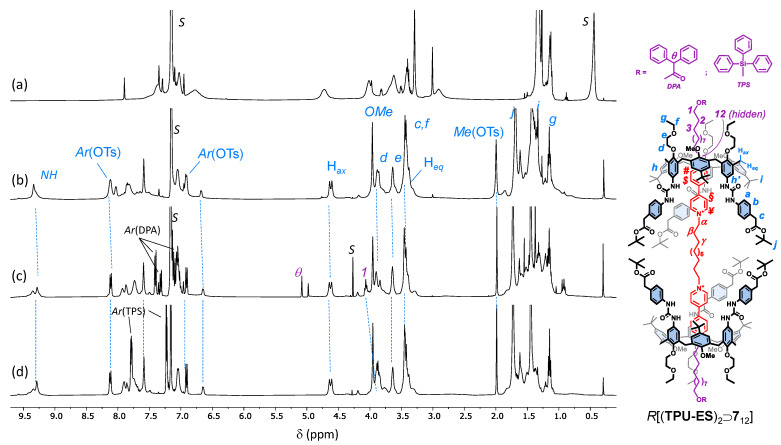
^1^H NMR spectra (400 MHz) of (**a**) **TPU-ES**, (**b**) [3]pseudorotaxane *P*[(**TPU-ES**)_2_⊃**5**_12_], (**c**) *R*[(**TPU-ES**)_2_⊃**7**_12_], and (**d**) [3]rotaxane *R*[(**TPU-ES**)_2_⊃**8**_12_] in C_6_D_6_ (see the sketch on the right for protons labels; the tosylates have been omitted for more clarity).

The synthesis of the [3]rotaxanes was thus performed as usual by suspending axle **5**_12_ in a toluene solution containing a twofold amount of the corresponding wheel (**TPU-ES** or **TPU-OTBS**; see Scheme 3). The resulting mixtures were stirred at room temperature for 24 h. However, the mixture containing **TPU-OTBS** remained heterogeneous, even after stirring with heat (80 °C) for 24 h. This unexpected result suggests that the bulkiness of the TBS-protecting group prevents the threading of the bis-viologen axle inside the cavity of this calix[6]arene derivative. Fortunately for us, the mixture with **TPU-ES** turned homogeneous and red. A portion of this solution was thus evaporated to dryness, taken up with benzene-d_6_, and submitted to a series of 1D and 2D NMR measurements to verify the formation of the [3]pseudorotaxane *P*[(**TPU-ES**)_2_⊃**5**_12_] with the desired upper-to-upper orientation of the calix[6]arene wheels. Its ^1^H NMR spectrum (Figure 4b) showed a signal pattern similar to *P*[(**TPU**)_2_⊃**5_12_**] (Figure 2c), except for the expected presence of the resonances relative to the methylene (*c*) and tert-butyl (*j*) groups at 3.45 and 1.70 ppm, respectively.

After the successful formation of the oriented [3]pseudorotaxane, the ω-hydroxymethyl endings of **5**_12_ were stoppered as usual with two equivalents of DPA-Cl to yield the novel [3]rotaxane *R*[(**TPU-ES**)_2_⊃**7**_12_] in 24% of yield, after chromatographic separation (see Scheme 3). MS-ESI and NMR measurements confirmed the success of these stoppering reactions. The HR-MS spectrum of the isolated compound showed a quadruple charged molecular ion (base peak at *m*/*z* = 1213.96853 with z = 4), whose isotopic pattern agreed with that of the target interlocked structure that had lost four tosylates (see Figure S60, SM). The ^1^H NMR spectrum (Figure 4c) showed a unique singlet at 5.08 ppm for the methine proton *θ* of the DPA stoppers. This signal accounted for the symmetric upper-to-upper arrangement of the two **TPU-ES** wheels around the dumbbell **7**_12_.

Prompted by these results, we eventually explored the possibility of employing complementary axle-stoppering units. For this aim, we opted for a triphenylsilyl (TPS) moiety because the bulkiness of this protecting group also allows its employment as a stopper for rotaxane synthesis. This strategy was quite successful, and the corresponding [3]rotaxane *R*[(**TPU-ES**)_2_⊃**8**_12_] was isolated in 17% of yield after chromatographic separation. As usual, both interlocked structures were characterised through MS and NMR analyses. The HR-MS spectrum (see Figure S67, SM) of *R*[(**TPU-ES**)_2_⊃**8**_12_] showed a quadruple charged molecular ion with *m*/*z* = 1245.97588 (z = 4). Unlike DPA, the TPS stoppers did not yield any diagnostic proton NMR resonance that can be exploited to assess which orientational isomer formed upon axle stoppering. However, the spectra of the two interlocked structures almost perfectly overlapped (cf Figure 4c,d), except in the presence of the spectrum of *R*[(**TPU-ES**)_2_⊃**8**_12_] of supplementary aromatic resonances, in the 7.8–7.2 ppm range, and the shift at higher fields (3.92 ppm, overlapped) of the previously visible resonance assigned to axle methylene group (*1*) (Figure 4c). An upfield shift, the latter, originated by the replacement of an electron-withdrawing group (DPA) with a donating one (TPS) onto the thread hydroxyl endings. Most importantly, like *R*[(**TPU-ES**)_2_⊃**7**_12_], the spectrum of *R*[(**TPU-ES**)_2_⊃**8**_12_] also showed a single sharp signal at 3.95 ppm for the three methoxy groups at the macrocycle lower rim. This would indicate that the two calix[6]arene macrocycles are experiencing an identical magnetic environment. In other words, they are adopting the expected upper-to-upper symmetric arrangement of the macrocycles around the dumbbell.

Finally, the deprotection of the carboxyl groups of *R*[(**TPU-ES**)_2_⊃**7**_12_] was accomplished, among the several attempts, by using *p*-toluenesulfonic acid monohydrate (TsOH∙H_2_O) in refluxing dichloromethane (see Scheme 3). The target [3]rotaxane *R*[(**TPU-AC**)_2_⊃**7**_12_] was isolated in good yield (60%) after chromatographic separation.

**Figure 5:**
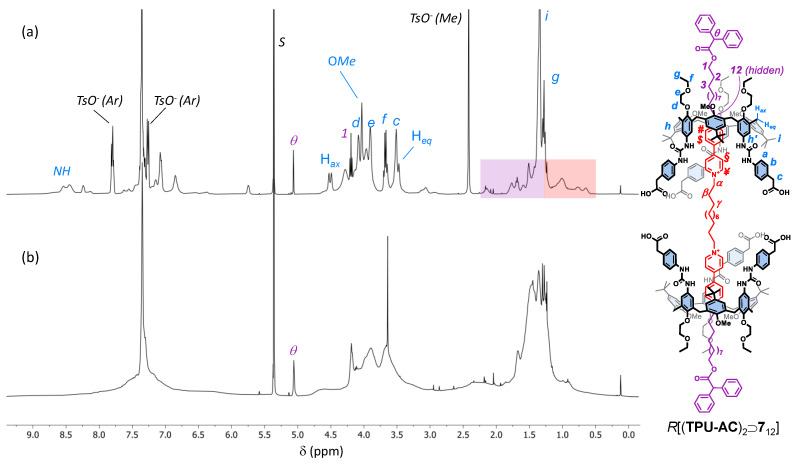
^1^H NMR spectra (400 MHz) of *R*[(**TPU-AC**)_2_⊃**7_12_**] in CD_2_Cl_2_ before (**a**) and after (**b**) the treatment of the solution with 4 eq. of TsOH∙H_2_O. The purple and orange boxes in (**a**) highlight the upfield-shifted resonances of the external and internal alkyl chains of the complexed dumbbell (**7**_12_), respectively (see the sketch on the right for protons labels; the tosylates have been omitted for more clarity).

Although HR-MS measurements confirmed the identity of *R*[(**TPU-AC**)_2_⊃**7_12_**] (see Figure S75, SM), the interpretation of its ^1^H NMR spectrum (see Figure 5b), taken in deuterated dichloromethane for solubility reasons, was anything but straightforward. Broad resonances characterised the entire spectrum except for the diagnostic methine signal (*θ*) of the DPA stoppers. The signals’ broadness could be explained by considering a marked fluxionality in this structure on the NMR timescale. Still, the reasons for such mobility were less clear when the structures of *R*[(**TPU-ES**)_2_⊃**7_12_**] and *R*[(**TPU-AC**)_2_⊃**7_12_**] were compared. However, a perusal of the spectrum revealed the total absence of the three tosylate counterion resonances, which are usually sharp and easily identified, thus suggesting that a neutral (zwitterionic) form of this [3]rotaxane, in which four out of six carboxyl groups were ionised, was present in the dichloromethane solution. As a result, a reasonable hypothesis for such mobility was that the lack of anion coordination could significantly reduce the preorganisation of the macrocycles. To verify our hypothesis, four equivalents of TsOH∙H_2_O were added to the dichloromethane solution of *R*[(**TPU-AC**)_2_⊃**7_12_**]. The new ^1^H NMR spectrum obtained (see Figure 5a) showed that the interlocked structure fully recovered its preorganisation, giving rise to sharper resonances assigned to the structure, thanks to a series of 2D NMR measurements (see Figures S72–S74, SM). Importantly, these findings confirmed the critical role played by the H-bonding donor ability of the phenylurea groups in the preorganisation and complexation abilities of this type of calix[6]arene macrocycle.

In summary, in this study, we reported the preparation, using a threading and capping approach, of a series of calix[6]arene-based upper-to-upper oriented [3]rotaxane isomers bearing diphenylacetyl or triphenylsilyl stoppers. The success in synthesising these rather-complicated interlocked structures was possible thanks to the template effect exerted by a bis-viologen thread functionalised at its ending with hydroxymethyl groups. Furthermore, the correct distance between the bis-pyridinium units of the axle in promoting the [3]rotaxane formation was explored by using axles having internal and external alkyl chains of different lengths. Finally, the isomeric selectivity leading to the desired upper-to-upper orientation of these rather complex interlocked structures was obtained thanks to the axle unidirectional threading process that in low polarity solvents always occurs through the largest access of the calix[6]arene cavity, i.e., that bearing the phenylurea groups.

The high functional group tolerance of the templating approach used for the [3]rotaxane synthesis has allowed the employment of calix[6]arene macrocycles decorated onto their phenylureas with different functional groups, such as the carboxyl, ester, and hydroxyl groups. Such functionalities were designed to be used as grafting points for inserting bridging units, eventually leading to the synthesis of calix[6]arene-based molecular capsules.

## 3. Materials and Methods

### 3.1. General

All solvents were dried using standard procedures; all other reagents were of reagent-grade quality obtained from commercial suppliers and used without further purification. Melting points were uncorrected. NMR spectra were recorded at 400 MHz for ^1^H and 100 MHz for ^13^C. Chemical shifts were expressed in ppm (δ) using the residual solvent signal as an internal reference (7.16 ppm for C_6_H_6_, 7.26 ppm for CHCl_3_, and 3.31 ppm for CD_2_HOD). The terms m, s, d, t, and q represent multiplet, singlet, doublet, triplet, and quadruplet, respectively; the term “br. s” means a broad signal. Other abbreviations used in the text are DPA = diphenylacetyl, TPS = triphenylsilyl, and TsO = tosylate. Mass spectra were recorded in the ESI mode. Compounds **1** [41], **TPU** [36], **TA** [24], **3**_6_ [42], **3**_12_ [43], **6**_12_ and **7**_12_ [38], **12** [44], and **14** [44] were synthesised according to published procedures.

### 3.2. Chemistry

#### 3.2.1. General Procedure for the Synthesis of the Bis-Viologen Axles **4_12_** and **5_6–12_**

In a sealed 100 mL glass autoclave, a solution of the appropriate pyridyl pyridinium tosylate (**1**–**2**, 0.6 mmol) and ditosylate (**3_m_**, 0.3 mmol) in dry acetonitrile (40 mL) was refluxed under vigorous stirring for 7 days. Afterwards, the solution was evaporated to dryness under reduced pressure.

**4**_12_: the solid residue of the evaporation was triturated with CH_3_CN to afford 0.3 g of product **4**_12_ as a solid white powder (63%). M.p. = 156–158 °C. ^1^H NMR (CD_3_OD, 400 MHz) δ = 9.22 (d, 8H, *J* = 6.9 Hz, H_#_ and H_¥_), 8.63 (d, 8H, *J* = 6.4 Hz, H_$_ and H_§_), 7.67 (d, 8H, *J* = 8.4 Hz, TsO), 7.22 (d, 8H, *J* = 8.0 Hz, TsO), 4.7–4.6 (m, 8H, H_α_ and H_6_), 3.55 (t, 4H, *J* = 5.6 Hz, H_1_), 2.36 (s, 12H, C*H_3_*, TsO), 2.1–2.0 (m, 8H, H_β_ and H_5_), and 1.5–1.3 (m, 28H, H_2–4_ and H_γ-ζ_) ppm. ^13^C NMR (CD_3_OD, 100 MHz): δ = 151.2, 147.0, 143.7, 141.7, 129.9, 128.3, 126.9, 63.3, 63.2, 62.6, 33.2, 32.5 (2 res.), 30.6, 30.5, 30.2, 27.2, 26.9, 26.4, and 21.3 ppm. ESI-MS(+): = *m*/*z* 1195.9 [M-TsO]^+^. For a complete proton assignment, see also Figure S1, SM.

**5**_6_: the solid residue of the evaporation was triturated with CH_3_CN to afford 0.3 g of **5_6_** as a solid white powder (65%). M.p. = 155–157 °C. ^1^H NMR (CD_3_OD, 400 MHz): δ = 9.25 (d, 4H, *J* = 6.9 Hz, H_¥_), 9.22 (d, 4H, *J* = 6.9 Hz, H_#_), 8.59 (d, 8H, *J* = 6.4 Hz, H_$_ and H_§_), 7.66 (d, 8H, *J* = 8.0 Hz, TsO), 7.21 (d, 8H, *J* = 8.0 Hz, TsO), 4.76 (t, 4H, *J* = 7.0 Hz, H_α_), 4.71 (t, 4H, *J* = 8.0 Hz, H_12_), 3.53 (t, 4H, *J* = 6.8 Hz, H_1_), 2.34 (s, 12H, TsO), 2.1–2.0 (m, 8H, H_β_, H_11_), and 1.6–1.3 (m, 42H, H_2–10_, H_γ-δ_) ppm. ^13^C NMR (CD_3_OD, 100 MHz): δ = 151.1, 151.0, 147.1, 146.9, 143.7, 141.7, 129.9, 128.2 (2 res.), 126.9, 63.2, 62.9 (2 res.), 33.6, 32.5, 31.9, 30.7, 30.6 (3 res.), 30.5, 30.1, 27.2, 26.9, 26.2, and 21.3 ppm. ESI-MS(+): *m*/*z* = 1279.1 [M-TsO]^+^. For a complete proton assignment, see also Figure S3, SM.

**5**_12_: the solid residue of the evaporation was recrystallised from CH_3_OH/CH_3_CN to afford 0.3 g of **5_12_** as a solid white powder (62%). M.p. = 196–198 °C. ^1^H NMR (CD_3_OD, 400 MHz): δ = 9.22 (d, 8H, *J* = 6.8 Hz, H_#_ and H_¥_), 8.63 (d, 8H, *J* = 6.4 Hz, H_$_ and H_§_), 7.67 (d, 8H, *J* = 8.0 Hz, TsO), 7.22 (d, 8H, *J* = 8.0 Hz, TsO), 4.70 (t, 8H, *J* = 7.6 Hz, H_α_ and H_12_), 3.53 (t, 4H, *J* = 7.0 Hz, H_1_), 2.36 (s, 12H, TsO), 2.1 (br. s, 8H, H_β_ and H_11_), and 1.5–1.3 (m, 52H, H_2–10_, H_γ-ζ_) ppm. ^13^C NMR (CD_3_OD, 100 MHz): δ = 151.2, 147.0, 143.6, 141.7, 129.9, 128.3, 126.9, 63.3, 62.9, 33.6, 32.5, 30.7 (2 res.), 30.6 (2 res.), 30.5, 30.2, 30.1, 27.2 (2 res.), 26.9, and 21.3 ppm. ESI-MS(+): *m*/*z* = 341.6 [M-3TsO]^3+^. For a complete proton assignment, see also Figure S5, SM.

#### 3.2.2. Synthesis of Tert-Butyl 2-(4-nitrophenyl)acetate (**11**)

First, 2-(4-nitrophenyl)acetic acid (2.5 g, 13.8 mmol) was dissolved in anhydrous dichloromethane (25 mL), then tert-butanol (3.1 g, 41.8 mmol) and DMAP (1.4 g, 11.0 mmol) were added. The mixture was cooled down to 0 °C, and DCC (4.1 g, 19.8 mmol) was slowly added. The solution was stirred at room temperature for 3 h, after which the formation of a white precipitate (DCU) was observed. The solid was filtered off through a Buchner filtration, and the organic phase was washed with water (50 mL) and evaporated to dryness under reduced pressure. The crude product **11** was purified by column chromatography (SiO_2_, hexane: ethyl acetate = 70:30) as a colourless oil in 70% yield. ^1^H NMR (CDCl_3_, 400 MHz): δ = 8.18 (d, 2H, *J* = 8.7 Hz, H_§_), 7.44 (d, 2H, *J* = 8.7 Hz, H_¥_), 3.64 (s, 2H, H_2_), and 1.44 (s, 9H, H_1_) ppm. ^13^C NMR (CDCl_3_, 100 MHz): δ = 28.1, 42.5, 81.9, 123.8, 130.3, 142.2, 147.1, and 169.5 ppm. ESI-MS (+): *m*/*z*= 238.2 [M + H]^+^.

#### 3.2.3. Synthesis of Tert-Butyl 2-(4-aminophenyl)acetate (**13**)

To a solution of **11** (1.8 g, 7.6 mmol) in methanol (20 mL) kept under a hydrogen atmosphere, a tip of a spatula of Pd/C was added. After stirring at room temperature for 12 h, the solution was vacuum filtered over celite in an inert atmosphere. The solvent was evaporated under reduced pressure, and the residue was portioned between dichloromethane and water. The separated organic phase was dried over CaCl_2_, filtered, and evaporated to dryness to quantitatively afford **13** as a colourless oil. ^1^H NMR (CDCl_3_, 400 MHz): δ = 7.05 (d, 2H, *J* = 8.4 Hz, H_¥_), 6.64 (d, 2H, *J* = 8.4 Hz, H_§_), 3.59 (bs, 2 H, Ar*NH_2_*), 3.40 (s, 2H, H_2_), and 1.43 (s, 9H, H_1_) ppm. ^13^C NMR (CDCl_3_, 100 MHz): δ = 28.2, 41.9, 80.6, 115.4, 124.8, 130.2, 145.3, and 171.7 ppm. ESI-MS (+): *m*/*z*= 208.1 [M + H]^+^.

#### 3.2.4. General Procedure for the Synthesis of Calix[6]arenes **TPU-ES** and **TPU-OTBS**

In a nitrogen atmosphere, a solution of triphosgene (103.1 mg, 347.3 µmol, mg, 1.1 eq.) in toluene (10 mL) was poured into a 250 mL two-necked flask. A freshly prepared solution of triamino calix[6]arene **TA** (350 mg, 315.7 µmol, 1 eq.) and triethylamine (154 μL, 1.1051 mmol, 3.5 eq.) in toluene (20 mL) was added to the reactor. The mixture was stirred at 80 °C for 3 h. After cooling the reactor at room temperature, compound **12** or **14** was added, and the reaction was stirred at room temperature for 12 h. After the completion of the reaction, the solvent was evaporated under reduced pressure. The crude product was purified by column chromatography (SiO_2_, hexane: ethyl acetate = 70:30).

**TPU-ES**: the product was recovered in 72% yield as a white solid. M.p. = 183–185 °C. ^1^H NMR (CDCl_3_, 400 MHz): δ = 7.4–7.2, 7.1–7.0, 6.8, 6.3 (6 br. s, 24H, N*H* and Ar*H*), 4.4 (br. s, 6H, H*_ax_*), 4.1 (br. s, 6H, H_d_), 3.84 (m, 6H, H_e_), 3.7–3.3 (m, 18H, H_f_, H_c_ and H*_eq_*), 3.1–2.5 (br. s, 9H, -OC*H_3_*), 1.42 (s, 27H, H_j_), and 1.4–1.1 (m, 36H, H_i_, H_g_) ppm. ^13^C NMR (CDCl_3_, 100 MHz): δ = 129.7, 72.5, 69.9, 67.0, 42.2, 42.0, 34.4, 31.7, 31.6, 29.8, 28.2, and 15.4 ppm. HR-MS (ESI, Orbitrap LQ) calculated for C_108_H_139_N_6_O_18_: *m*/*z* (z = 1): 1808.01404, 1809.01739, 1810.02075, 1811.02410, and 1812.02746; found 1808.01286, 1809.01598, 1810.01902, 1811.02156, and 1812.02503. For a complete structural assignment, see also Figures S11–S16, SM.

**TPU-OTBS**: the product was recovered in 64% yield as an orange solid. M.p. = 158–160 °C. ^1^H NMR (CDCl_3_, 400 MHz): δ = 7.2, 7.1, 6.2 (3 br. s, 24H, Ar*H* and N*H*), 4.4 (br. s, 6H, H_c_), 4.1 (br. s, 6 H, H*_ax_*), 3.8 (br. s, 6H, H_d_), 3.4 (br. s, 6H, H_e_), 3.7–3.6 (m, 12H, H_f_, H*_eq_*), 2.8 (br. s, 9H, -OC*H_3_*), 1,4–1.0 (m, 36H, H_i_, H_g_), 0.91 (s, 27H, H_j_), and 0.05 (s, 18H, H_k_) ppm. ^13^C NMR (CDCl_3_, 100 MHz): δ = 155.0, 152.2, 146.9, 137.2, 136.8, 135.9, 133.1, 127.8, 127.0, 123.3, 120.7, 72.5, 69.9, 67.0, 64.8, 60.4, 34.3, 31.7, 26.1, 18.4, 15.43, and 5.06 ppm. HR-MS (ESI, Orbitrap LQ) calculated for C_111_H_157_N_6_O_15_Si_3_: *m*/*z* (z = 1): 1898.10092, 1899.10428, 1900.10763, 1901.11099, and 1902.11434; found 1898.10054, 1899.10317, 1900.10381, 1901.10644, and 1902.11069. For a complete structural assignment, see also Figures S17–S22, SM.

#### 3.2.5. General Procedure for the Synthesis of [3]Rotaxanes R[(**TPU**)_2_⊃6_12_], R[(**TPU**)_2_⊃7_12_] and R[(**TPU-ES**)_2_⊃7_12_]

A suspension of bis-viologen axle (**4_12_** or **5_12_**, 0.03 mmol) and wheel (**TPU** or **TPU-ES**, 0.06 mmol) in toluene (10 mL) was stirred at room temperature until it turned homogeneous and deep red (24 hrs). Triethylamine (0.03 g, 0.12 mmol) and diphenylacetyl chloride (0.03 g, 0.12 mmol) were added. After stirring at room temperature for 16 h, the solvent was evaporated to dryness under reduced pressure. The resulting red solid residue was purified by column chromatography (SiO_2_, CH_2_Cl_2_:CH_3_OH = 95:5). The isolated [3]rotaxane was then dissolved in dichloromethane and washed twice with an aqueous solution of NaOTs and twice with distilled water.

*R*[(**TPU**)_2_⊃**6_12_**] was obtained as a sticky red solid in 15% yield (0.05 g). ^1^H NMR (benzene-d_6_, 400 MHz): δ = 9.4 (br. s, 12H, -NH-), 8.2 (br. d, 12H, *J* = 7.7 Hz, TsO), 8.0, 7.9, 7.8 (3 br. s, 20H, H_¥_, H_a,_ H_§_), 7.58 (s, 12H, H_h_), 7.43 (d, 12H, DPA), 7.2–7.0, 7.03, 6.91, 6.8, 6.7 (m, t, d, br. t, br. d, 42H, *J* = 8 Hz, *J* = 8 hz, (DPA), H_h’_, H_b_, H_#_, TsO, H_c_, H_$_), 5.11 (s, 2H, H_θ_), 4.56 (d, 12H, *J* = 14.8 Hz, H_ax_), 4.3 (br. t, 4H, *J* = 12.8 Hz, H_1_), 3.89, 3.8, 3.7 (s, br. d, 34H, O*CH*_3_, H_d_, H_α_), 3.6, 3.5, 3.5–3.3 (br. s, br. s, m, 40H, H_6_, H_e_, H_f_, H*_eq_*,), 1.97 (s, 12H, TsO), and 1.8, 1.69, 1.6, 1.3, 1.2, 1.11, 1–0.9 (br. s, s, br. s, br. s, br. s, br. s, t, br. s, br. t, 100H, H_i_, H_2–5_, H_β-ζ_ and H_g_) ppm. ^13^C NMR (benzene-d_6_, 100 MHz): δ = 172.4, 153.9, 153.2, 148.3, 144.7, 143.7, 143.3, 141.6, 139.7, 139.4, 137.9, 134.2, 132.5, 129.7, 129.1, 128.9, 126.9, 125.9, 125.3, 121.5, 118.5, 117.0, 72.8, 70.3, 66.7, 65.4, 61.3, 61.1, 60.0, 57.7, 38.6, 34.9, 32.4, 31.9, 31.7, 30.2, 30.1, 30.0, 29.8, 29.7, 29.6, 29.4, 28.1, 26.6, 26.4, 23.1, 21.2, 15.6, and 14.4 ppm. HR-MS (ESI, Orbitrap LQ) calculated for C_266_H_316_N_16_O_34_S_2_: *m*/*z* (z = 2): 2171.14602 (24%), 2171.64769 (70%), 2172.14937 (100%), 2172.65105 (95%), 2173.15273 (68%), 2173.65440 (39%), 2174.15608 (18%), and 2174.65776 (7%); found 271.14348 (21%), 2171.64532 (64%), 2172.14235 (99%), 2172.664861 (100%), 2173.15003 (80%), 2173.65187 (53%), 2174.15217 (30%), and 2174.65623 (13%). For a full structural assignment, see Figures S29–S35, SM.

*R*[(**TPU**)_2_⊃**7_12_**]: was obtained as a sticky red solid in 21% yield (0.07 g) 0.07 g (21%) as a sticky red compound. ^1^H NMR (benzene-d_6_, 400 MHz): δ = 9.3–9.2 (br. s, 12H, -NH), 8.14 (d, 8H, *J* = 8 Hz, TsO), 8.0, 7.9, 7.8 (br. s, br. s, br. s, 20H, H_¥_, H_a_, H_§_), 7.6 (br. s, 12H, H_h_), 7.4 (d, 12H, H_b_), 7.31 (d, 12H, *J* = 7.6 Hz, DPA), 7.1–7.0 (m, 20H, H_h’_, DPA,), 6.91 (d, 8H, *J* = 16 Hz, TsO, H_#_), 6.8 (br. t, 6H, H_c_), 6.7 (br. S, 4H, H_$_), 5.08 (s, 2H, H_θ_), 4.59 (d, 12H, *J* = 14.7 Hz, H*_ax_*), 4.06 (t, 4H, *J* = 6.7 Hz, H_1_), 3.95 (s, 9H, O*CH*_3_), 3.86 (br. s, 34H, H_d_ and H_α_), 3.7 (br. s, 4H, H_12_), 3.6 (br. s, 12H, H_e_), 3.5–3.2 (m, 18H, H*_eq_* and H_f_), 2.1 (br. s, 4H, H_11_), 1.96 (s, 12 H, TsO), and 1.9, 1.68, 1.6, 1.6–0.9, 1.14 (br. s, br. s, br. s, m, br. t, 110H, H_2–10_, H_β-ζ_, Hi_,_ and H_g_) ppm. ^13^C NMR (benzene-d_6_, 100 MHz): δ = 177.2, 172.4, 153.9, 153.2, 148.5, 148.4, 144.8, 143.4, 143.3, 141.5, 140.5, 139.9, 139.5, 139.1, 137.8, 134.2, 132.6, 129.9, 129.7, 129.2, 129.1, 129.0, 128.9, 128.8, 127.5, 127.4, 126.9, 125.9, 125.3, 121.5, 118.5, 117.1, 72.8, 70.4, 66.7, 65.1, 61.9, 61.3, 57.7, 57.5, 34.9, 32.3, 31.9, 31.2, 30.6, 30.4, 30.3, 30.2, 30.1, 30.0, 29.8, 29.6, 29.4, 28.9, 26.6, 26.2, 23.1, 21.1,21.0, 15.6, and 14.4 ppm. HR-MS (ESI, Orbitrap LQ) calculated for C_278_H_340_N_16_O_34_S_2_: *m*/*z* (z = 2): 2255.23992 (22%), 2255.74160 (67%), 2256.24327 (100%), 2256.74495 (99%), 2257.246632 (74%), 2257.74830 (44%), 2258.24998 (22%), and 2258.75166 (9%); found 2255.23668 (18%), 2255.74953 (58%), 2256.24190 (93%), 2256.74351 (100%), 2257.246381 (83%), 2257.74418 (59%), 2258.24485 (34%), and 2258.74509 (17%). For a full structural assignment, see also Figures S36–S42, SM.

*R*[(**TPU-ES**)_2_⊃**7_12_**] was obtained as a sticky red solid in 24% yield (0.04 g). ^1^H NMR (benzene-d_6_, 400 MHz): δ = 9.3 (2 br. s, 12H, -NH), 8.14 (d, 8H, *J* = 8 Hz, TsO), 7.9, 7.8, 7.7 (3 br. s, 20H, H_¥_, H_a_, H_§_), 7.58 (br. s, 12H, H_h_), 7.40 (d, 10H, *J* = 7.5 Hz, DPA), 7.32 (d, 10H, *J* = 7.6 Hz, DPA), 7.2–7.0 (m, 28H, H_b_, H_#_ and H_h’_), 6.91 (d, 8H, *J* = 16 Hz, TsO), 6.7 (br. S, 4H, H_$_), 5.08 (s, 2H, H_θ_), 4.63 (d, 12H, *J* = 15 Hz, H*_ax_*), 4.06 (t, 4H, *J* = 7 Hz, H_1_), 3.95 (s, 9H, O*CH*_3_), 3.9, 3.8, 3.6, (3 br. s, 32H, H_d_, H_α_, H_12_ and H_e_), 3.5–3.4 (m, 40H, H_f_, H*_eq_* and H_c_), 1.98 (s, 12H, TsO), 1.73 (s, 27H, H_j_), and 1.6–1.0, 1.44, 1.15 (m, s, t, 165H, H_2–_11, Hi, H_β-ζ_, and H_g_) ppm. ^13^C NMR (benzene-d_6_, 100 MHz): δ = 129.9, 129.4, 128.8, 128.8, 128.7, 128.6, 128.5, 127.8, 127.5, 127.2, 127.2, 126.6, 118.1, 70.1, 66.6, 66.5, 64.8, 61.0, 57.4, 31.7, 28.7, 28.0, 27.8, 25.9, 20.9, and 15.3 ppm. HR-MS (ESI, Orbitrap LQ) calculated for C_300_H_386_N_16_O_40_ [M-4OTs]: *m*/*z* (z = 4): 1213.21601, 1213.46685, 1213.71769, 1213.96853, 1214.21936, 1214.47020, 1214.72104, 1214.97188, and 1215.22272; found 1213.21960, 1213.47004, 1213.72126, 1213.97215, 1214.22240, 1214.47295, 1214.72394, 1214.97433, and 1215.22577. For a complete structural assignment, see also Figures S54–S60, SM.

#### 3.2.6. General Procedure for the Synthesis of [3]Rotaxanes R[(**TPU**)_2_⊃**8_12_**] and R[(**TPU-ES**)_2_⊃**8_12_**]

A suspension of bis-viologen axle **5_12_** (0.03 mmol) and wheel (**TPU** or **TPU-ES**, 0.06 mmol) in toluene (10 mL) was stirred at room temperature until it turned homogeneous and deep red (24 h). Imidazole (0.009 g, 0.12 mmol) and chlorotriphenylsilane (0.035 g, 0.12 mmol) were then added in this order. After stirring at room temperature for 48 h, the solvent was evaporated to dryness under reduced pressure. The resulting red solid residue was purified by column chromatography (SiO_2_, CH_2_Cl_2_:CH_3_OH = 95:5). The isolated [3]rotaxane was then dissolved in dichloromethane and washed twice with an aqueous solution of NaOTs and twice with distilled water.

*R*[(**TPU**)_2_⊃**8_12_**] was obtained as a sticky red solid in 14% yield (0.015 g). ^1^H NMR (benzene-d_6_, 400 MHz, 25 °C): δ = 9.3 (br. s, 12H, -NH), 8.17 (d, 8H, *J* = 8 Hz, TsO), 8.1–7.7 (2 m, 38H, TPS, H_a_, H_§_ and H_¥_), 7.58 (s, 12H, H_h_) 7.3–7.2 (m, 20H, TPS and H_b_), 7.1 (br. s, 10H, H_h’_), 6.92 (d, 8H, *J* = 8 Hz, TsO), 6.8–6.7 (m, 16H, H_#_ and H_$_), 4.57 (d, 12H, *J* = 14 Hz, H*_ax_*), 3.94 (s, 9H, OC*H*3) 3.9 (br. s, 16H, H_1_, H_d_), 3.7 (br. s, 4H, H_α_), 3.6 (br. s, 16H, H_12_ and H_e_), 3.5–3.2 (m, 24H, H_f_ and H*_eq_*), 2.1 (br. s, 4H, H_11_), 1.98 (s, 12H, TsO), and 1.9–0.8 (br. s, s, 5 br. s, t, br. s, 128H, H_2–10_, Hi, H_g_, and H_β-ζ_) ppm. ^13^C NMR (benzene-d_6_, 100 MHz): δ = 144.3, 143.1, 135.6, 135.2, 130.1, 129.4, 129.0, 128.7, 128.0, 126.6, 125.6, 124.8, 121.3, 118.1, 72.5, 70.1, 66.4, 63.9, 61.0, 60.9, 32.9, 31.6, 30.2, 30.0, 29.7, 29.3, 29.0, 26.3, 26.1, 20.9, and 15.3 ppm. HR-MS (ESI, Orbitrap LQ) calculated for C_272_H_334_N_16_O_26_Si_2_ [M-4OTs]: *m*/*z* (z = 4): 1074.12055, 1074.37133, 1074.62209, 1074.87281, 1075.12351, 1075.37418, 1075.62485, 1075.87550, and 1076.12614; found 1074.12160, 1074.37233, 1074.62274, 1074.87341, 1075.12404, 1075.37483, 1075.62526, 1075.87554, and 1076.12609. Calculated for C_279_H_341_N_16_O_29_SSi_2_ [M-3OTs]: *m*/*z* (z = 3): 1489.16477, 1489.49916, 1489.83346, 1490.16771, 1490.50191, 1490.83607, 1491.17020, 1491.50432, and 1491.83841; found 1489.16582, 1489.50006, 1489.83480, 1490.16818, 1490.50235, 1490.83659, 1491.17000, 1491.50462, and 1491.83886. Calculated for C_286_H_348_N_16_O_32_S_2_Si_2_ [M-2OTs]: *m*/*z* (z = 2): 2319.25323, 2319.75480, 2320.25623, 2320.75754, 2321.25876, 2321.75991, 2322.26100, and 2322.76211; found 2319.25395, 2319.75584, 2320.25763, 2023.75902, 2321.26003, 2321.76056, 2322.26079, and 2322.76104. For a complete structural assignment, see also Figures S43–S51, SM.

*R*[(**TPU-ES**)_2_⊃**8_12_**] was obtained as a sticky red solid in 17% yield (0.029 g). ^1^H NMR (benzene-d_6_, 400 MHz): δ = 9.3 (2 br. s, 12H, -NH), 8.12 (d, 8H, *J* = 8 Hz, TsO), 7.9, 7.8, 7.8–7.7 (2 br. s, m, br. s, 58H, H_¥_, H_a_, TPS and H_§_), 7.59 (s, 12H, H_h_), 7.2 (m, 16H, TPS and H_b_), 7 (br. s, 16H, H_h’_ and H_#_), 6.91 (d, 8H, *J* = 8 Hz, TsO), 6.7 (br. d, 4H, H_$_), 4.62 (d, 12H, *J* = 14, H*_ax_*), 3.94 (s, 9H, OC*H*_3_) 3.9–3.8 (m, 20H, H_α_, H_1_ and H_d_), 3.63 (s, 12H, H_e_), 3.5–3.2 (m, 40H, H_f,_ H_c_, and H*_eq_*, H_12_), 1.99 (s, 12H, TsO), 1.9–1.2 (m, 168H, H_i_, H_β-ζ,_ H_2–11_, and H_j_), and 1.15 (t, 18H, *J* = 7 Hz, H_g_) ppm. ^13^C NMR (benzene-d_6_, 100 MHz, 25 °C): δ = 144.6, 143.3, 135.6, 135.2, 130.1, 129.9, 129.4, 128.7, 128.0, 127.9, 126.6, 125.6, 125.2, 118.1, 72.5, 70.1, 66.5, 63.9, 61.7, 61.0, 41.5, 31.7, 31.2, 30.2, 30.0, 29.8, 29.6, 28.0, 26.1, 20.9, and 15.3 ppm. HR-MS (ESI, Orbitrap LQ) calculated for C_308_H_394_N_16_O_38_Si_2_ [M-4OTs]: *m*/*z* (z = 4): 1245.22267, 1245.47350, 1245.72434, 1245.97518, 1246.22602, 1246.47686, 1246.72770, and 1247.22937; found 1245.21594, 1245.46924, 1245.72095, 1245.97229, 1246.22278, 1246.72473, and 1247.22192. Calculated for C_315_H_401_N_16_O_41_SSi_2_ [M-3OTs]: *m*/*z* (z = 3): 1717.30093, 1717.63538, 1717.96984, 1718.30429, 1718.63874, 1718.97319, 1719.30764, 1719.64209, and 1719.97655; found 1717.29626, 171763025, 171796521, 1718.29895, 1718.96716, 1719.30078, 1719.63586, and 1719.96838. Calculated for C_322_H_408_N_16_O_44_S_2_Si_2_ [M-2OTs]: *m*/*z* (z = 2): 2661.45747, 2661.95915, 2662.46082, 2662.96250, 2663.46418, 2663.96586, 2664.46753, 2664.96921, and 2665.47089; found 2661.44678, 2661.95190, 2662.45239, 2663.45532, 2663.95410, 2664.45728, 2664.95923, and 2665.45752. For a complete structural assignment, see also Figures S61–S69, SM.

#### 3.2.7. Synthesis of [3]Rotaxane R[(**TPU-AC**)_2_⊃**7_12_**]

To a solution of *R*[(**TPU-ES**)_2_⊃**7_12_**] (0.02 g, 0.035 mmol) in 5 ml of dry dichloromethane, *p*-toluenesulfonic acid monohydrate (0.35 mmol) was added, and the resulting reaction mixture was refluxed for 1 hour. After this period, the crude product was purified through column chromatography (SiO_2_, CH_2_Cl_2_:CH_3_OH = 95:5). R[(**TPU-AC**)_2_⊃**7_12_**] was obtained as a sticky red solid in 60% yield (0.011 g). ^1^H NMR (CD_2_Cl_2_, 400 MHz): δ = 8.28 (br. s, 16H, -NH and H_¥_), 7.8–6.9 (m, H, TsO, H_h-h’_, H_a-b_ and H_§_), 6.8 (br. s, 4H, H_#_), 5.7 (br. s, 4H, H_$_), 5.02 (s, 2H, H_θ_), 4.47 (d, 12H, *J* = 14 Hz, H*_ax_*), 4.16 (t, 4H, *J* = 7 Hz, H_1_), 4.1–3.7 (m, 44H, OC*H_3_*, H_d_, H_α_, and H_e_), 3.64 (q, 12H, *J* = 7 Hz, H_f_), 3.6–3.3 (m, 24H, H*_eq_*, and H_c_), 3.0 (br. s, 4H, H_12_), 2.38 (s, 12H, TsO), and 2.2–0.5 (m, 132H, H_2–11_, Hi, H_g_ and H_β-ζ_) ppm. ^13^C NMR (CD_2_Cl_2_, 100 MHz, 25 °C): δ = 143.3, 130.1, 129.3, 128.9, 128.8, 128.7, 127.4, 126.4, 118.2, 117.3, 73.0, 70.3, 66.9, 65.5, 61.7, 57.3, 31.3, 30.6, 30.0, 29.8, 29.5, 29.1, 28.8, 26.0, 21.4, and 15.4 ppm. HR-MS (ESI, Orbitrap LQ) calculated for C_276_H_338_N_16_O_40_ [M-4OTs]: *m*/*z* (z = 4): 1129.12211, 1129.37293, 1129.62374, 1129.87454, 1130.12533, 1130.37611, 1130.62689, and 1130.87766; found 1129.12238, 1129.37272, 1129.62394, 1129.87418, 1130.12532, 1130.37554, 1130.62657, and 1130.87690. Calculated for C_283_H_345_N_16_O_43_S [M-3OTs]: *m*/*z* (z = 3): 1562.50019, 1562.83461, 1563.16899, 1563.50333, 1563.83764, 1564.17192, 1564.50618, and 1564.84042; found 1562.50044, 1562.83428, 1563.16907, 1563.50269, 1563.83620, 1564.17082, 1564.50493, and 1564.83858. Calculated for C_290_H_352_N_16_O_46_S_2_ [M-2OTs]: *m*/*z* (z = 2): 2429.25636, 2429.75799, 2430.25951, 2430.76094, 2431.26231, 2431.76362, 2432.26488, 2432.76611, and 2433.26731; found 2429.25649, 2429.75803, 2430.25933, 2430.76027, 2431.26130, 2431.76212, 2432.26331, 2432.76385, and 2433.26532. For a complete structural assignment, see also Figures S70–S78, SM.

### 3.3. Computational Studies

The geometry of [3]rotaxane *R*[(**TPU-ES**)_2_⊃**7**_12_] was initially minimised with the MMFF94 force field [45], using the Avogadro software [46], and then refined at PM7 level [47] using the MOPAC2016 software [40].

## Data Availability

Not applicable.

## Supplementary Materials

The following supporting information can be downloaded at https://www.mdpi.com/article/10.3390/molecules28020595/s1; copy of NMR and HR-MS spectra of the obtained compounds (Figures S1–S78).

## Sample Availability

Samples of the compounds are available from the authors.
