# Supplementary material for: Calix[6]arene-Based [3]Rotaxanes as Prototypes for the Template Synthesis of Molecular Capsules
Source: Molecules. 2023 Jan 6;28(2):595. doi: 10.3390/molecules28020595 (PMC9864859; doi:10.3390/molecules28020595)
Supplement: Supplementary file 1 [file molecules-28-00595-s001.zip › molecules-2075029-supplementary.pdf]

Supporting Material

Calix[*6*]arene-based [*3*]rotaxanes as prototypes for the template synthesis of molecular capsules

Federica Cester Bonati,[1] Margherita Bazzoni,[1] Caterina Baccini,[1] Valeria Zanichelli,[1] Guido Orlandini,[1] Arturo Arduini,[1] Gianpiero Cera,[1] and Andrea Secchi[1,*]

[1] Dipartimento di Scienze Chimiche, della Vita e della Sostenibilità Ambientale, Università di Parma, Parco Area delle Scienze 17/A, I-43124 Parma, Italy

* Correspondence: andrea.secchi@unipr.it; Tel.: +39-0521 905 409.

Table of Contents

Characterisation of bis-viologen axle 4_{12} ……………………………………………..3
Characterisation of bis-viologen axle 5_{6} ……………………………………………..5
Characterisation of bis-viologen axle 5_{12} ……………………………………………7
Characterisation of 11 ……………………………………………………………..9
Characterisation of 13 ……………………………………………………………..10
Characterisation of TPU-ES …………………………………………………..11
Characterisation of TPU-OTBS ……………………………………………..15
Characterisation of P[(**TPU**)$_2$⊃ **5_6**] ……………………………………………19
Characterisation of P[(**TPU**)$_2$⊃ **5_{12}**] ……………………………………………...21
Characterisation of R[(**TPU**)$_2$⊃ **6_{12}**] ……………………………………………24
Characterisation of R[(**TPU**)$_2$⊃ **7_{12}**] ……………………………………………..28
Characterisation of R[(**TPU**)$_2$⊃ **8_{12}**] ……………………………………………32
Characterisation of P[(**TPU-ES**)$_2$⊃ **5_{12}**] ………………………………………...37
Characterisation of R[(**TPU-ES**)$_2$⊃ **7_{12}**] ………………………………………39
Characterisation of R[(**TPU-ES**)$_2$⊃ **8_{12}**] ………………………………………43
Characterisation of R[(**TPU-Ac**)$_2$⊃ **7_{12}**] ………………………………………48

Characterisation of bis-viologen axle $\mathbf{4_{12}}$

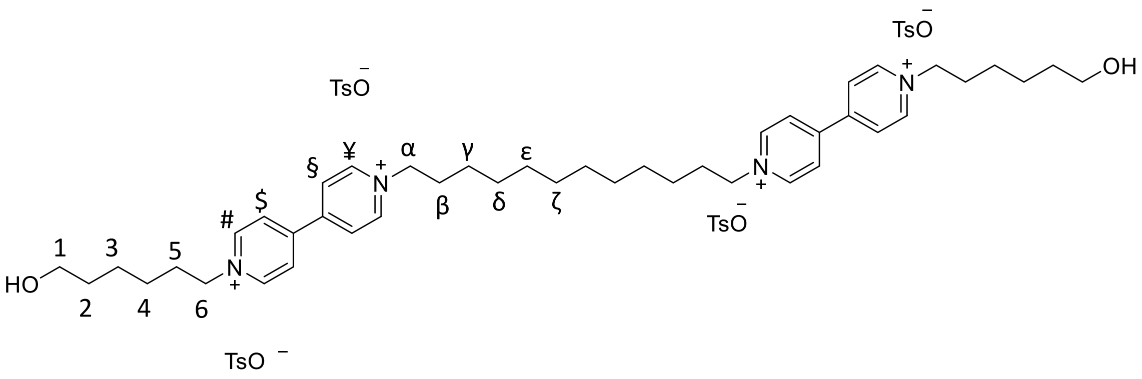

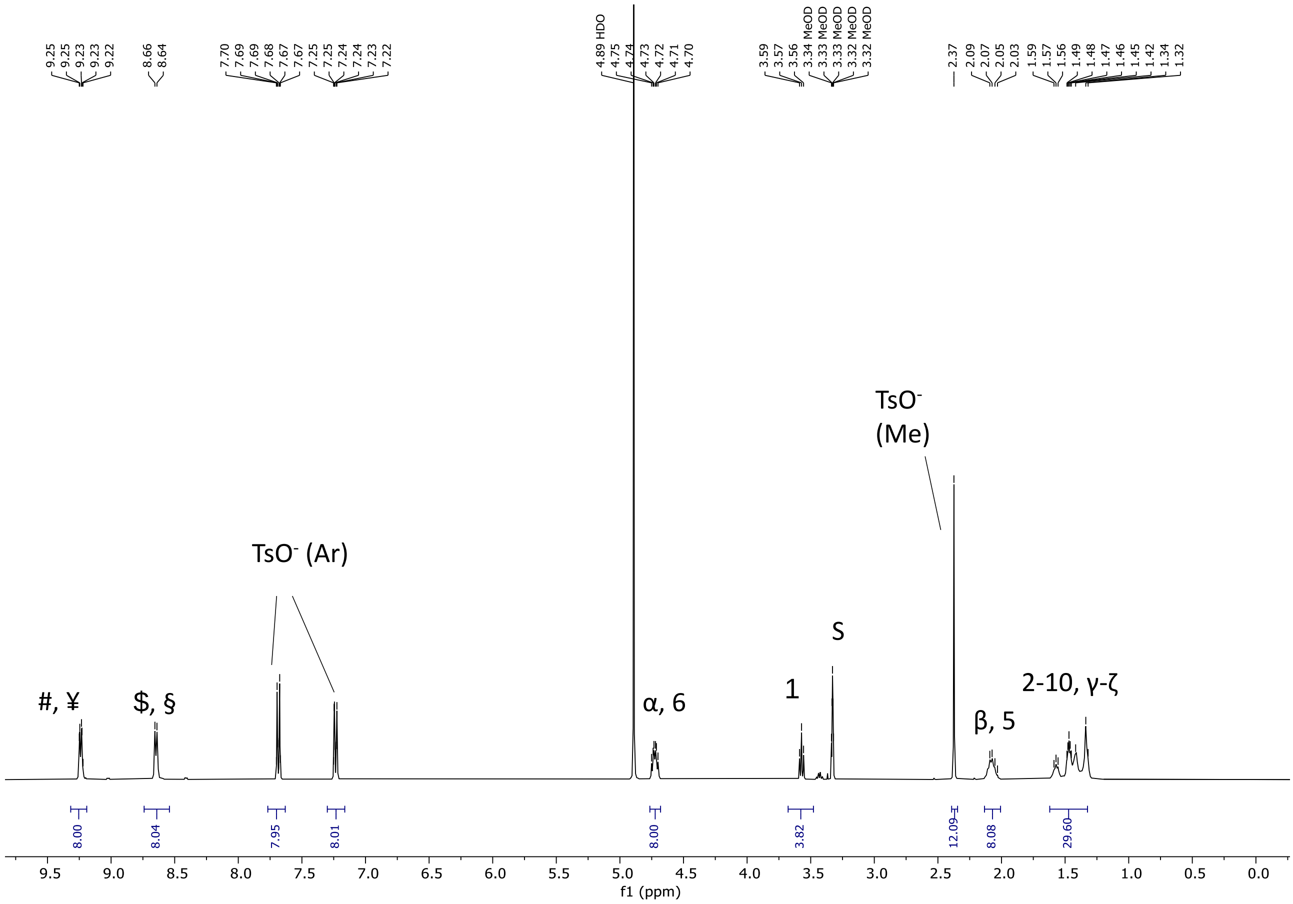

Figure S1: ^{1}H NMR spectrum (400 MHz, CD_3OD) of compound $\mathbf{4_{12}}$.

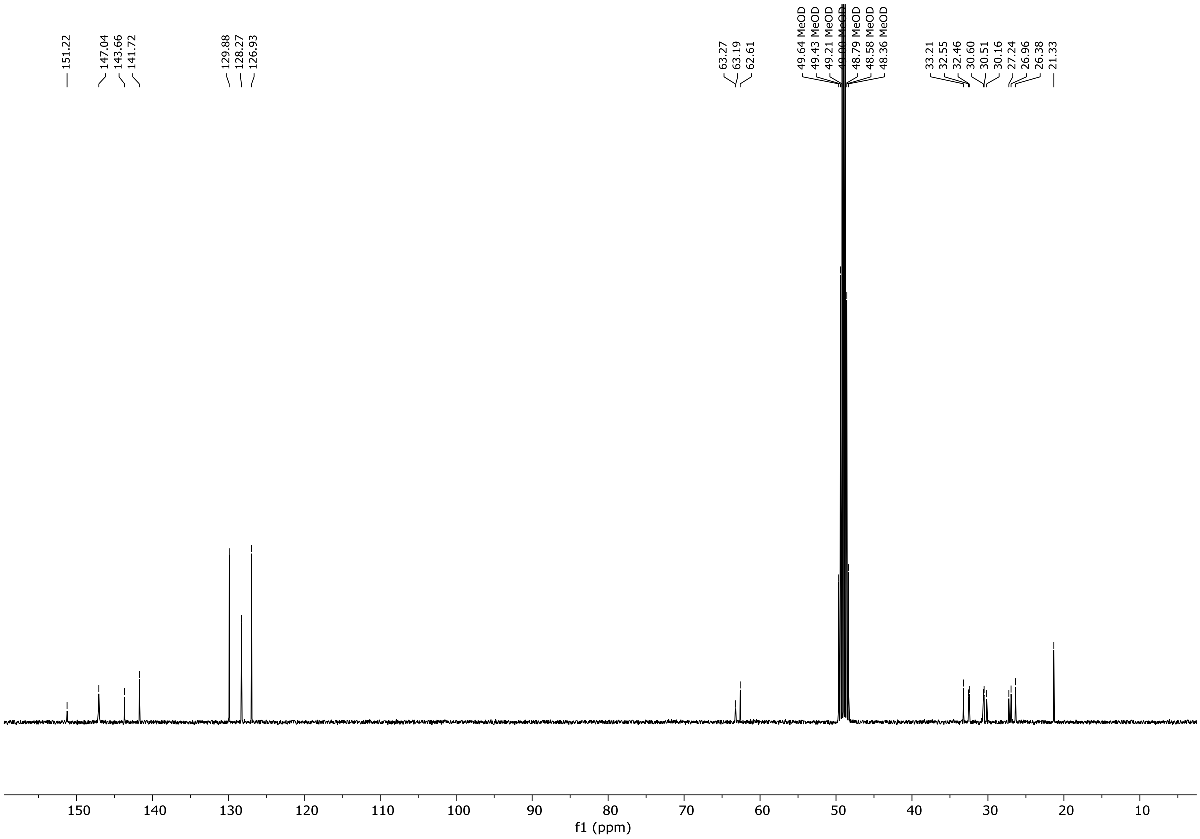

Figure S2: ^{13}C NMR spectrum (100 MHz, CD_3OD) of compound **4**12.

Characterisation of bis-viologen axle 5_6

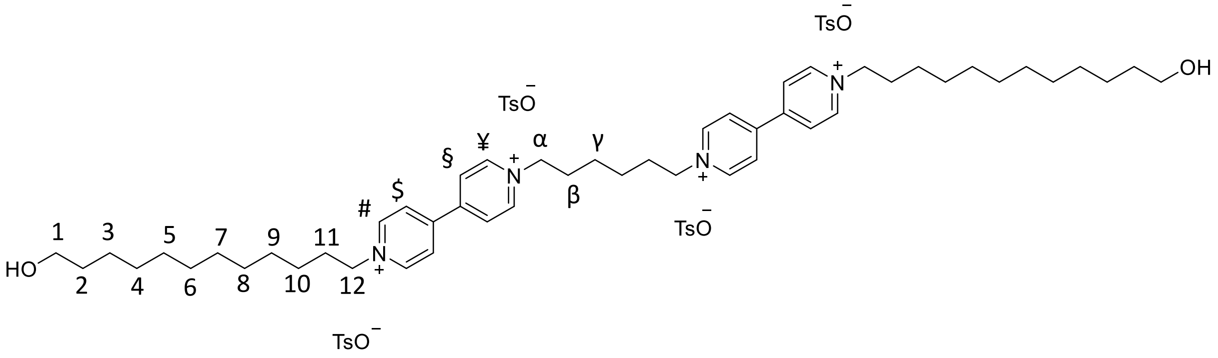

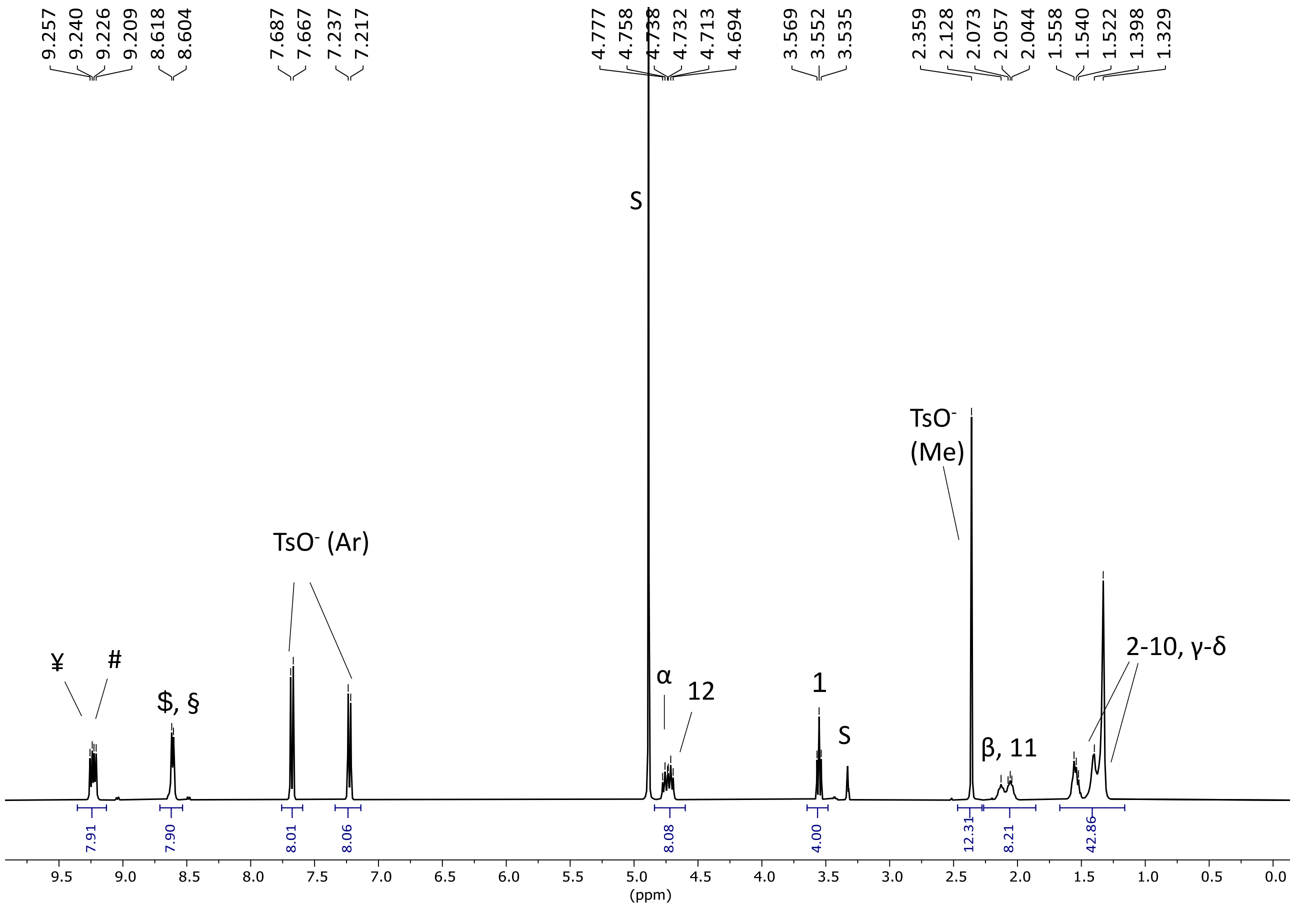

Figure S3: ^{1}H NMR spectrum (400 MHz, CD_3OD) of compound **5_6**.

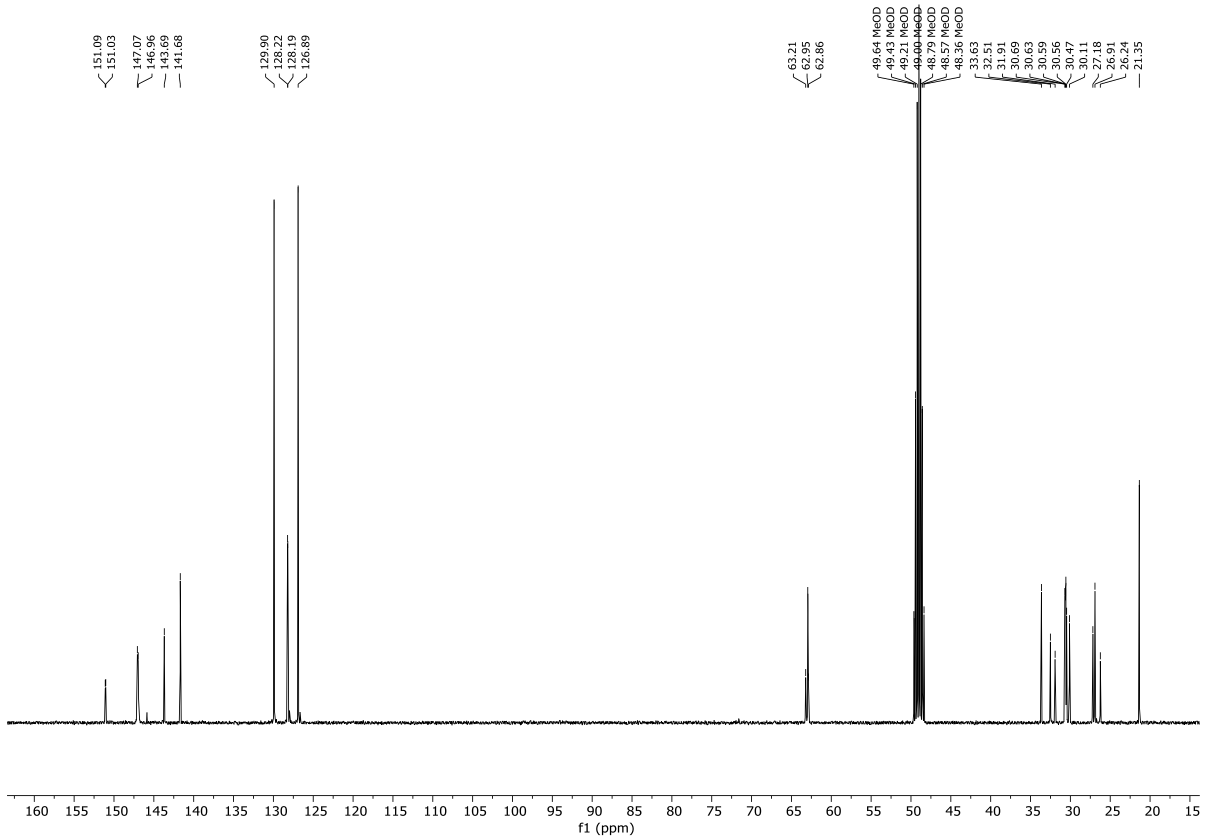

Figure S4: ^{13}C NMR spectrum (100 MHz, CD_3OD) of compound **5_6**.

Characterisation of bis-viologen axle 5_{12}

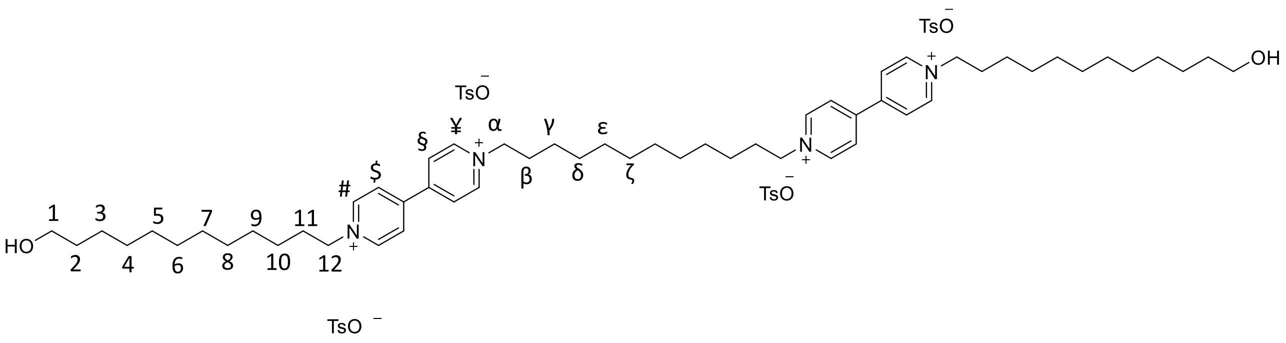

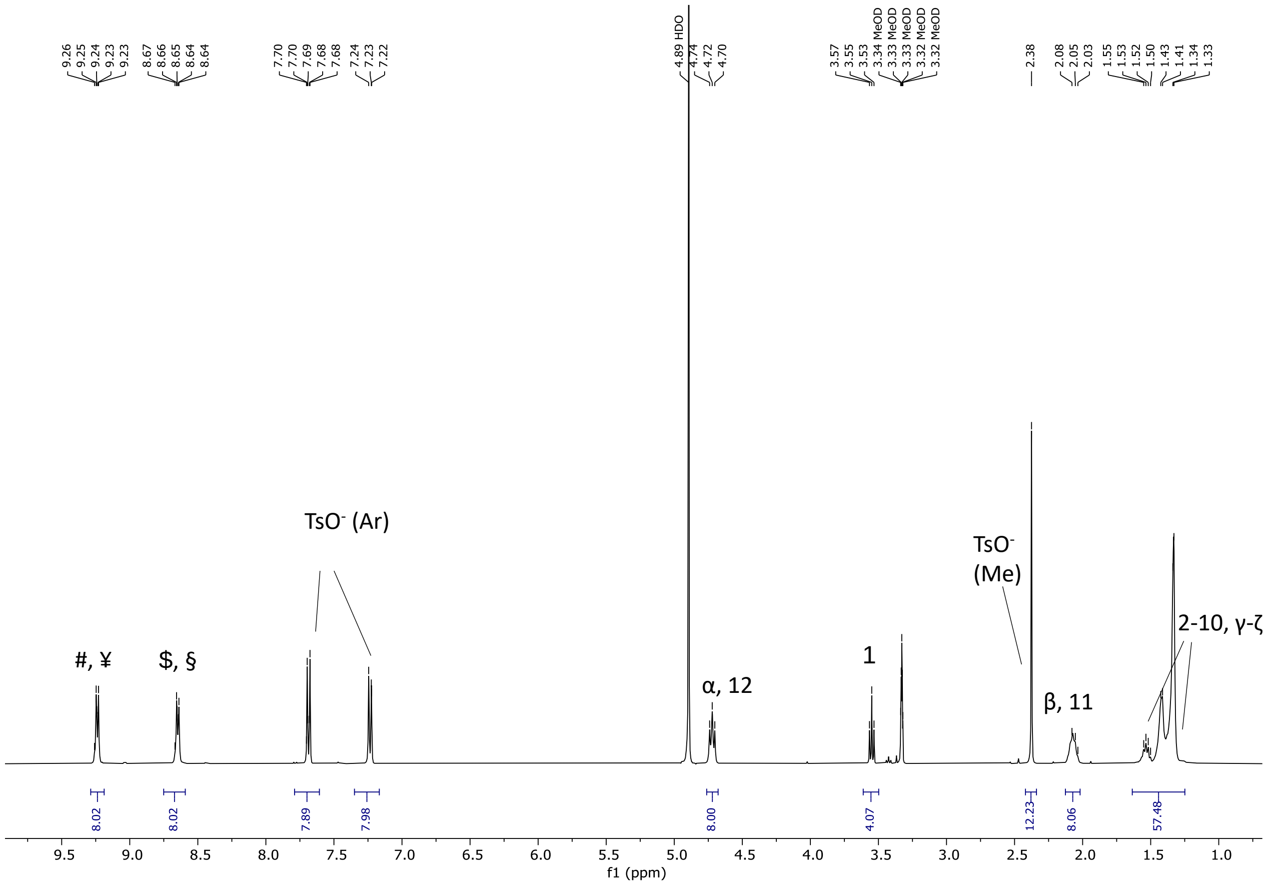

Figure S5: ^{1}H NMR spectrum (400 MHz, CD_3OD) of compound **5**12.

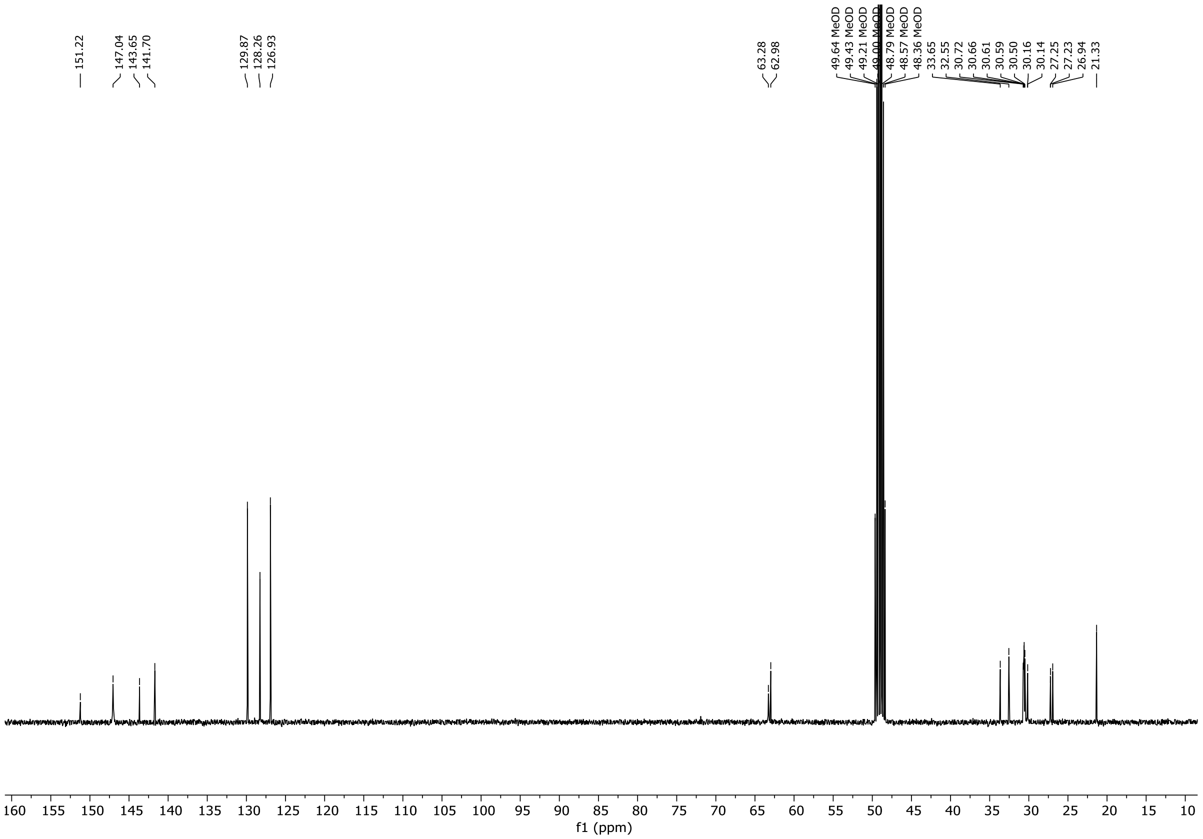

Figure S6: ^{13}C NMR spectrum (100 MHz, CD_3OD) of compound **5**$_{12}$.

Characterisation of 11

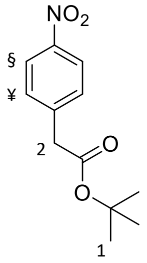

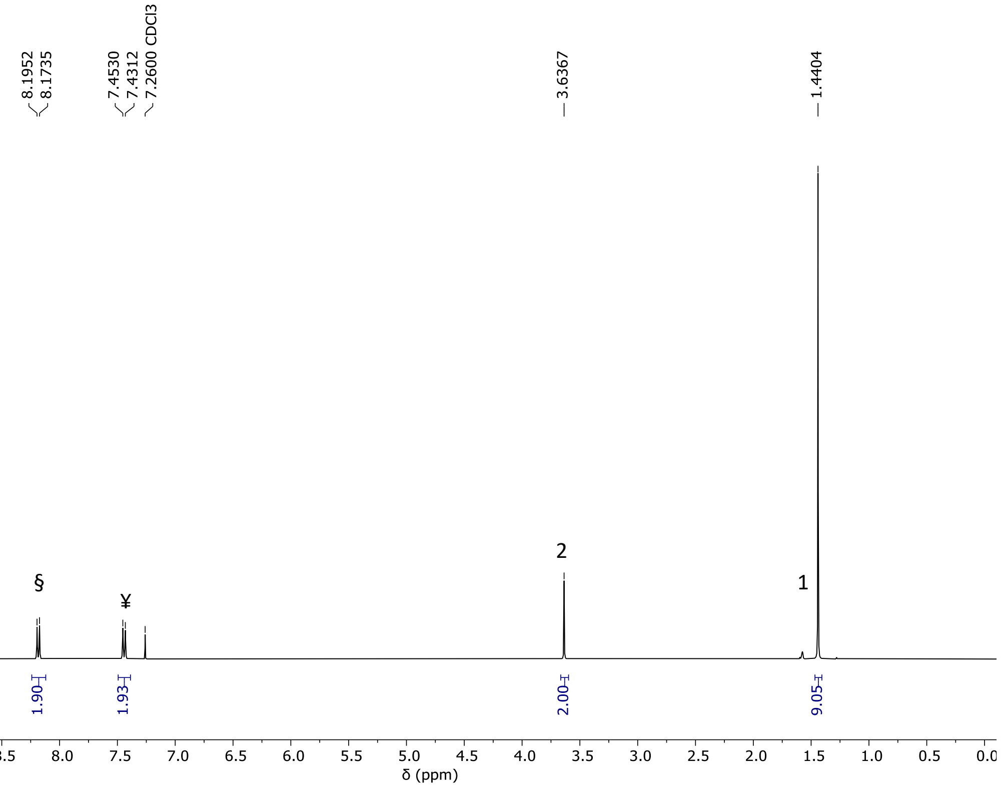

Figure S7: ^{1}H NMR spectrum (400 MHz, $CDCl_3$) of compound **11**.

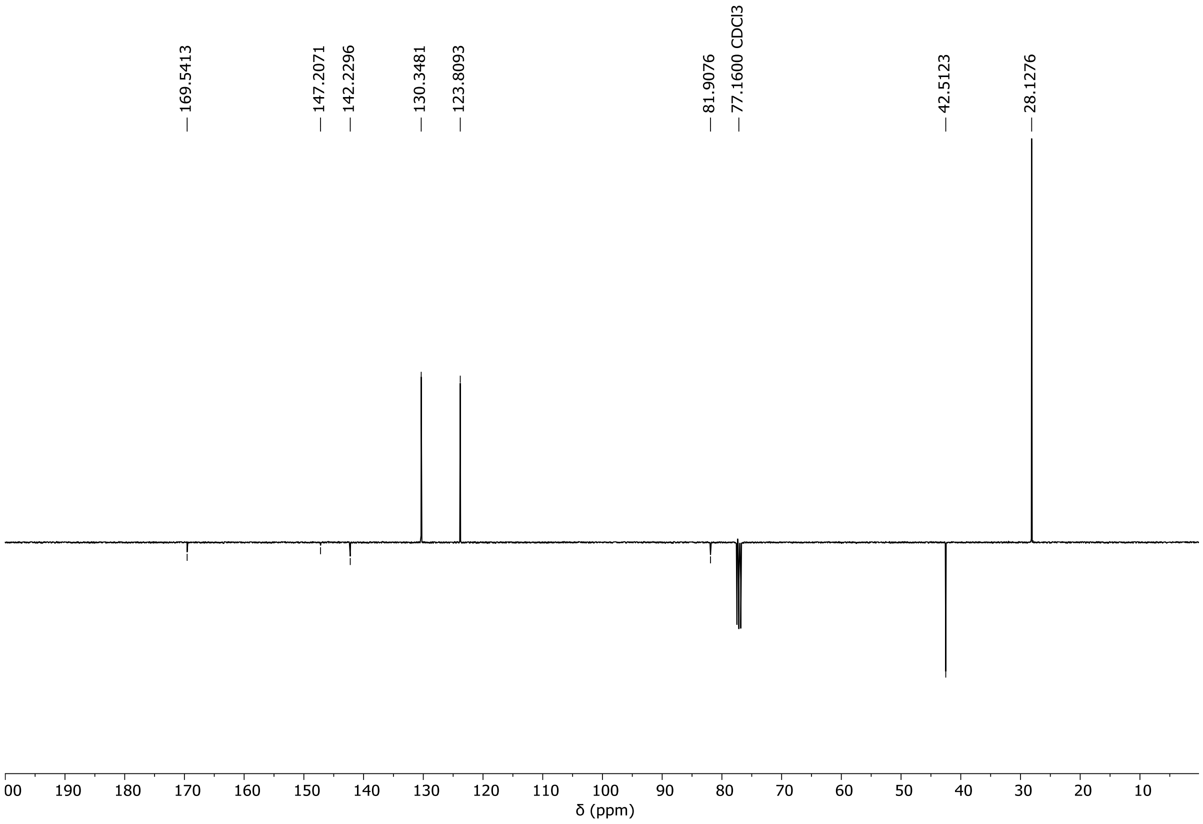

Figure S8: ^{13}C-APT NMR spectrum (100 MHz, $CDCl_3$) of compound **11**.

Characterisation of 13

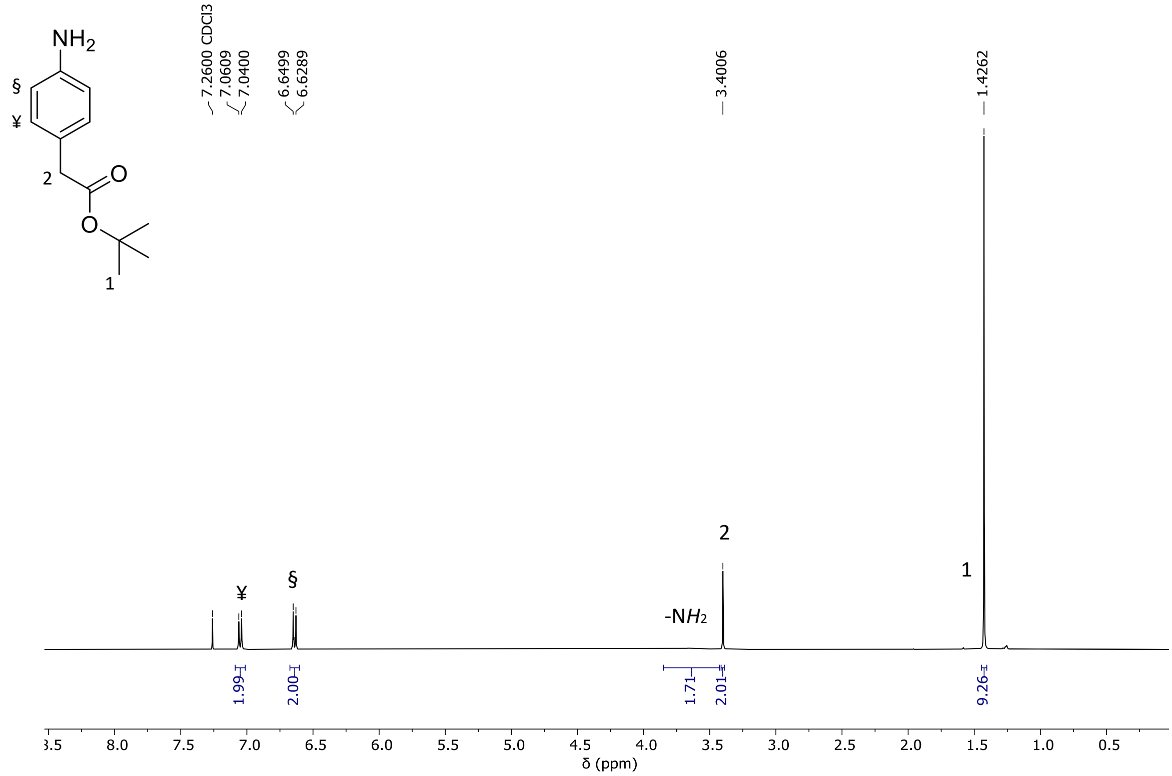

Figure S9: ^{1}H NMR spectrum (400 MHz, $CDCl_3$) of compound **13**.

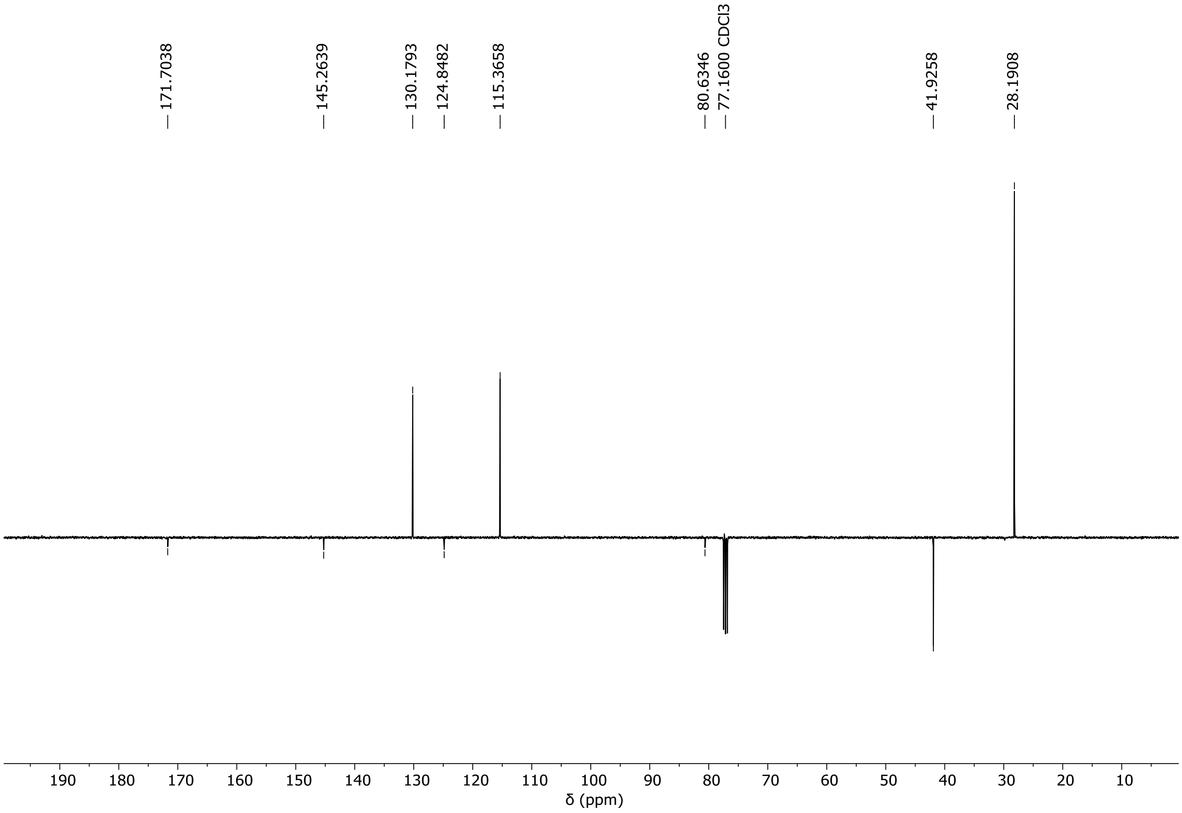

Figure S10: ^{13}C-APT NMR spectrum (100 MHz, $CDCl_3$) of compound **13**.

Characterisation of TPU-ES

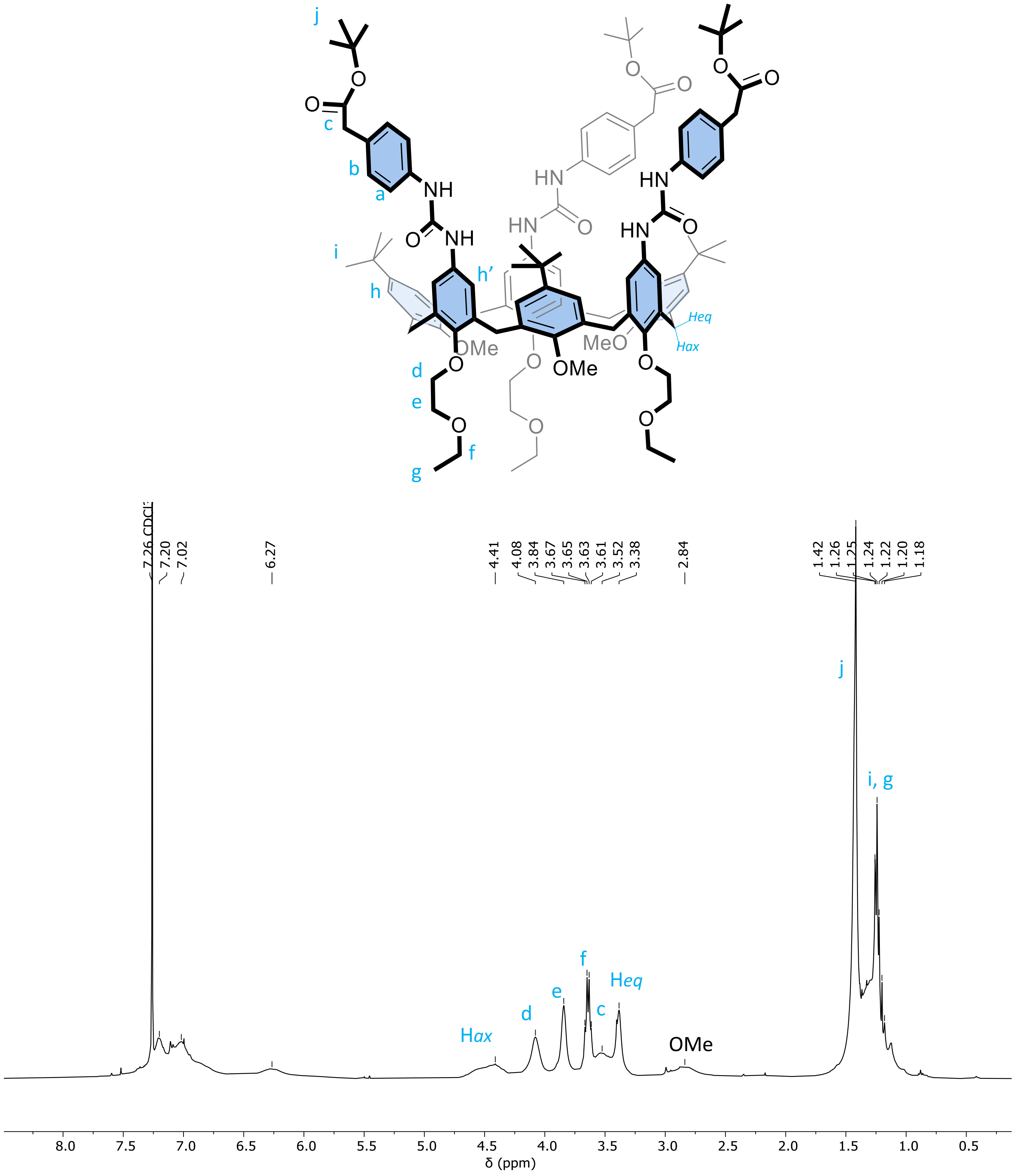

Figure S11 ^{1}H NMR spectrum (400 MHz, $CDCl_3$) of compound **TPU-ES**.

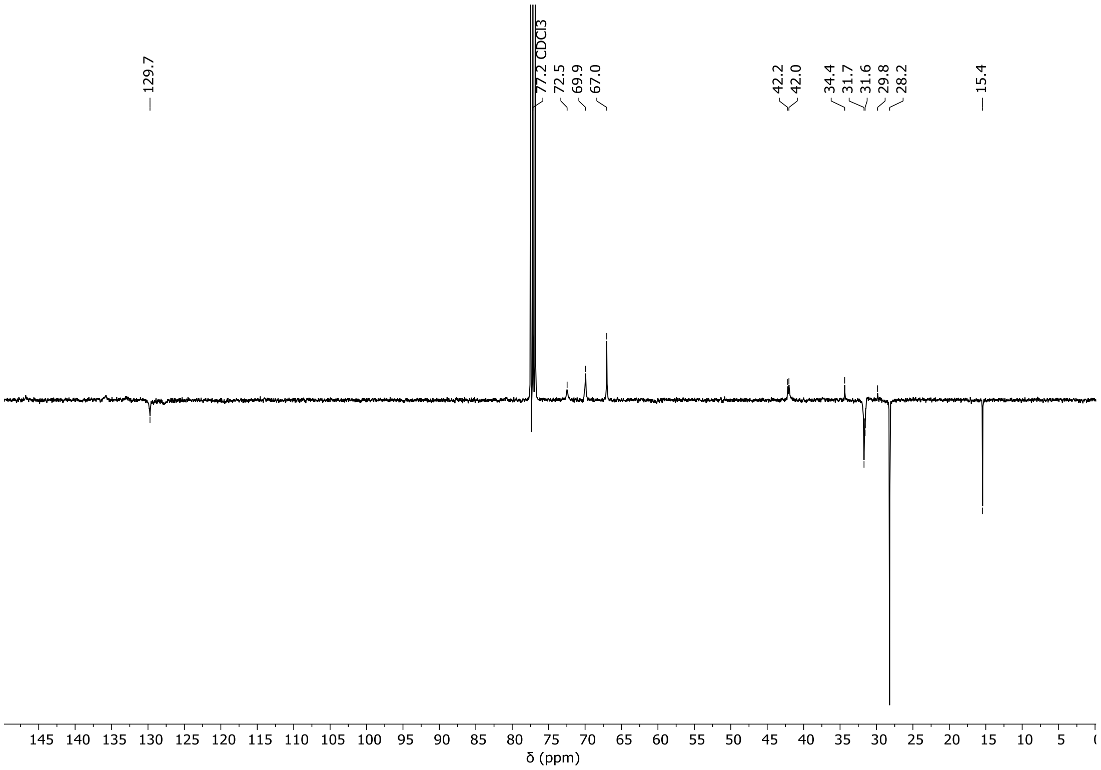

Figure S12: ^{13}C-APT NMR spectrum (100 MHz, $CDCl_3$) of compound **TPU-ES**.

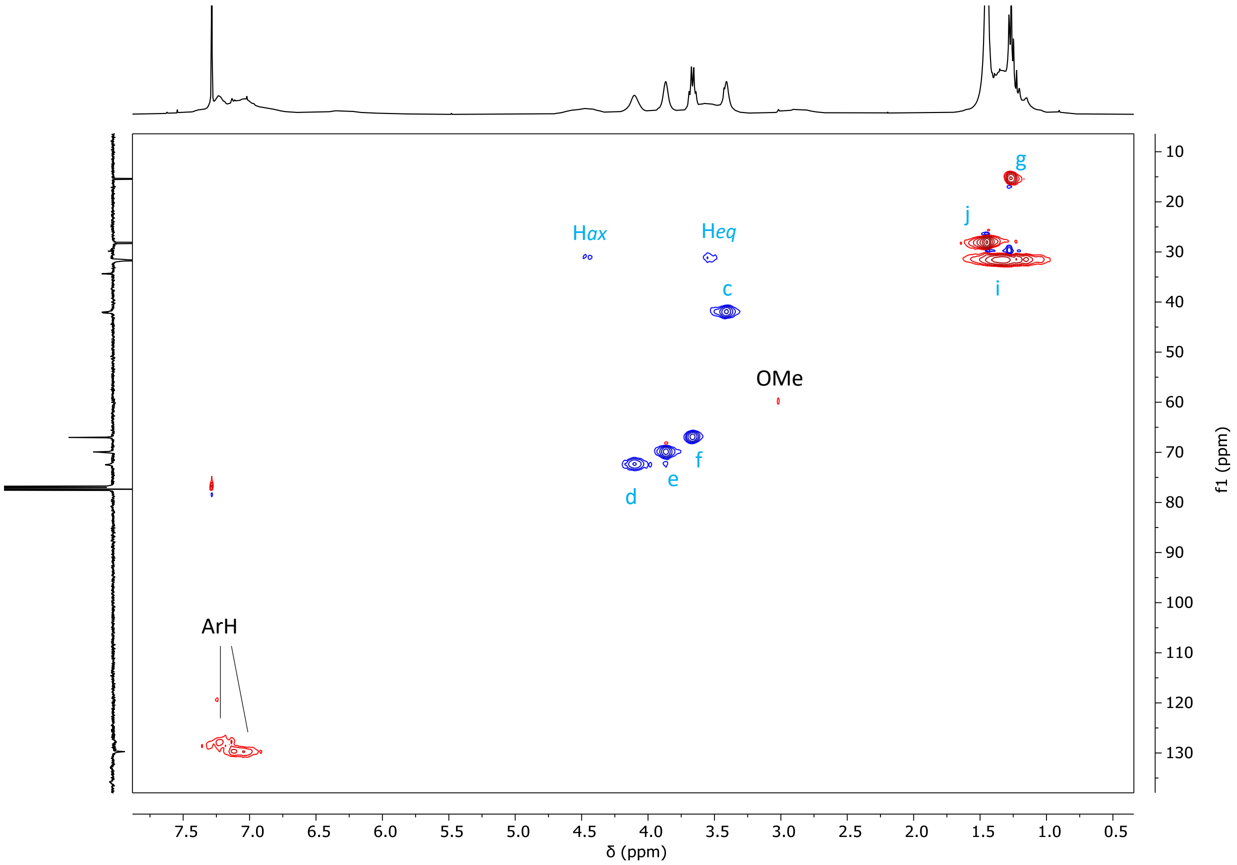

Figure S13: Edited HSQC 2D NMR spectrum (400 MHz, $CDCl_3$) of compound **TPU-ES**. Positive peaks (CH_3 and CH) are shown in red, while negative ones (CH_2) are in blue.

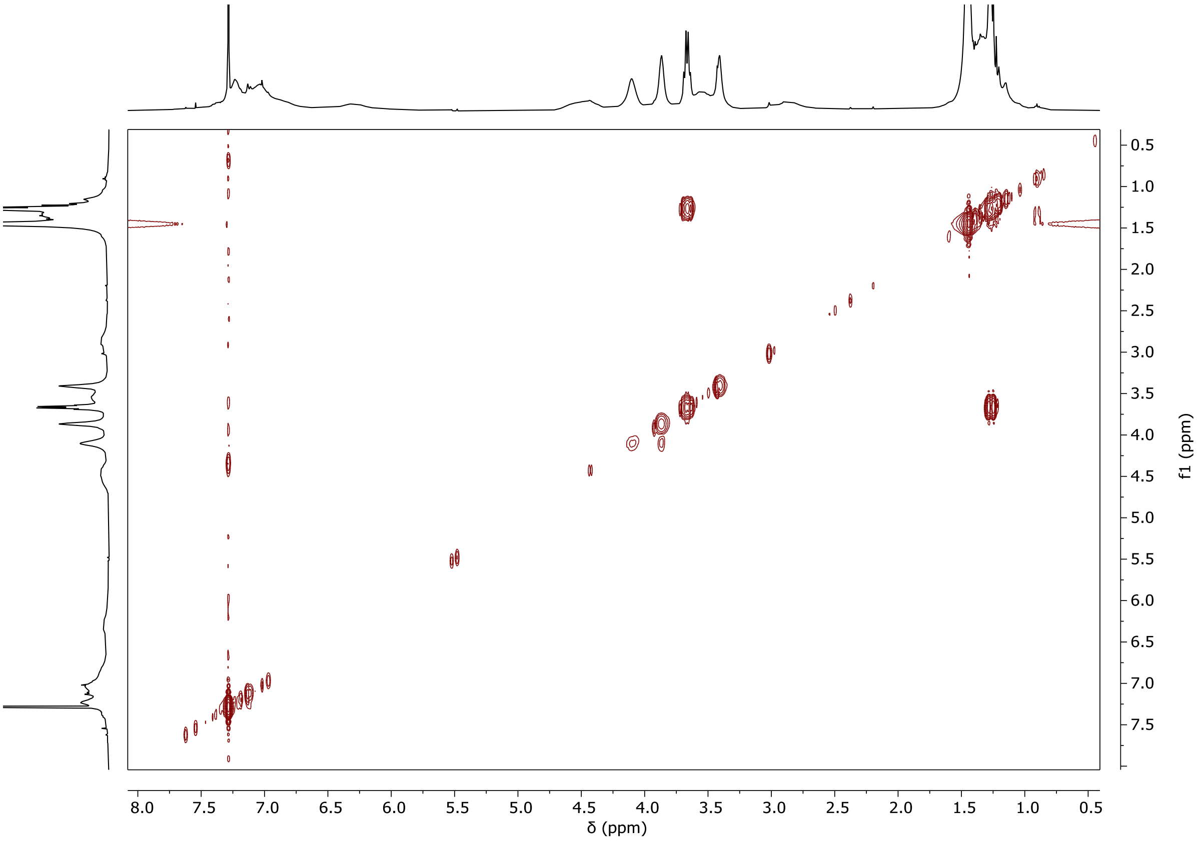

Figure S14: 2D magnitude g-COSY NMR spectrum (400 MHz, $CDCl_3$) of compound **TPU-ES**.

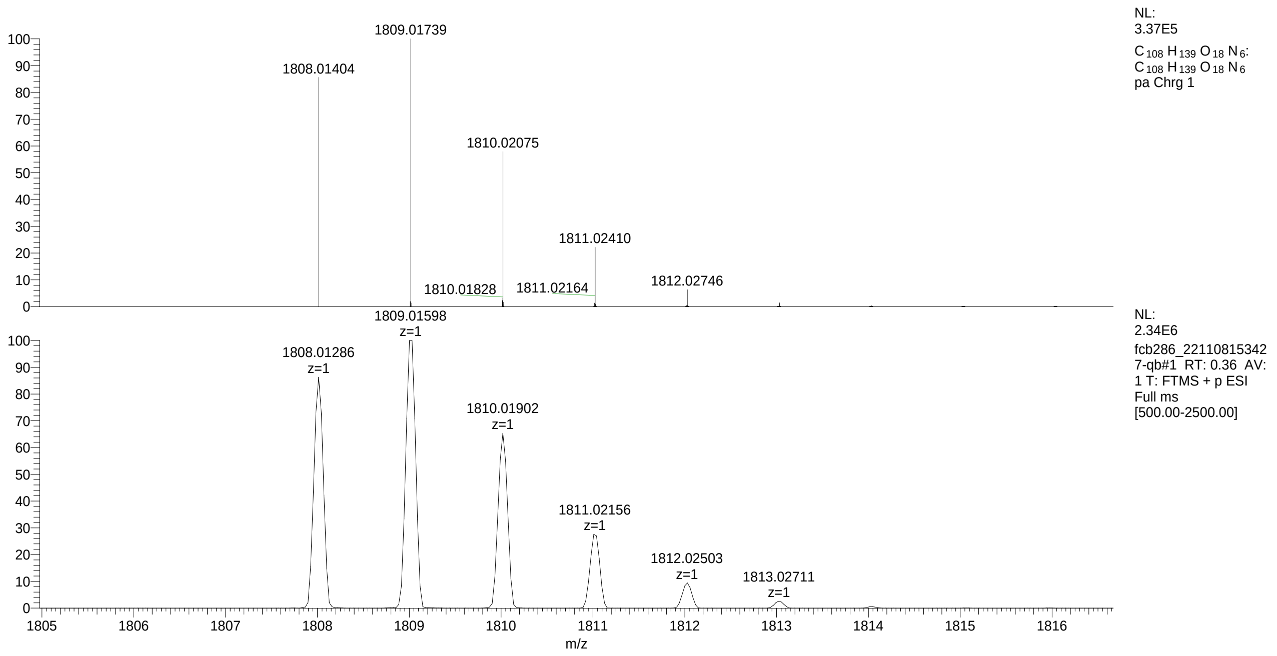

Figure S15: Inset of HR-MS (ESI, Orbitrap LQ) spectrum of compound **TPU-ES:** calculated (top) and experimental (down) isotopic distribution for the singly charged molecular ion.

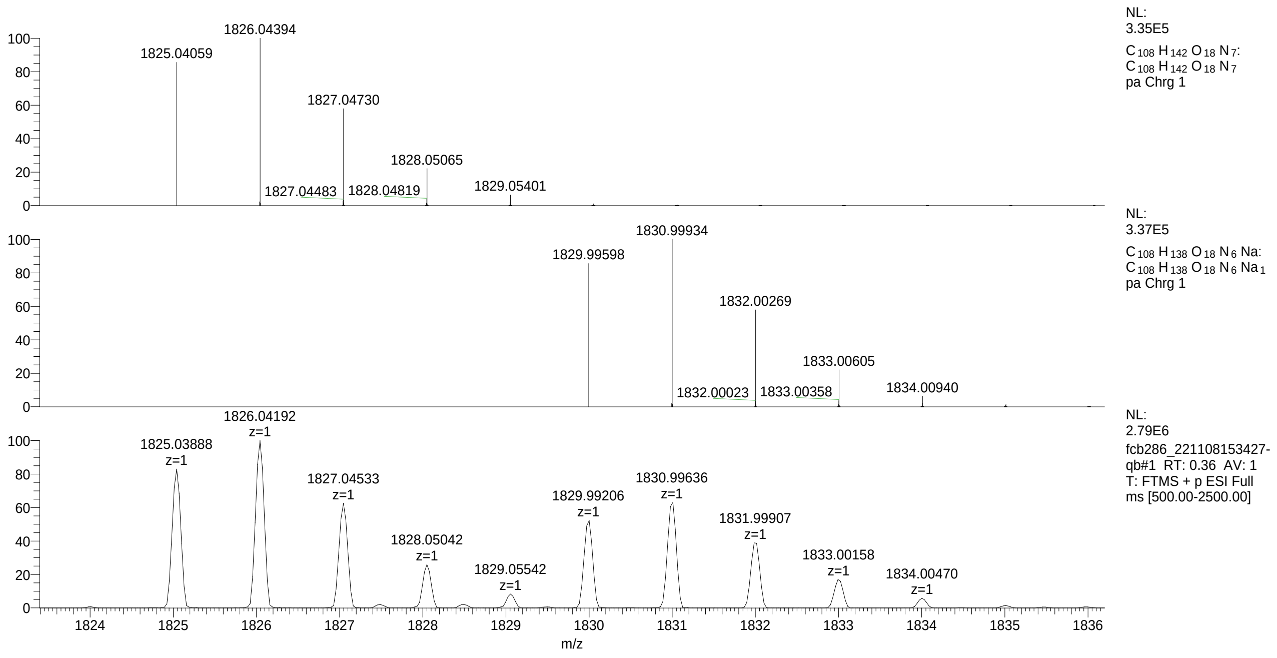

Figure S16: Inset of HR-MS (ESI, Orbitrap LQ) spectrum of compound **TPU-ES:** calculated (top) and experimental (down) isotopic distributions for singly charged molecular adducts with ammonium and sodium ions, respectively.

Characterisation of TPU-OTBS

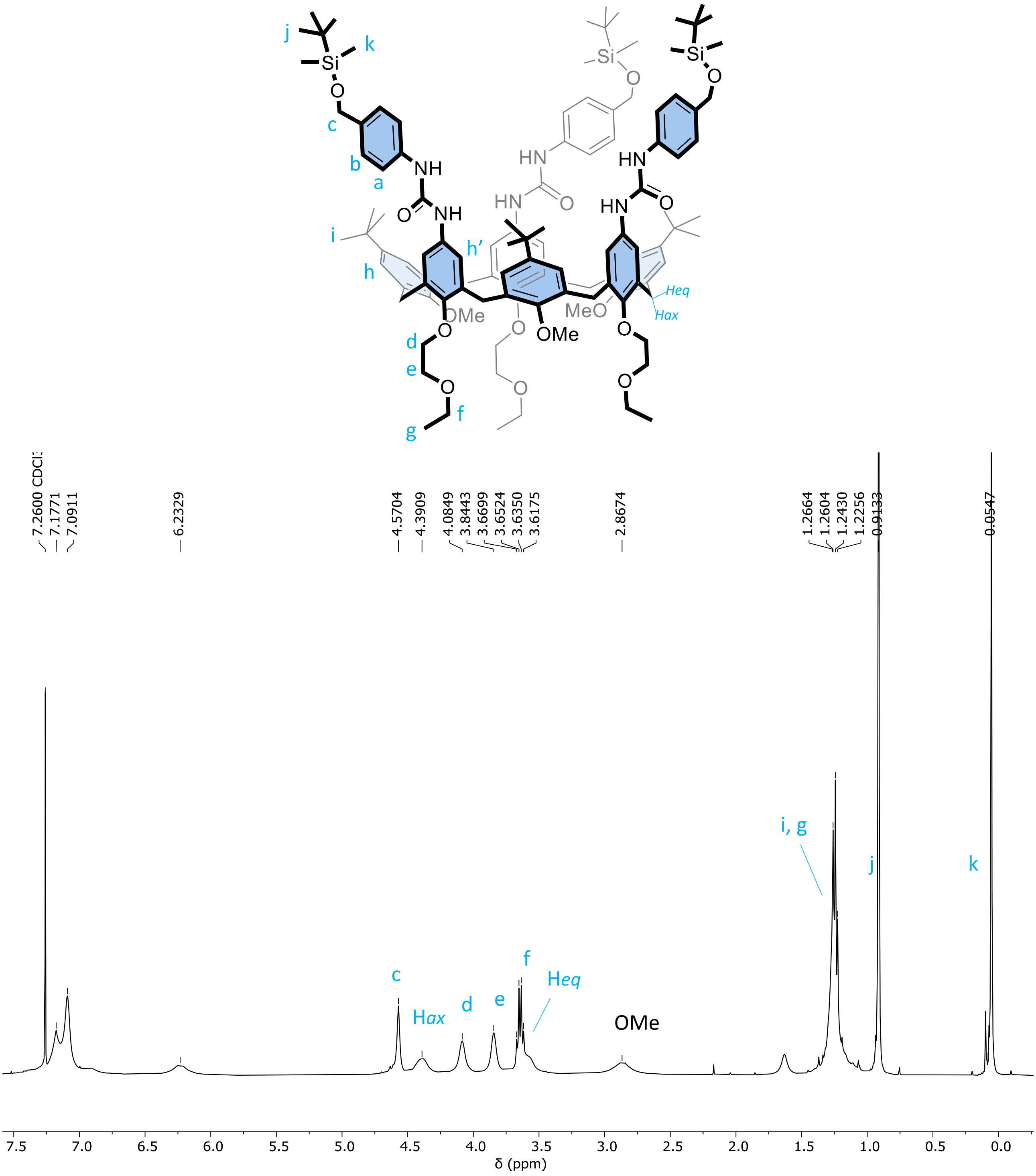

Figure S17: ^{1}H NMR spectrum (400 MHz, $CDCl_3$) of compound **TPU-OTBS**.

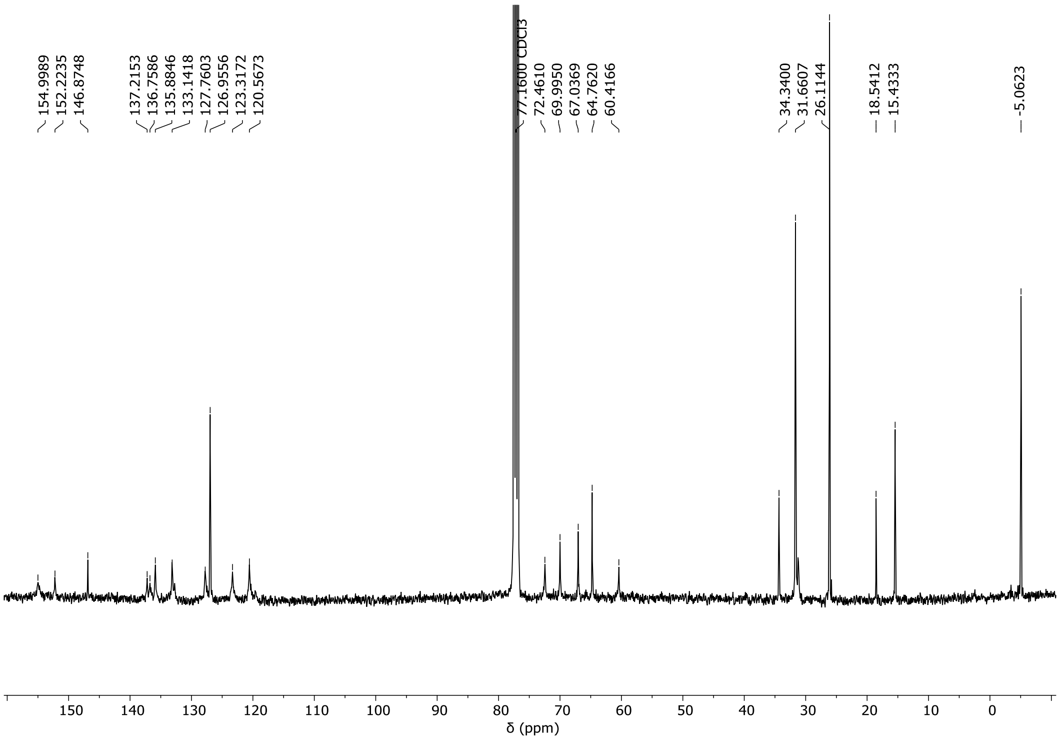

Figure S18: ^{13}C NMR spectrum (100 MHz, $CDCl_3$) of compound **TPU-OTBS**.

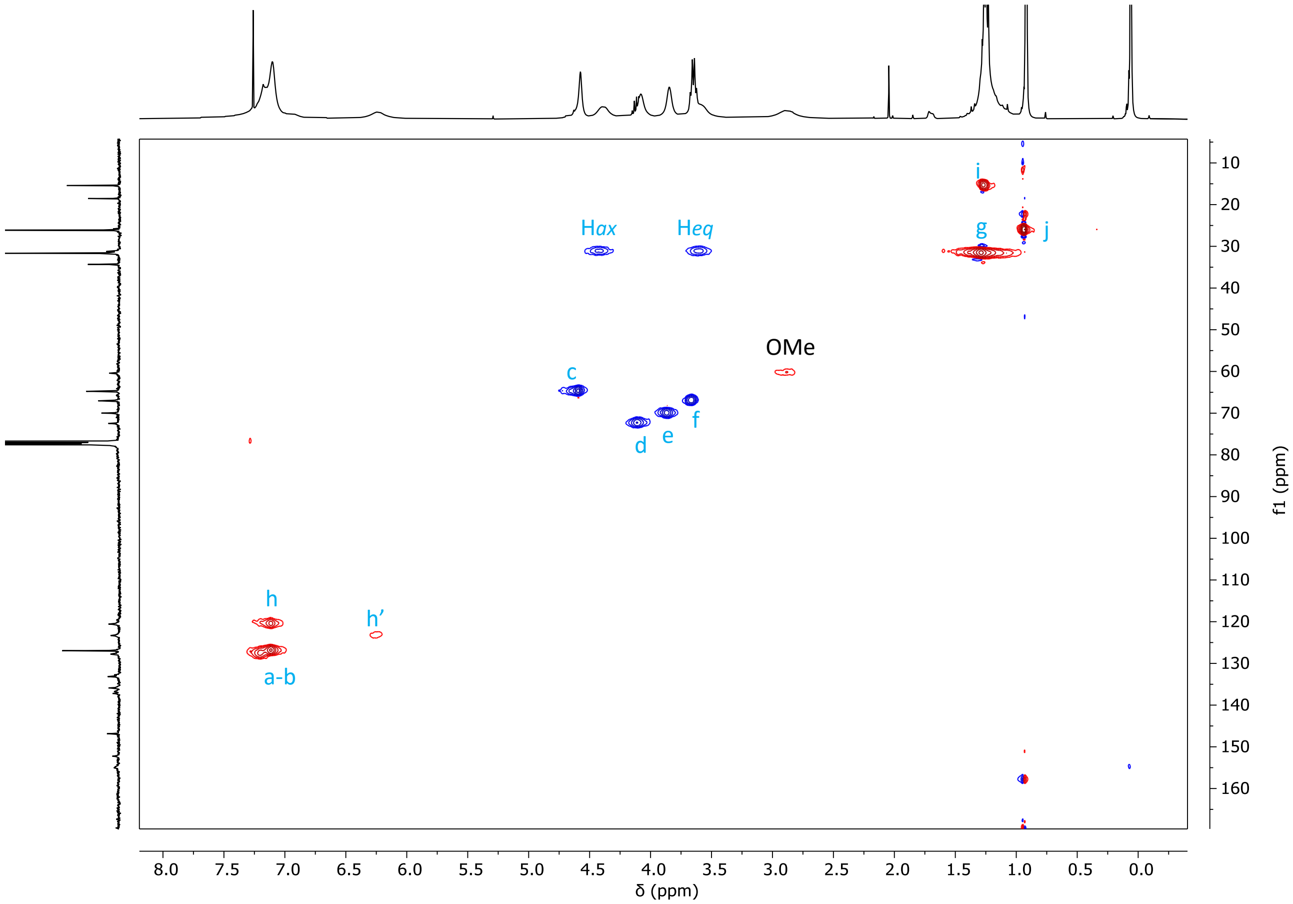

Figure S19: Edited HSQC 2D NMR spectrum (400 MHz, $CDCl_3$) of compound **TPU-OTBS**. Positive peaks (CH_3 and CH) are shown in red, while negative ones (CH_2) are in blue.

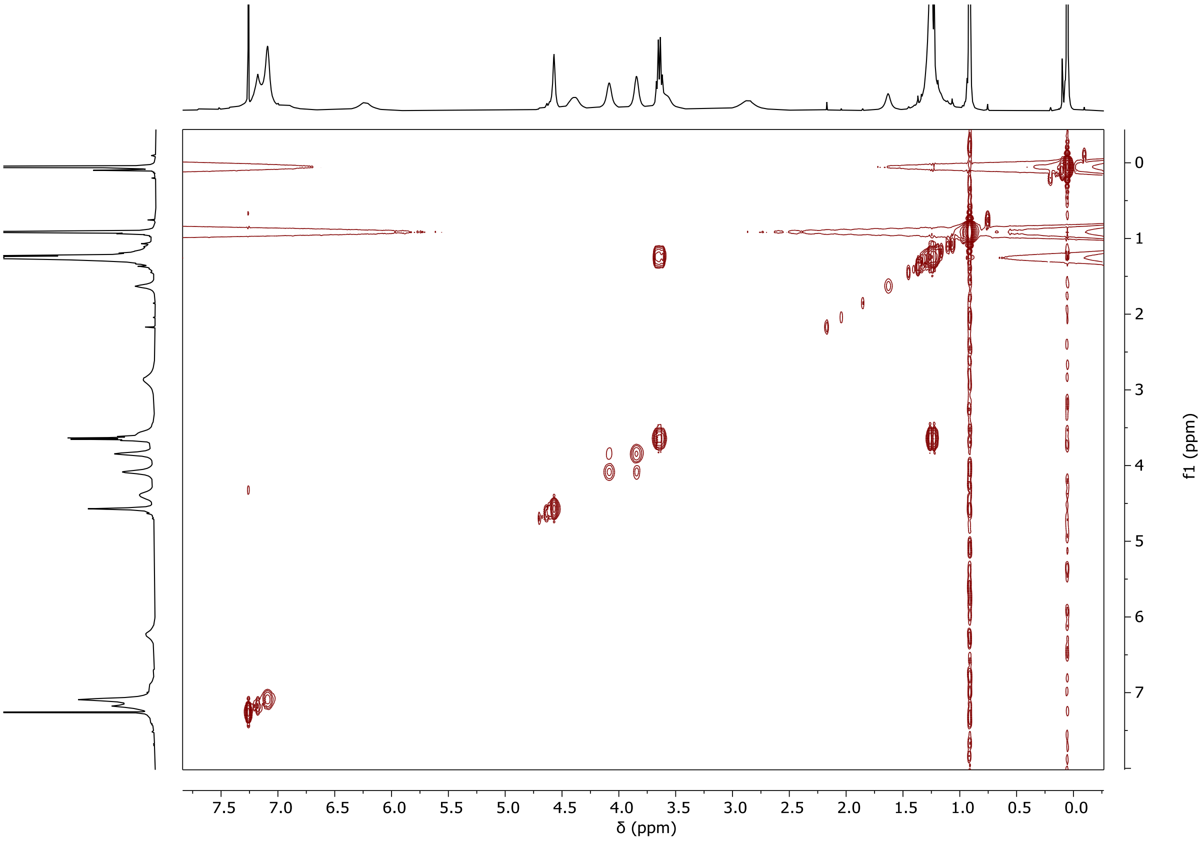

Figure S20: 2D magnitude g-COSY NMR spectrum (400 MHz, $CDCl_3$) of compound **TPU-OTBS**.

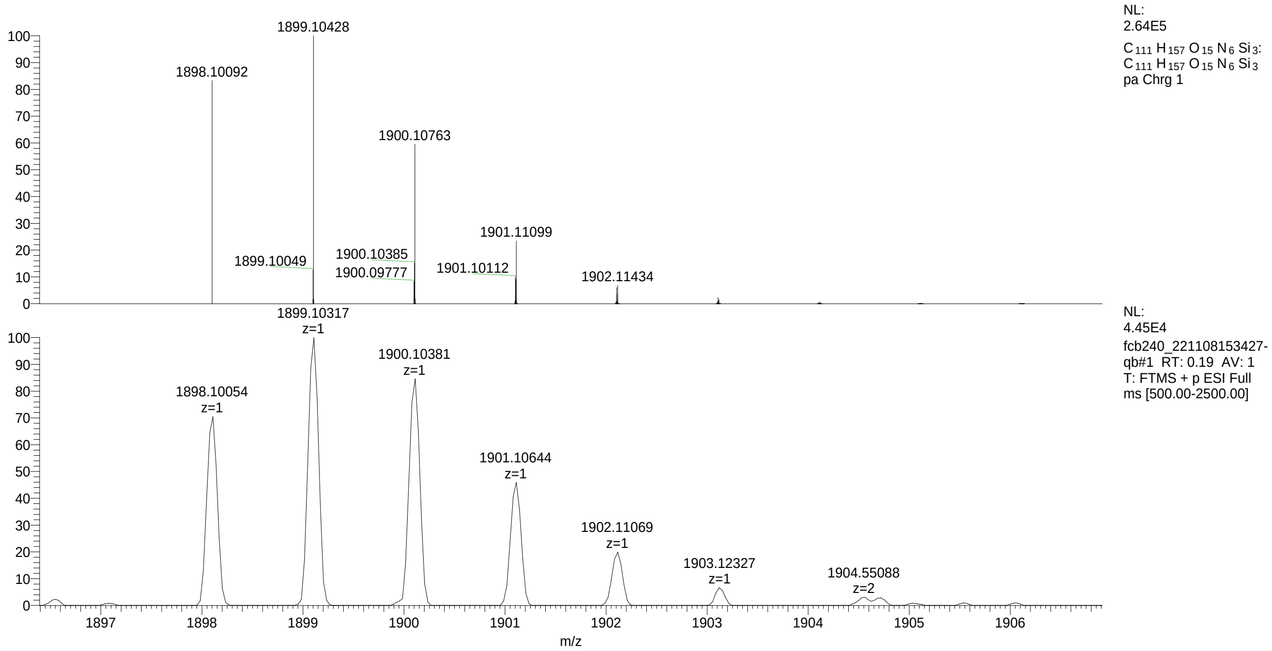

Figure S21: Inset of HR-MS (ESI, Orbitrap LQ) spectrum of compound **TPU-OTBS:** calculated (top) and experimental (down) isotopic distribution for the singly charged molecular ion.

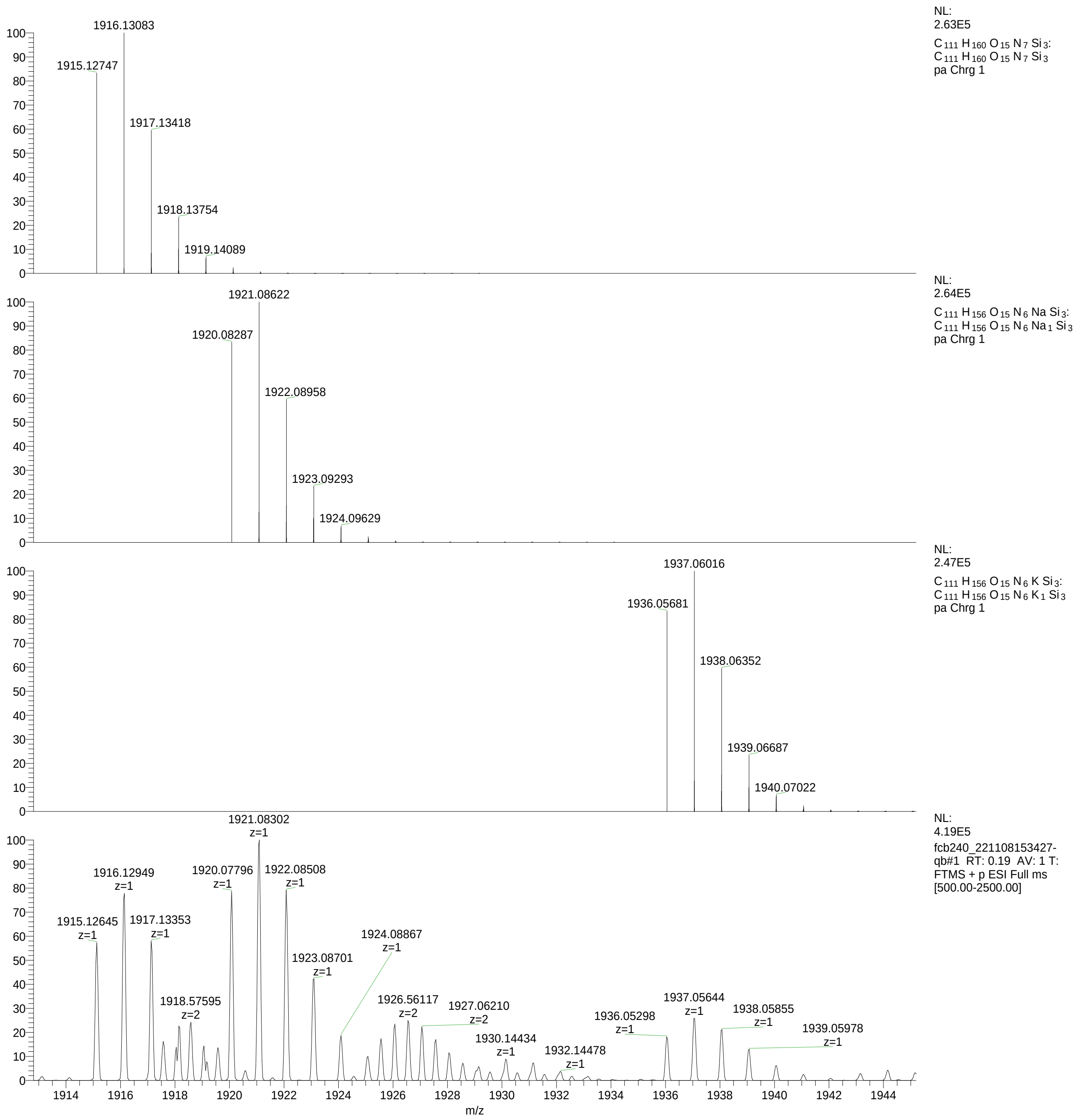

Figure S22: Inset of HR-MS (ESI, Orbitrap LQ) spectrum of compound **TPU-OTBS:** calculated (top) and experimental (down) isotopic distributions for singly charged molecular adducts with ammonium, sodium, and potassium ions, respectively.

Characterisation of *P*[(**TPU**)$_2$⊃**5**$_6$]

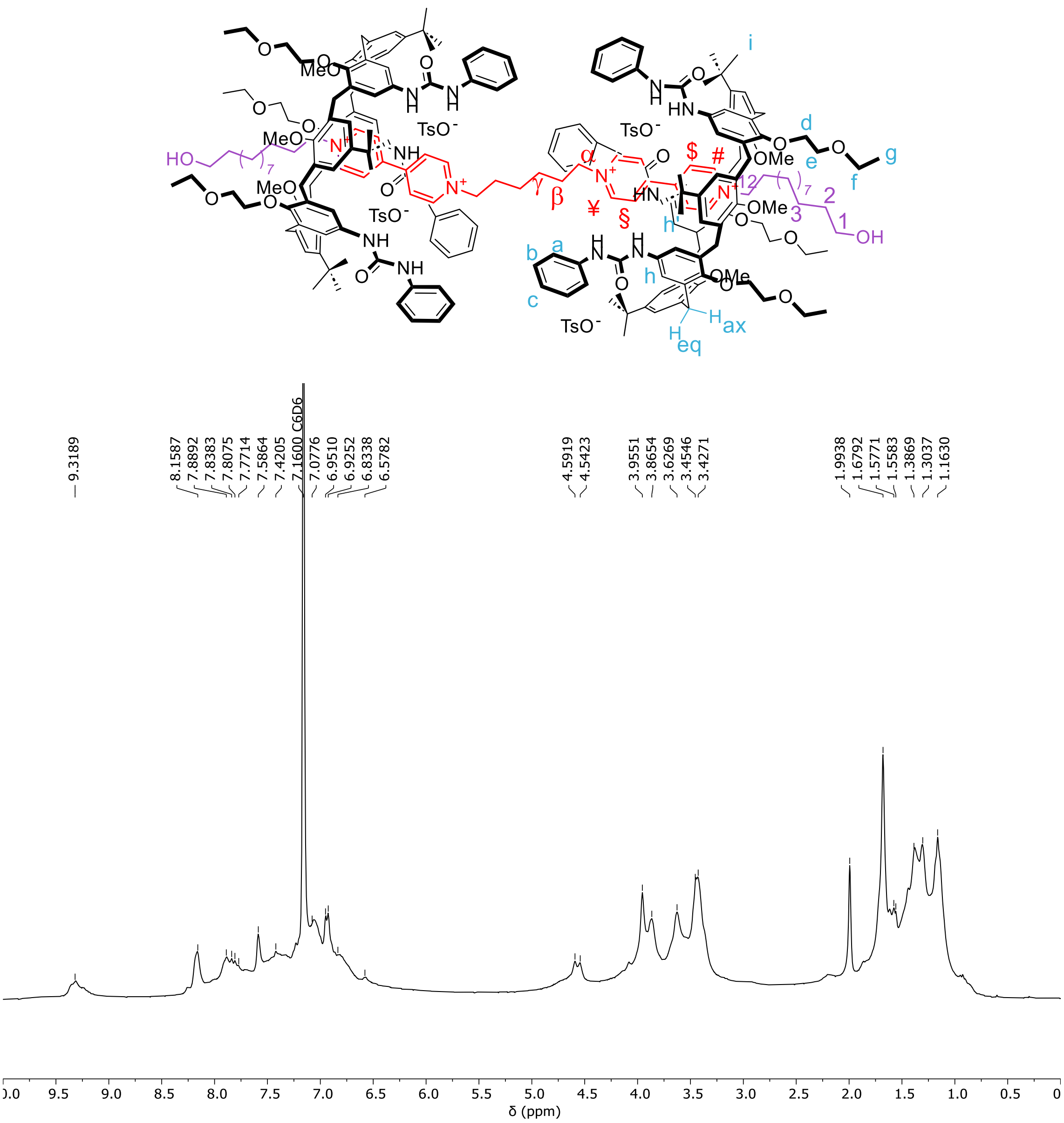

Figure S23: ^{1}H NMR spectrum (400 MHz, benzene-d$_6$) of [3]pseudorotaxane *P*[(**TPU**)$_2$⊃**5**$_6$].

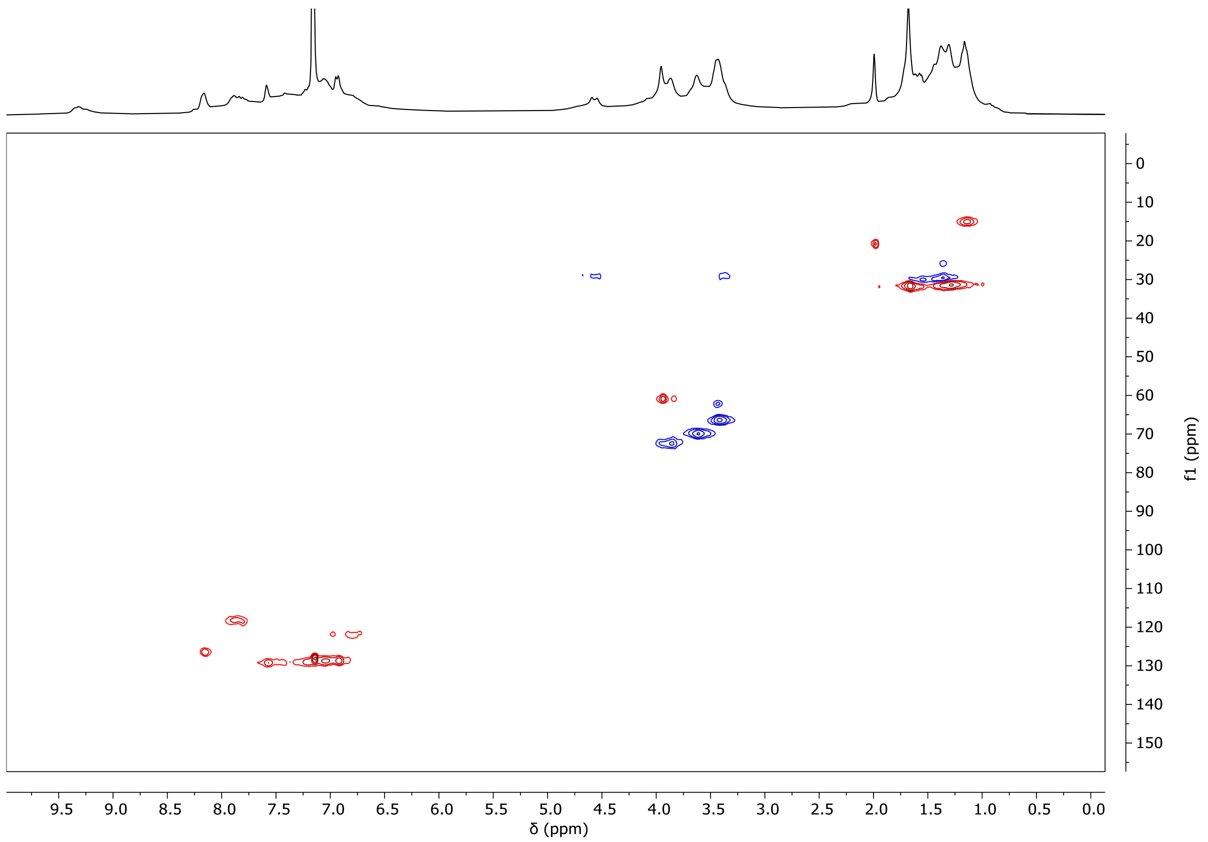

Figure S24: Edited HSQC 2D NMR spectrum (400 MHz, benzene-d$_6$) of [3]pseudorotaxane *P*[(**TPU**)$_2$⊃**5**$_6$]. Positive peaks (CH_3 and CH) are shown in red, while negative ones (CH_2) are in blue.

Characterisation of *P*[(**TPU**)$_2$⊃**5**$_{12}$]

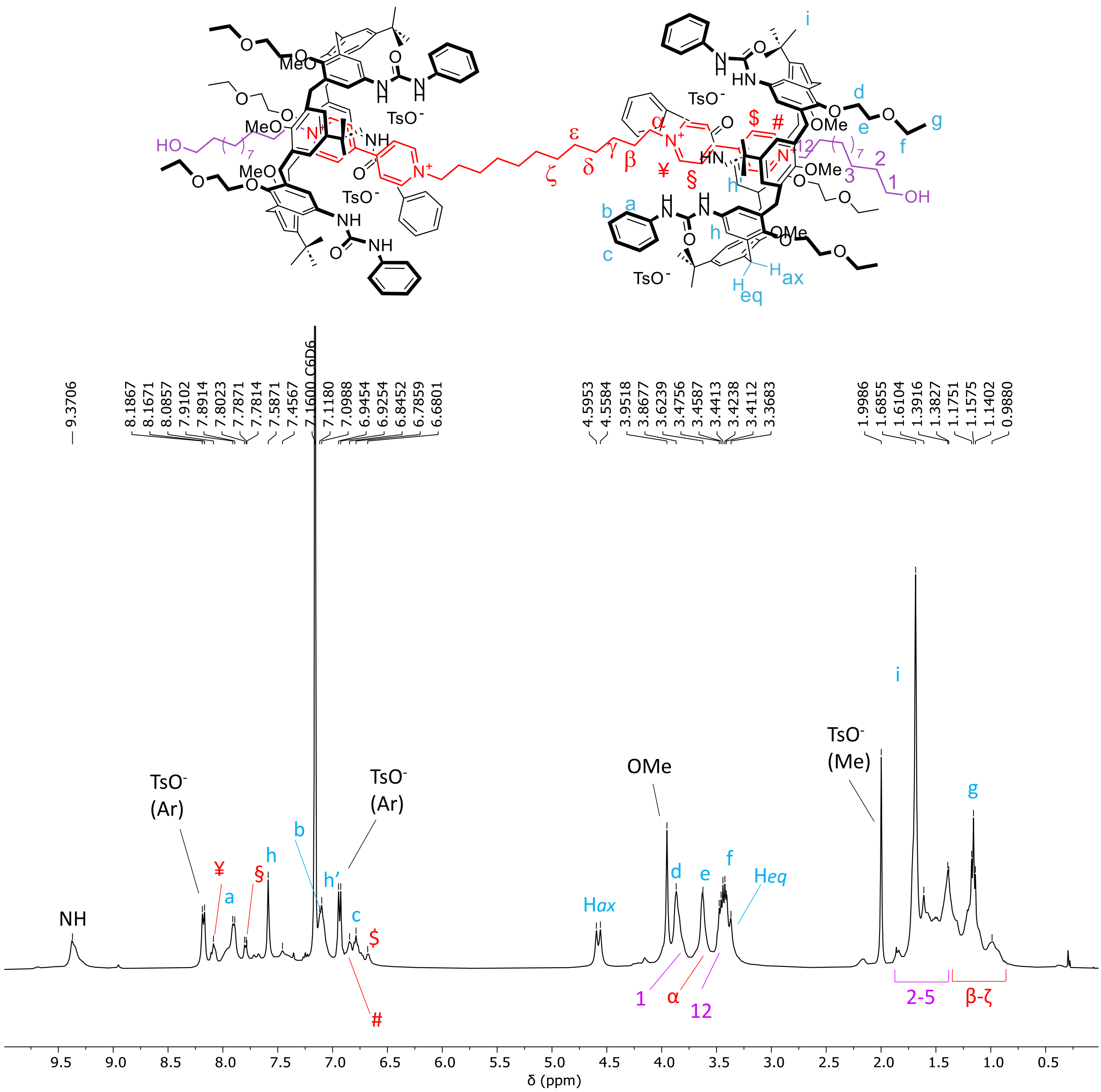

Figure S25: ^{1}H NMR spectrum (400 MHz, benzene-d$_6$) of [3]pseudorotaxane *P*[(**TPU**)$_2$⊃**5**$_{12}$].

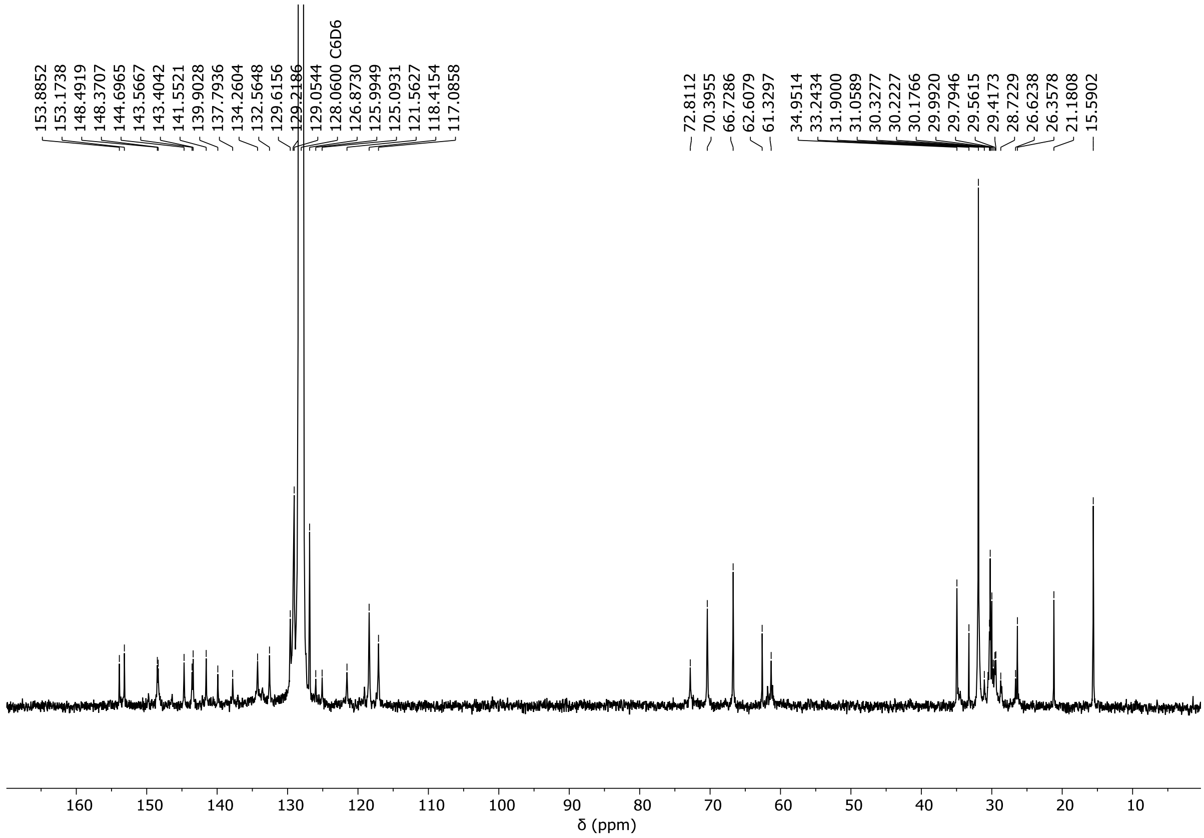

Figure S26: ^{13}C NMR spectrum (100 MHz, benzene-d_6) of [3]pseudorotaxane *P*[(**TPU**)$_2$⊃**5**$_{12}$].

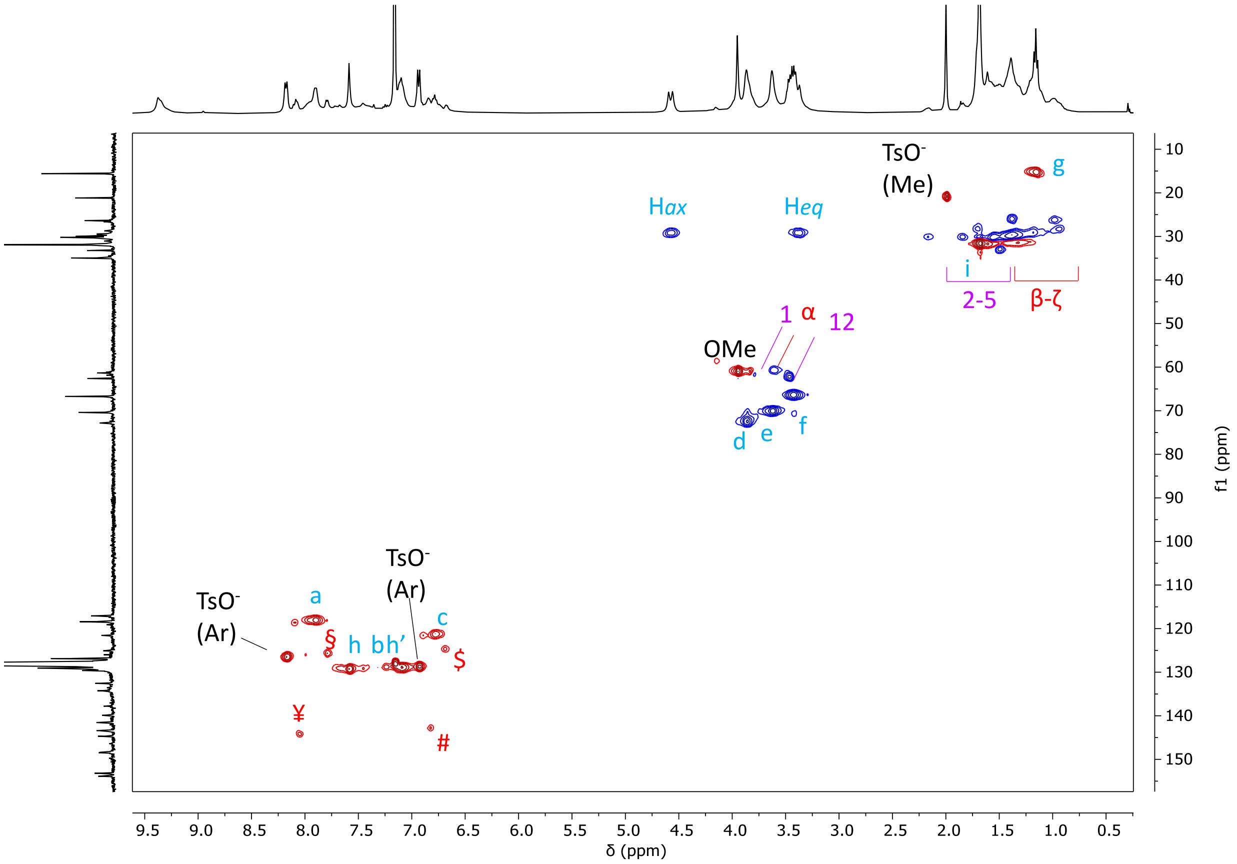

Figure S27: Edited HSQC NMR spectrum (400 MHz, benzene-d_6) of [3]pseudorotaxane *P*[(**TPU**)$_2$⊃**5**$_{12}$]. Positive peaks (CH_3 and CH) are shown in red, while negative ones (CH_2) are in blue.

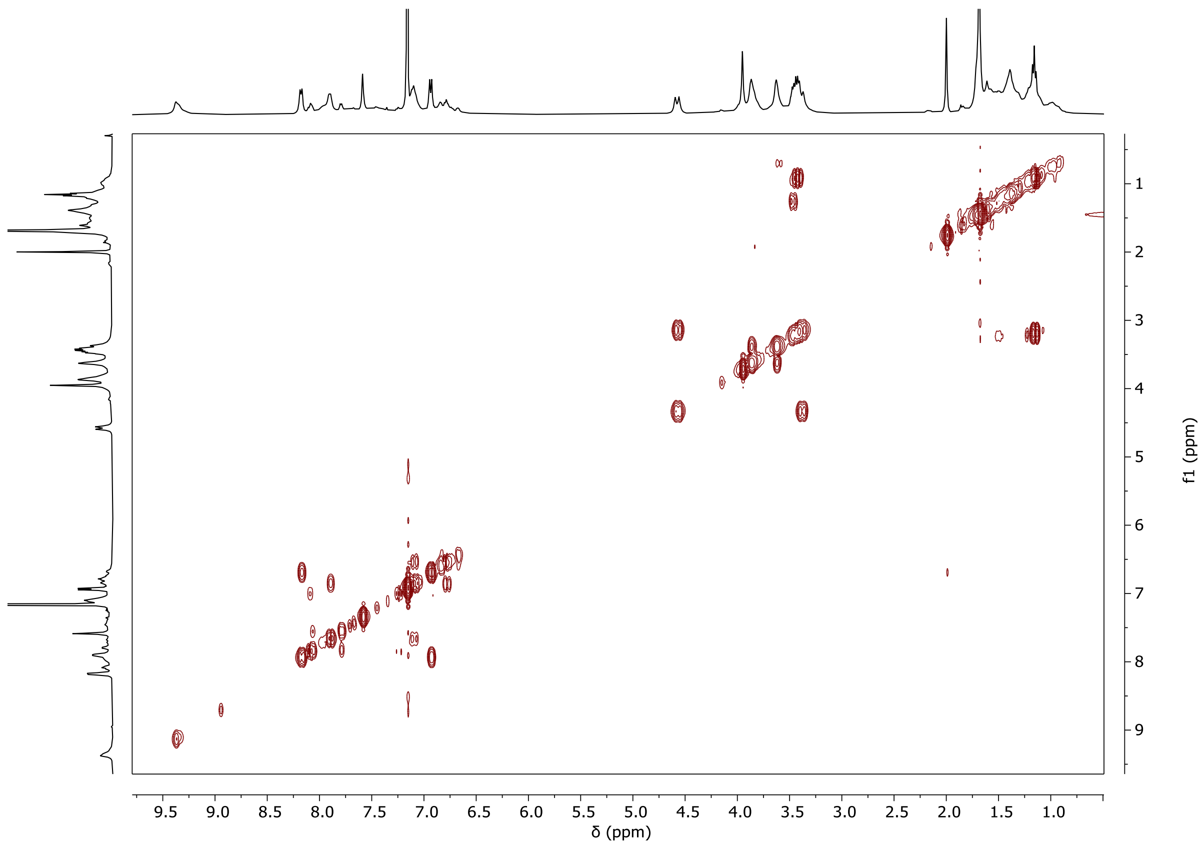

Figure S28: 2D magnitude g-COSY NMR spectrum (400 MHz, benzene-d6) of [3]pseudorotaxane *P*[(**TPU**)2⊃**5**12].

Characterisation of *R*[(**TPU**)$_2$⊃ **6**$_{12}$]

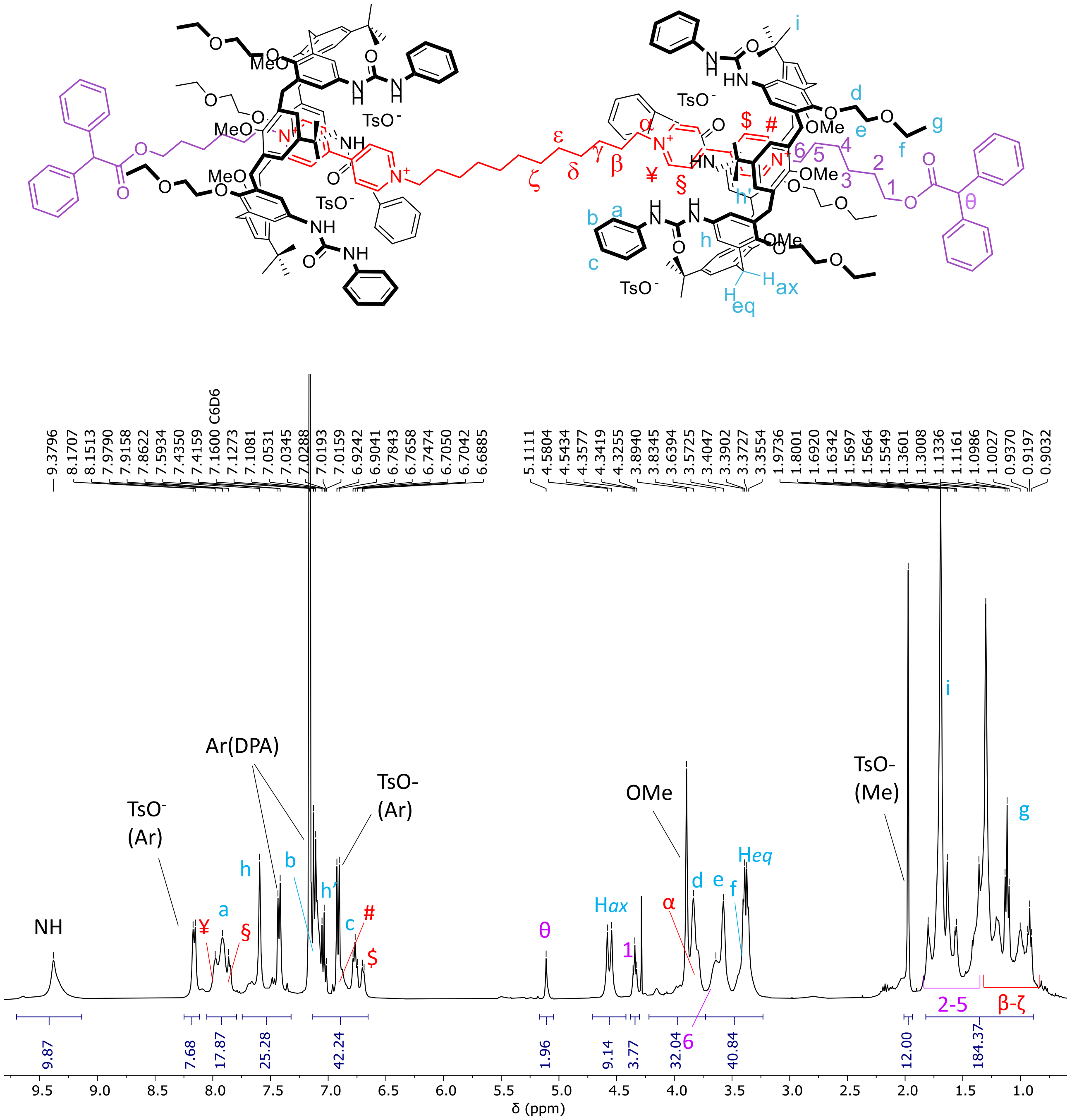

Figure S29: ^{1}H NMR spectrum (400 MHz, benzene-d$_6$) of [3]rotaxane *R*[(**TPU**)$_2$⊃**6**$_{12}$].

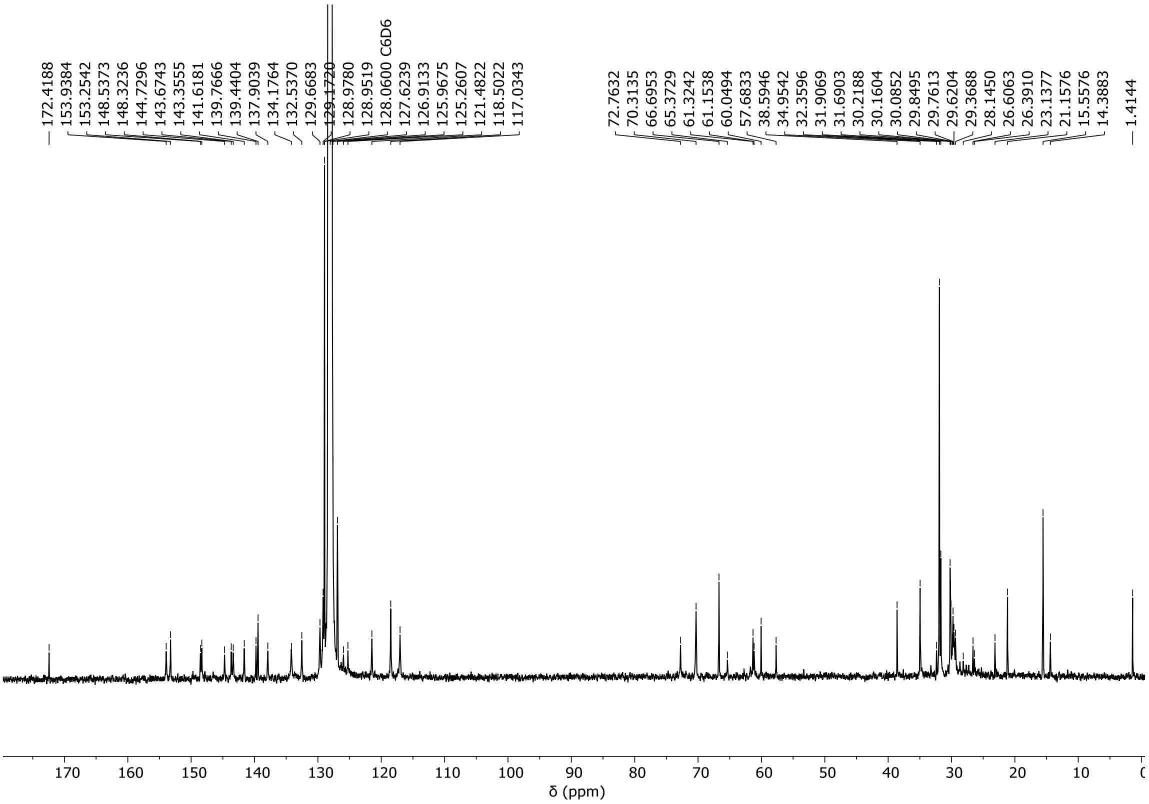

Figure S30: ^{13}C NMR spectrum (100 MHz, benzene-d$_6$) of [3]rotaxane *R*[(**TPU**)$_2$⊃**6**$_{12}$].

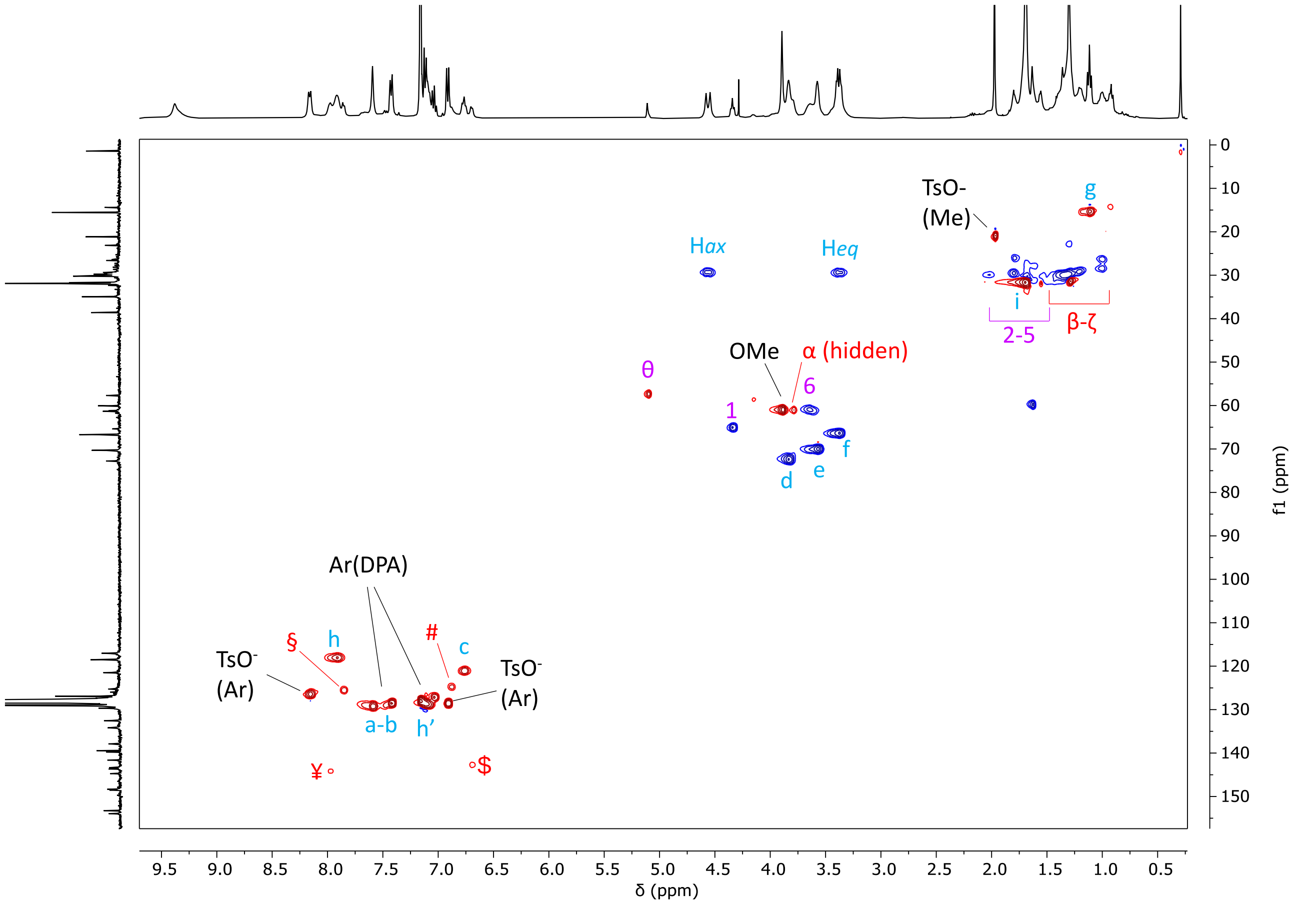

Figure S31: Edited HSQC NMR spectrum (400 MHz, benzene-d$_6$) of [3]rotaxane *R*[(**TPU**)$_2$⊃**6**$_{12}$]. Positive peaks (CH_3 and CH) are shown in red, while negative ones (CH_2) are in blue.

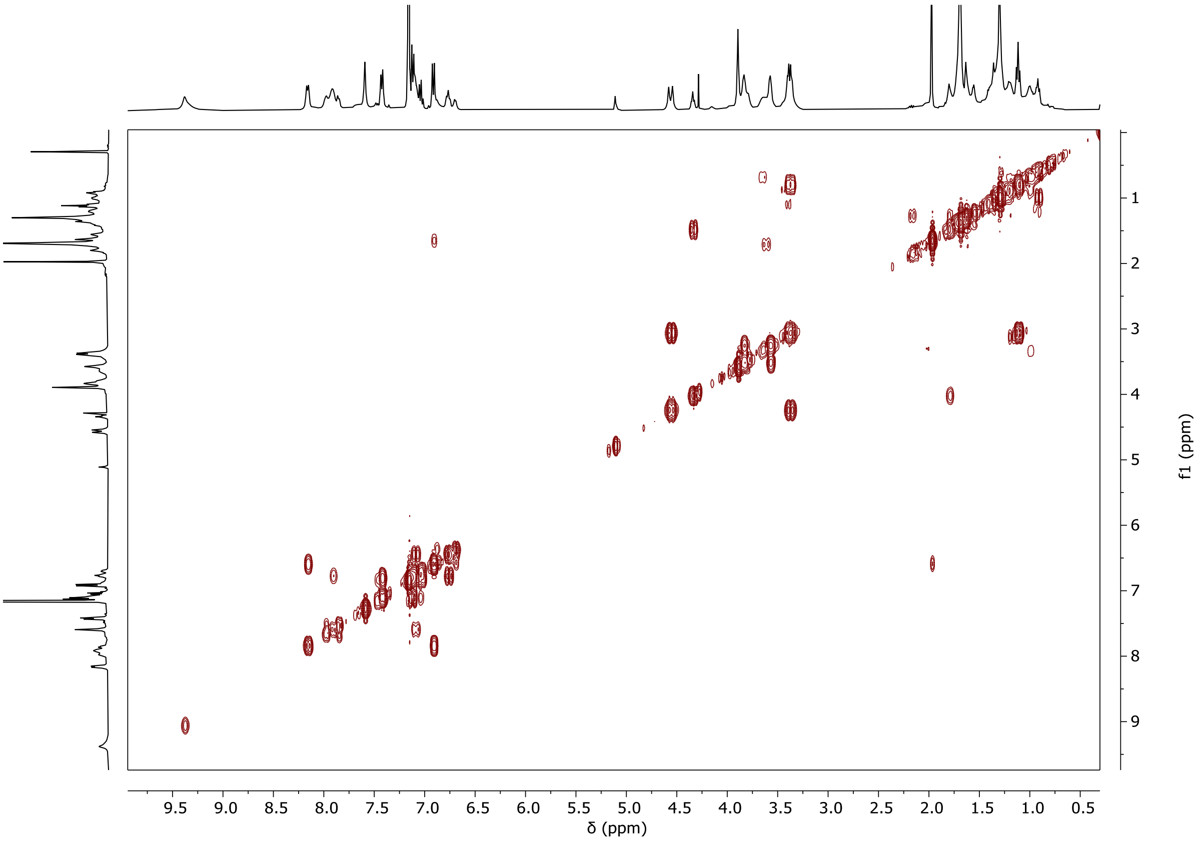

Figure S32: 2D magnitude g-COSY NMR spectrum (400 MHz, benzene-d$_6$, 25 °C) of [3]rotaxane *R*[(**TPU**)$_2$⊃**6**$_{12}$].

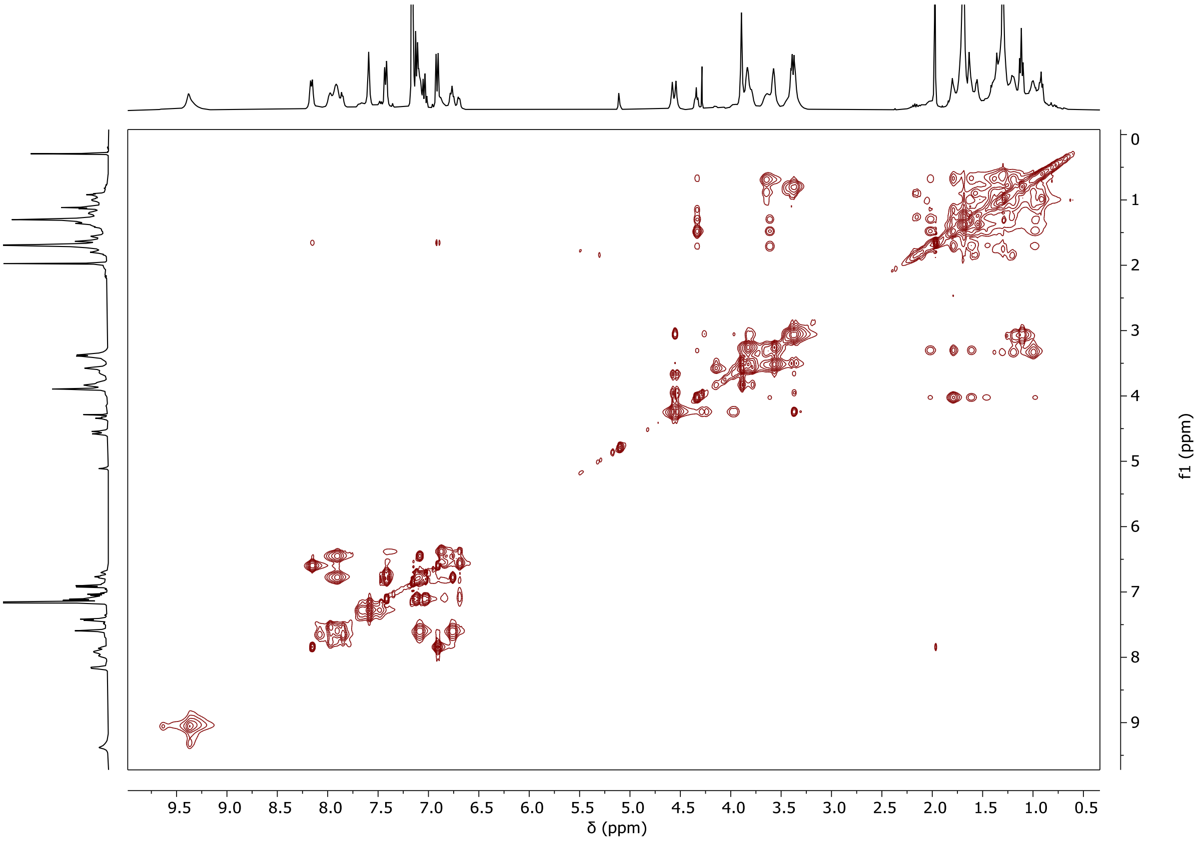

Figure S33: 2D TOCSY NMR spectrum (400 MHz, benzene-d$_6$, MT = 0.06 s) of [3]rotaxane *R*[(**TPU**)$_2$⊃**6**$_{12}$].

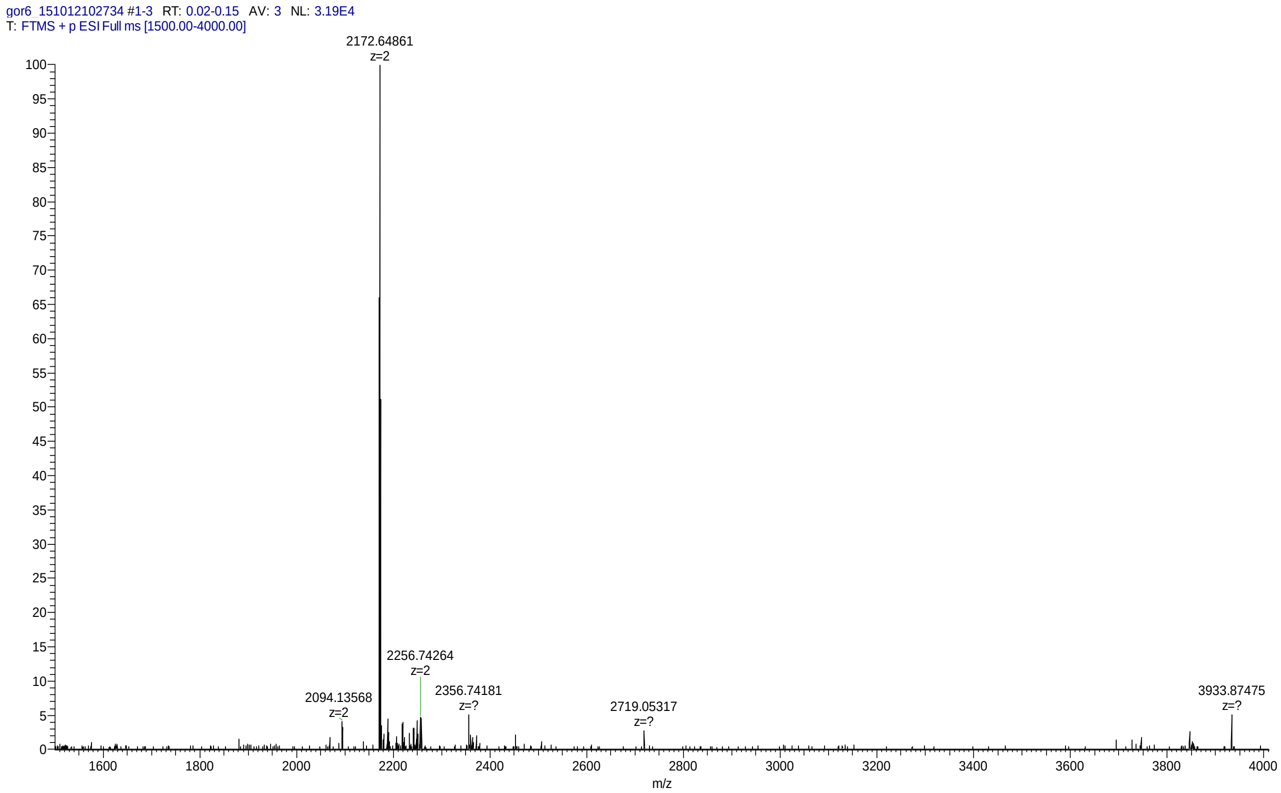

Figure S34: HR-MS (ESI, Orbitrap LQ) spectrum of [3]rotaxane R[(TPU)$_2$⊃**6**$_{12}$].

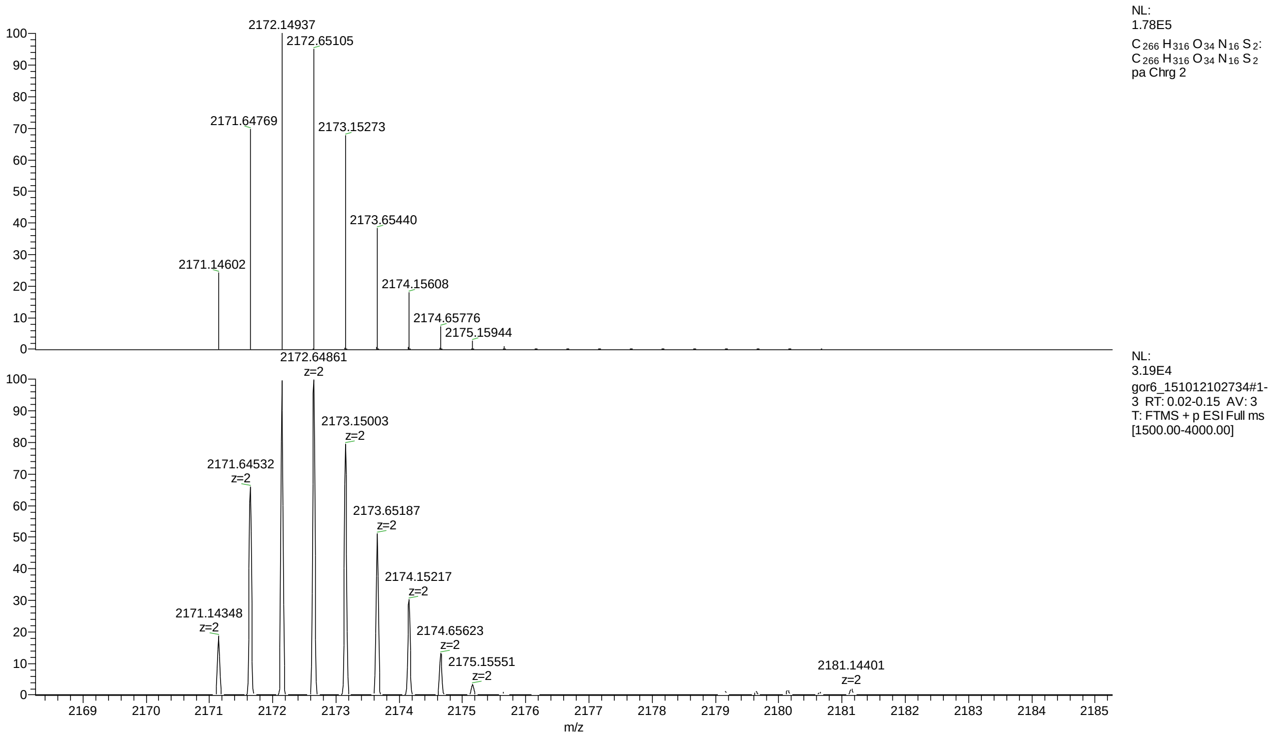

Figure S35: Inset of HR-MS (ESI, Orbitrap LQ) spectrum of compound *R*[(**TPU**)$_2$⊃**6**$_{12}$]: calculated (top) and experimental (down) isotopic distribution for the singly charged molecular ion.

Characterisation of *R*[(**TPU**)$_2$⊃ **7**$_{12}$]

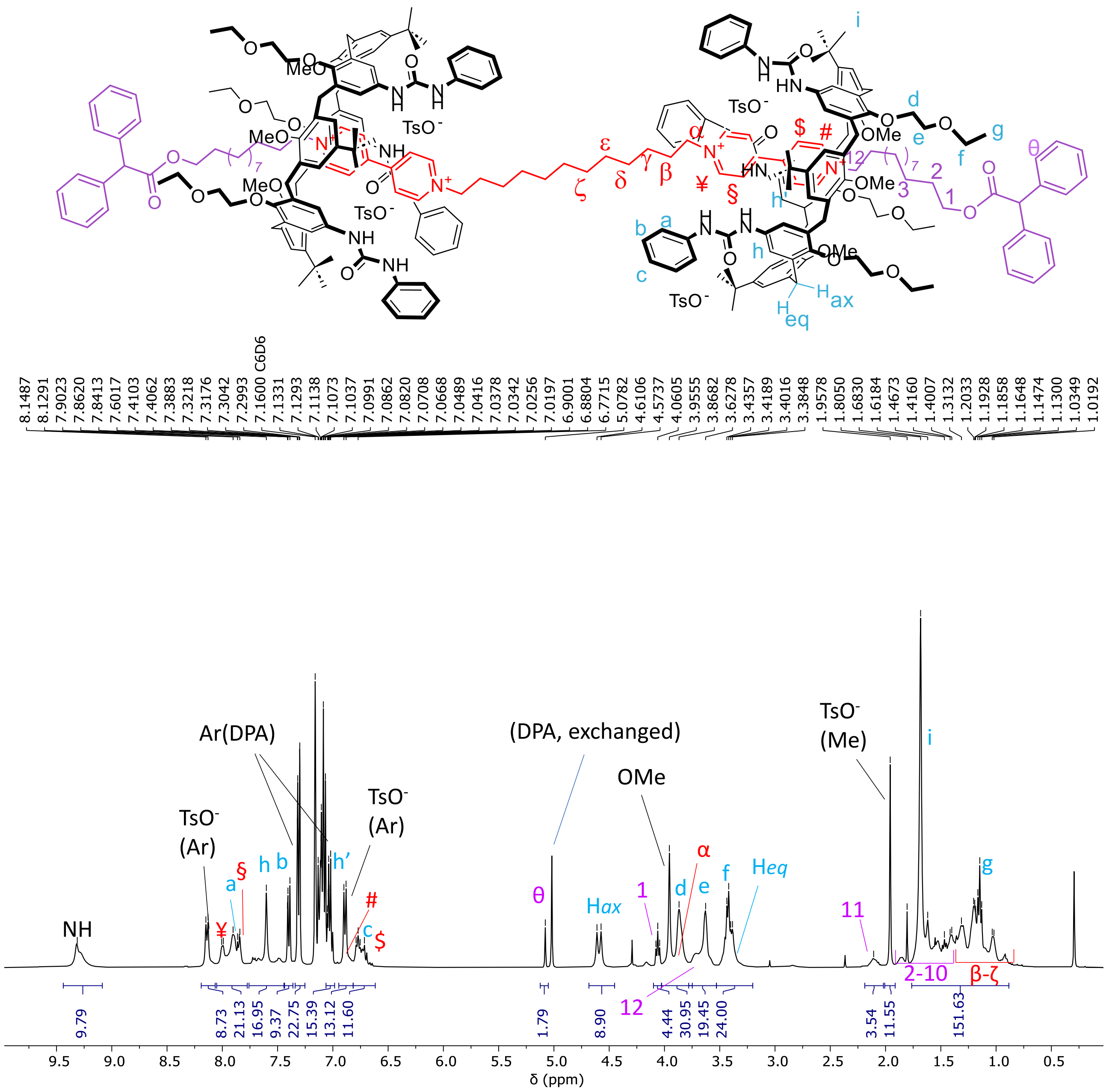

Figure S36: ^{1}H NMR spectrum (400 MHz, benzene-d$_6$) of [3]rotaxane *R*[(**TPU**)$_2$⊃**7**$_{12}$]. The resonance with the DPA label at 5.02 ppm is associated with the signal of the diphenylacetate methine proton that exchanged the tosylates upon axle stoppering.

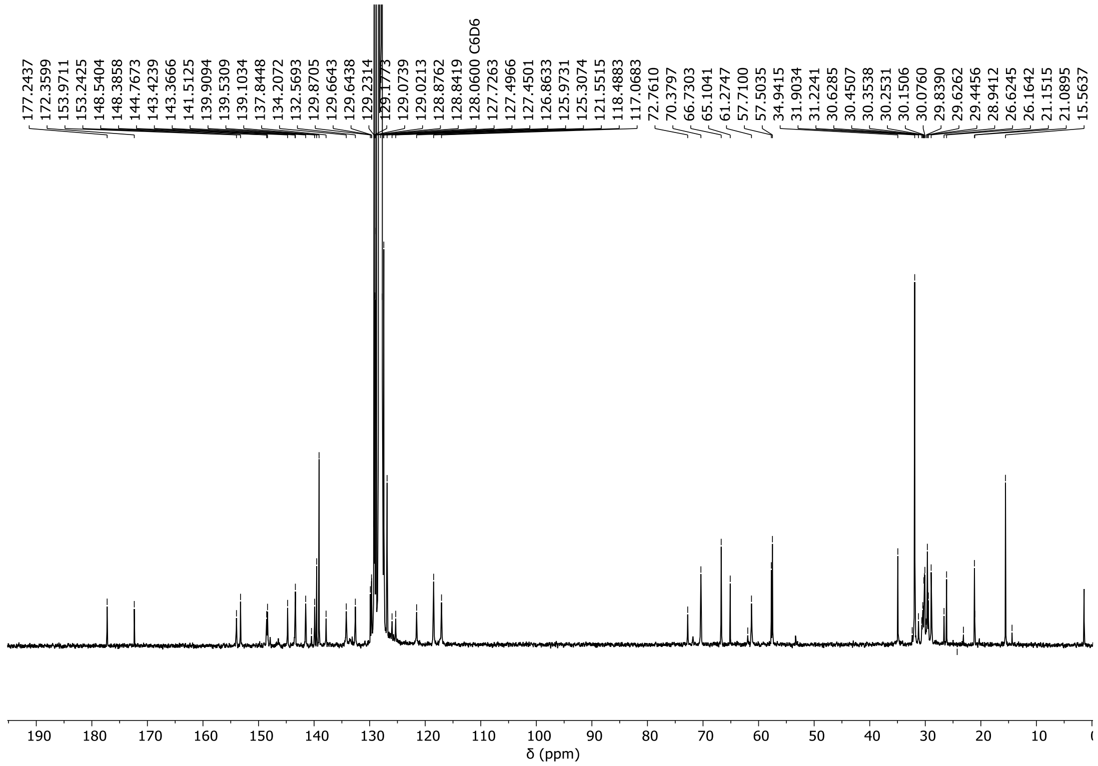

Figure S37: ^{13}C NMR spectrum (100 MHz, benzene-d_6) of [3]rotaxane *R*[(**TPU**)$_2$⊃**7**$_{12}$].

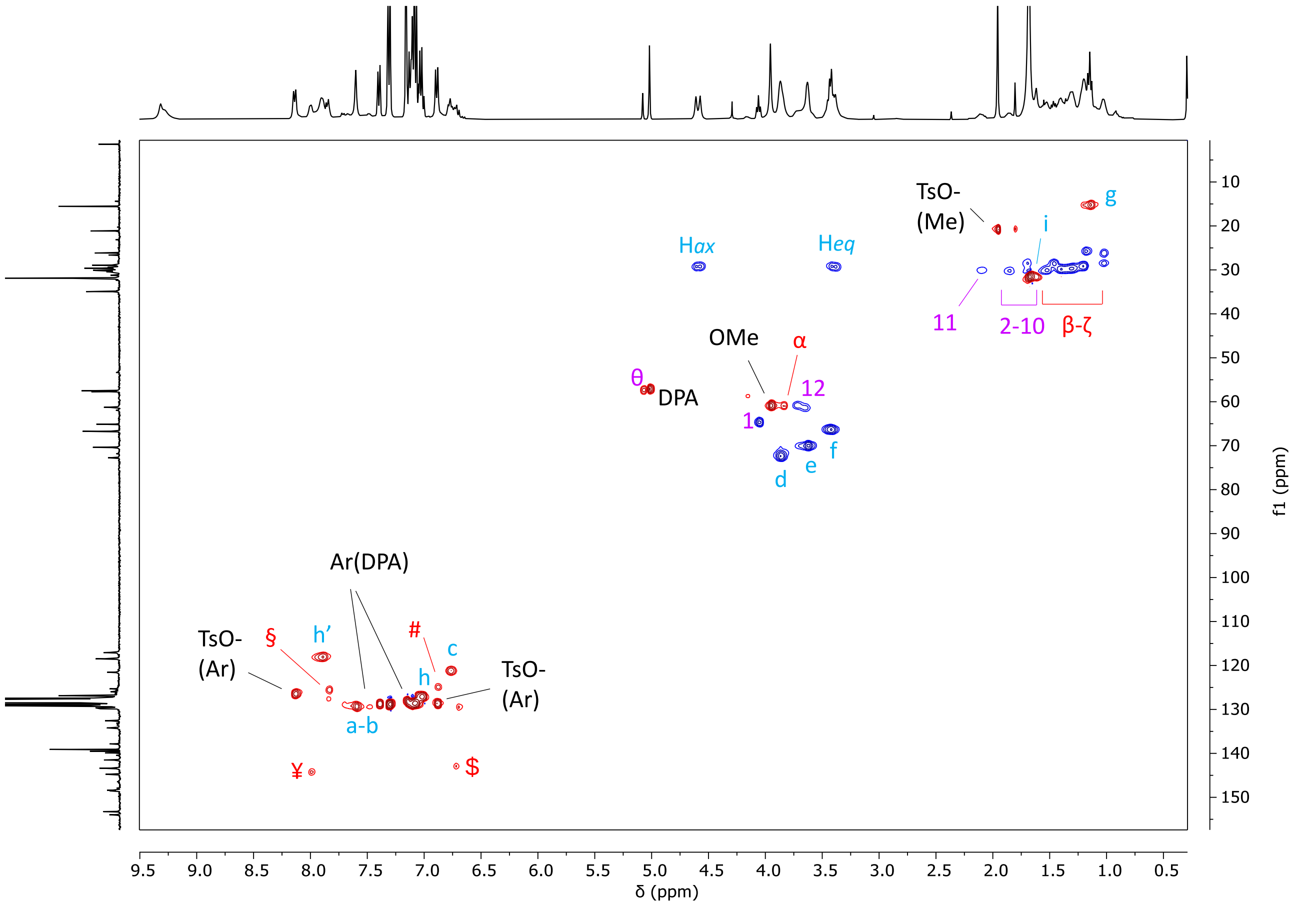

Figure S38: Edited HSQC NMR spectrum (400 MHz, benzene-d_6) of [3]rotaxane *R*[(**TPU**)$_2$⊃**7**$_{12}$]. Positive peaks (CH_3 and CH) are shown in red, while negative ones (CH_2) are in blue.

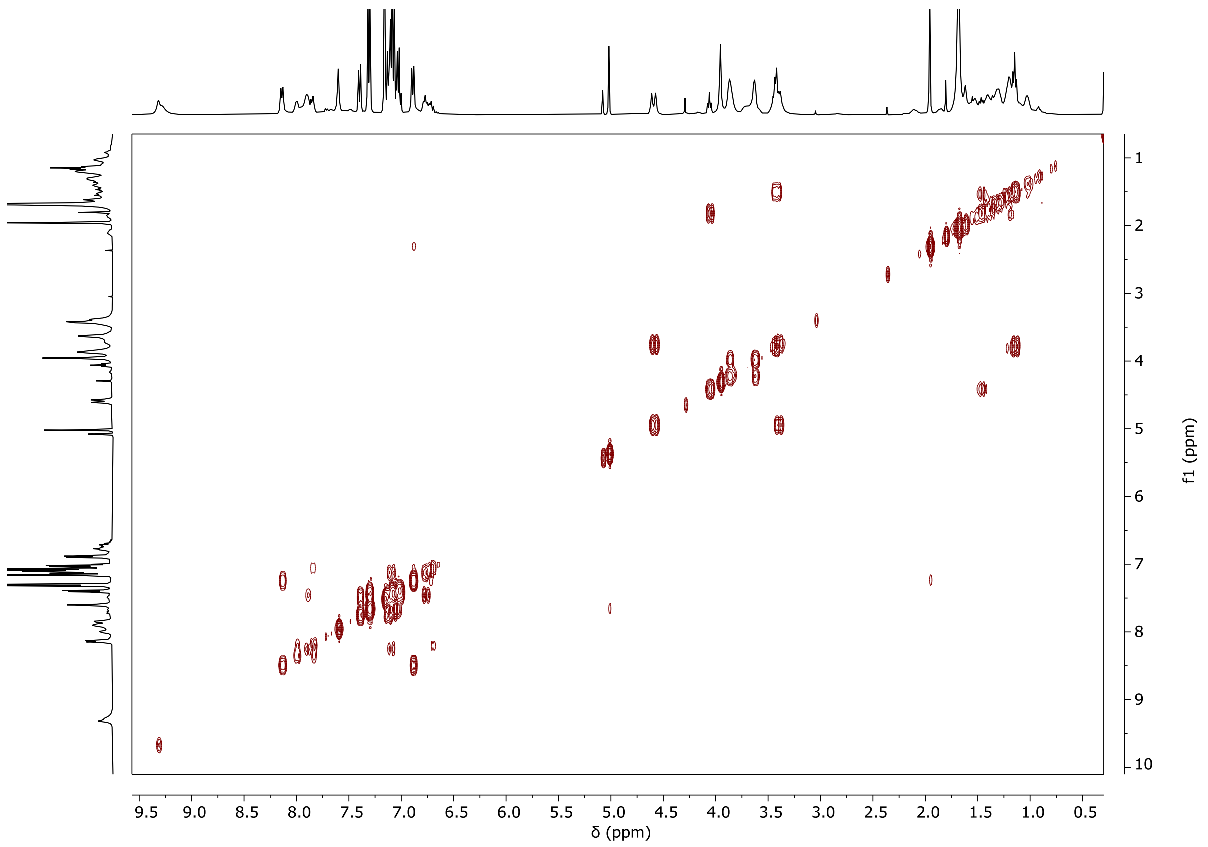

Figure S39: 2D magnitude g-COSY NMR spectrum of [3]rotaxane *R*[(**TPU**)$_2$⊃**7**$_{12}$] (400 MHz, benzene-d$_6$).

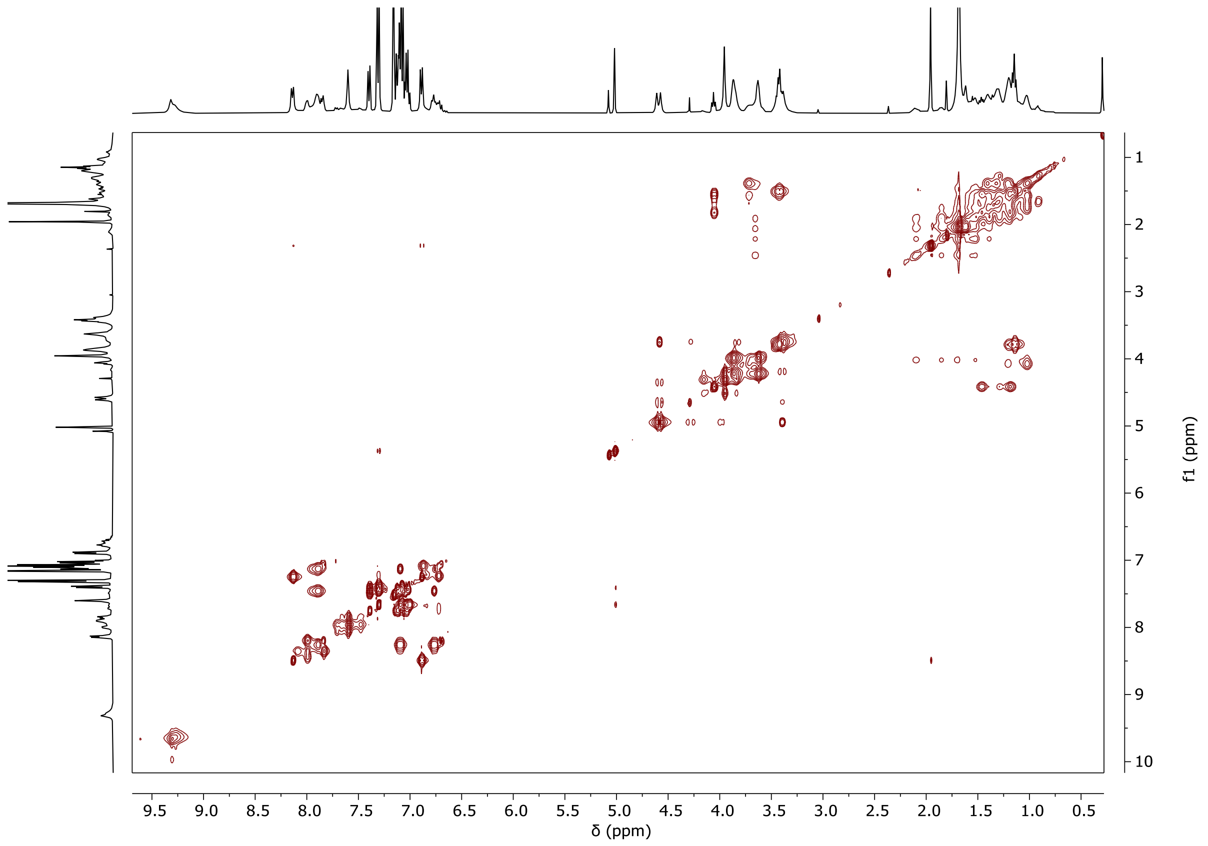

Figure S40: 2D TOCSY NMR spectrum (400 MHz, benzene-d$_6$, MT = 0.06 s) of [3]rotaxane *R*[(**TPU**)$_2$⊃**7**$_{12}$].

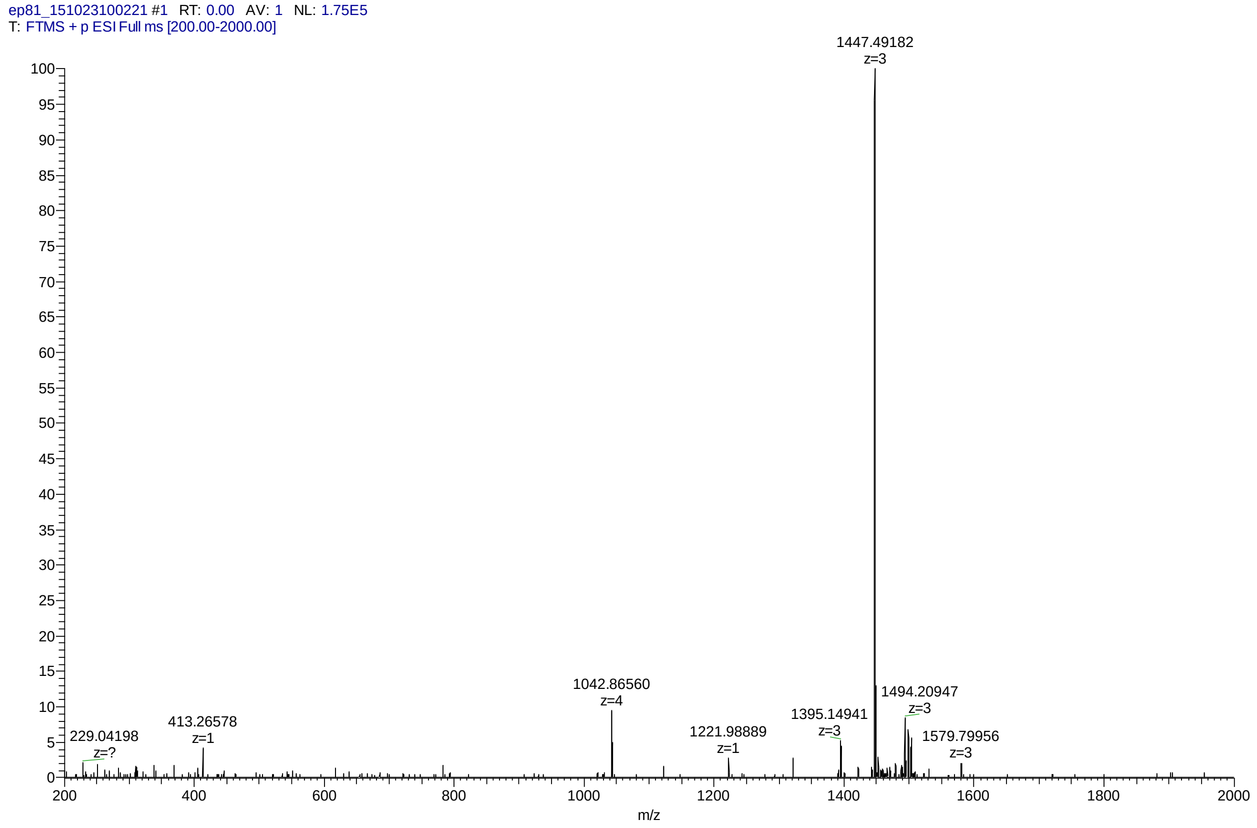

Figure S41: HR-MS (ESI, Orbitrap LQ) spectrum of [*3*]rotaxane R[(TPU)$_2$⊃**7**$_{12}$] showing the triply charged molecular ion.

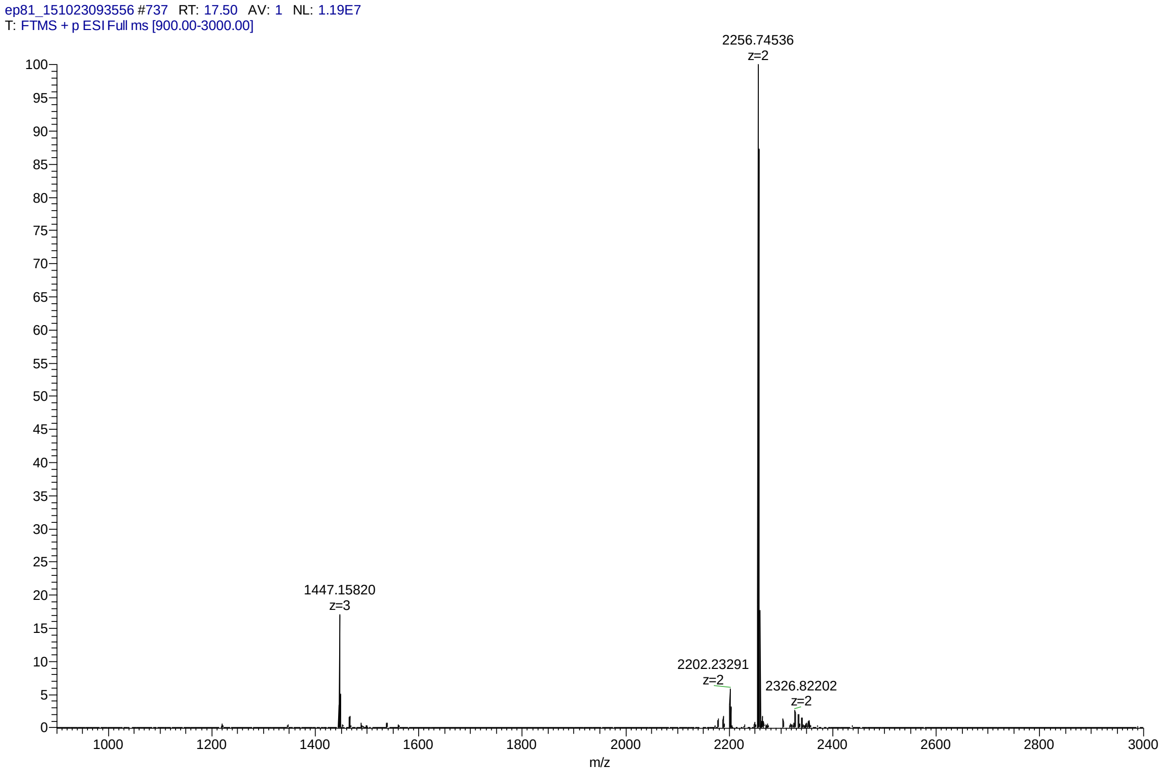

Figure S42: HR-MS (ESI, Orbitrap LQ) spectrum of [3]rotaxane *R*[(**TPU**)$_2$⊃**7**$_{12}$] showing the doubly charged molecular ion.

Characterisation of R[(**TPU**)$_2$⊃ **8**$_{12}$]

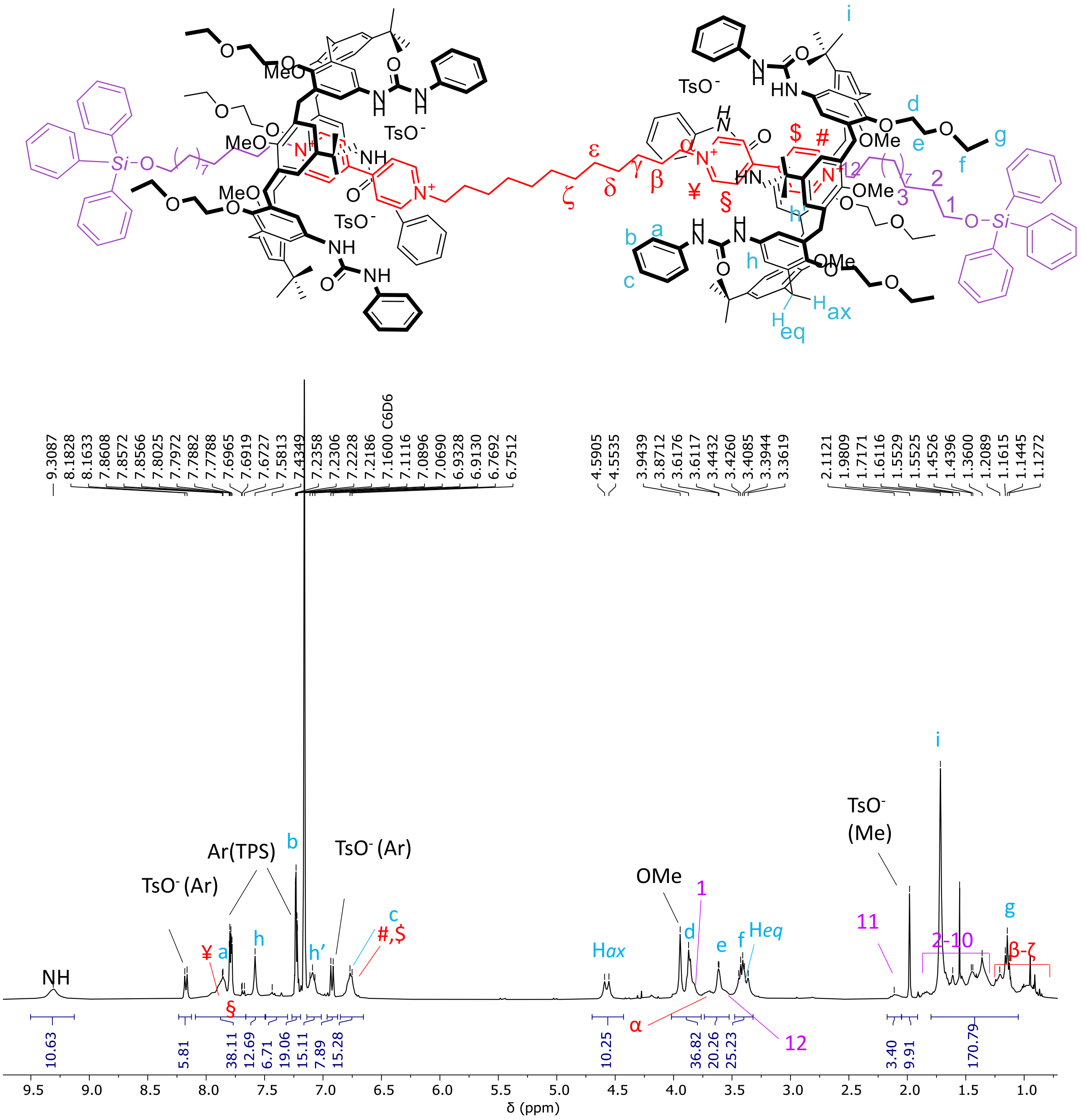

Figure S43: ^{1}H NMR spectrum (400 MHz, benzene-d$_6$) of [3]rotaxane R[(**TPU**)$_2$⊃**8**$_{12}$].

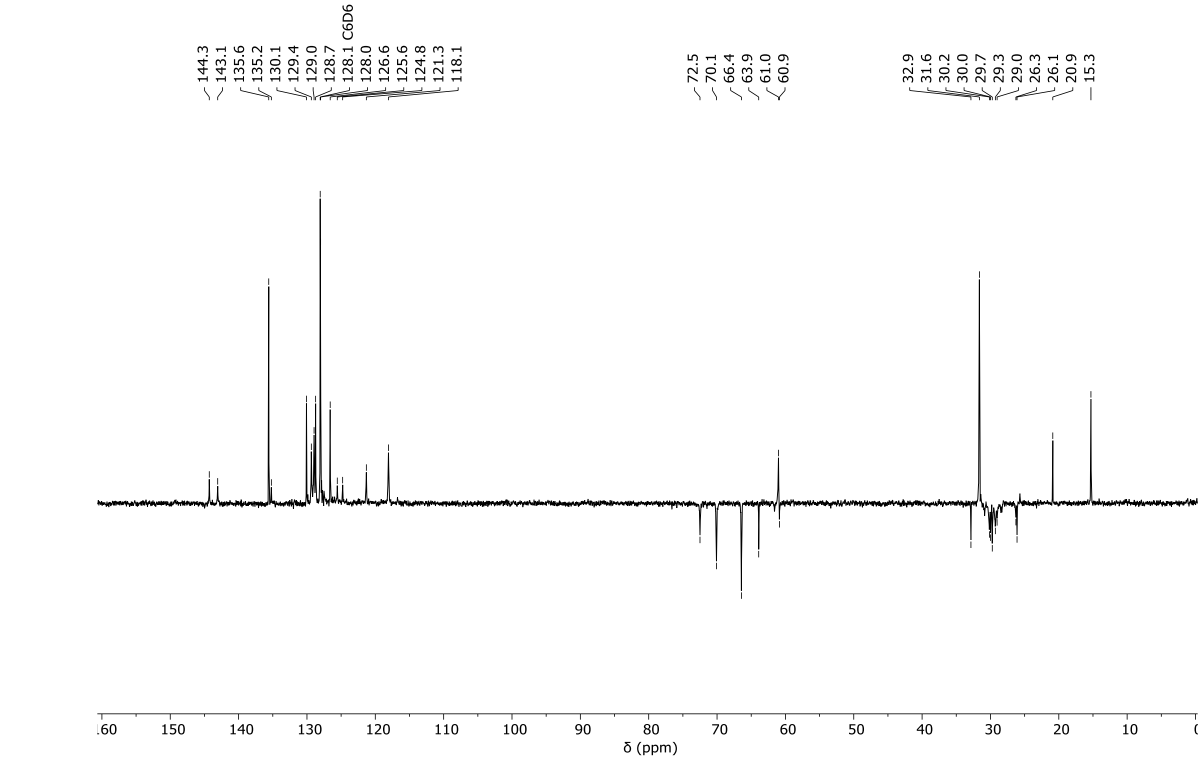

Figure S44: ^{13}C-APT NMR spectrum (100 MHz, benzene-d$_6$) of [3]rotaxane *R*[(**TPU**)$_2$⊃**8**$_{12}$].

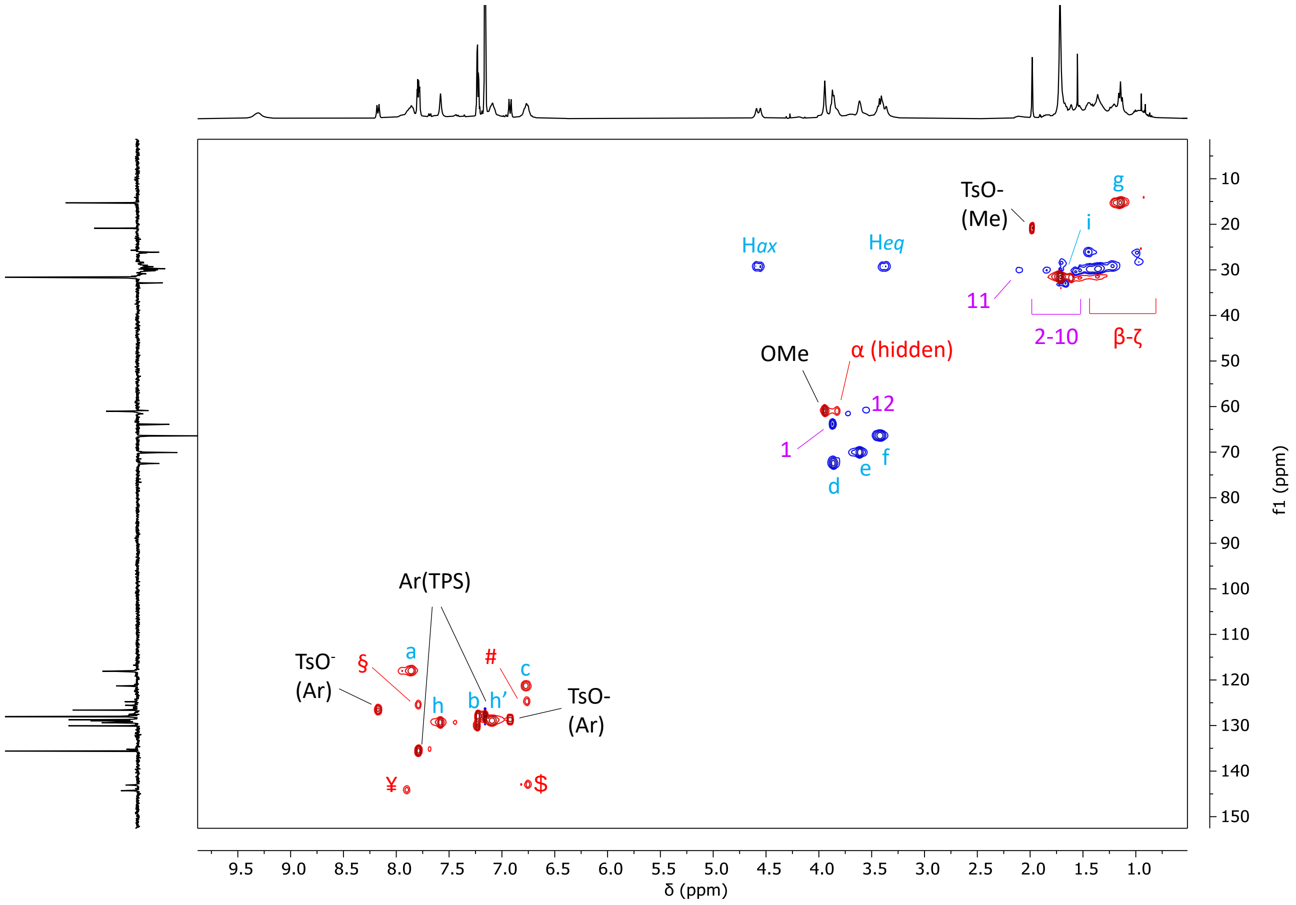

Figure S45: Edited HSQC NMR spectrum (400 MHz, benzene-d$_6$) of [3]rotaxane *R*[(**TPU**)$_2$⊃**8**$_{12}$]. Positive peaks (CH_3 and CH) are shown in red, while negative ones (CH_2) are in blue.

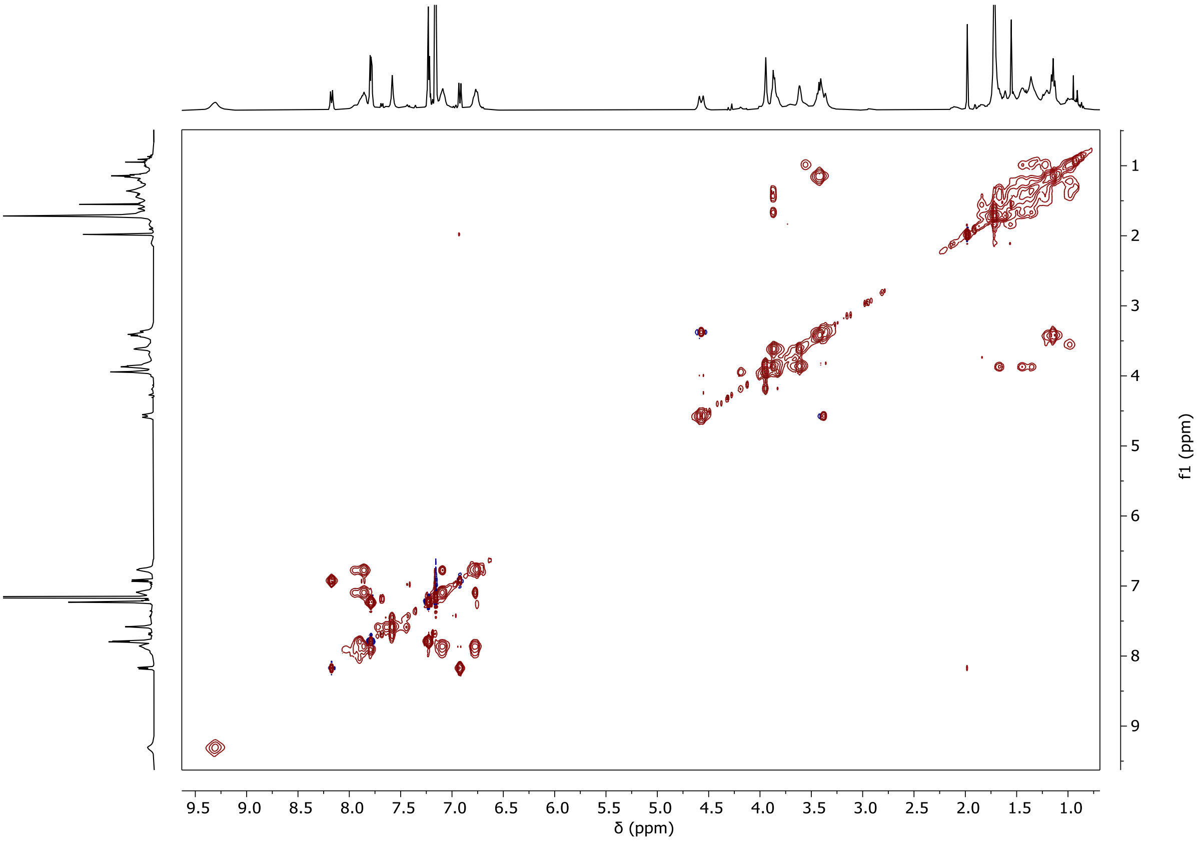

Figure S46: 2D TOCSY NMR spectrum (400 MHz, benzene-d$_6$, MT = 0.06 s) of [3]rotaxane *R*[(**TPU**)$_2$⊃**8**$_{12}$].

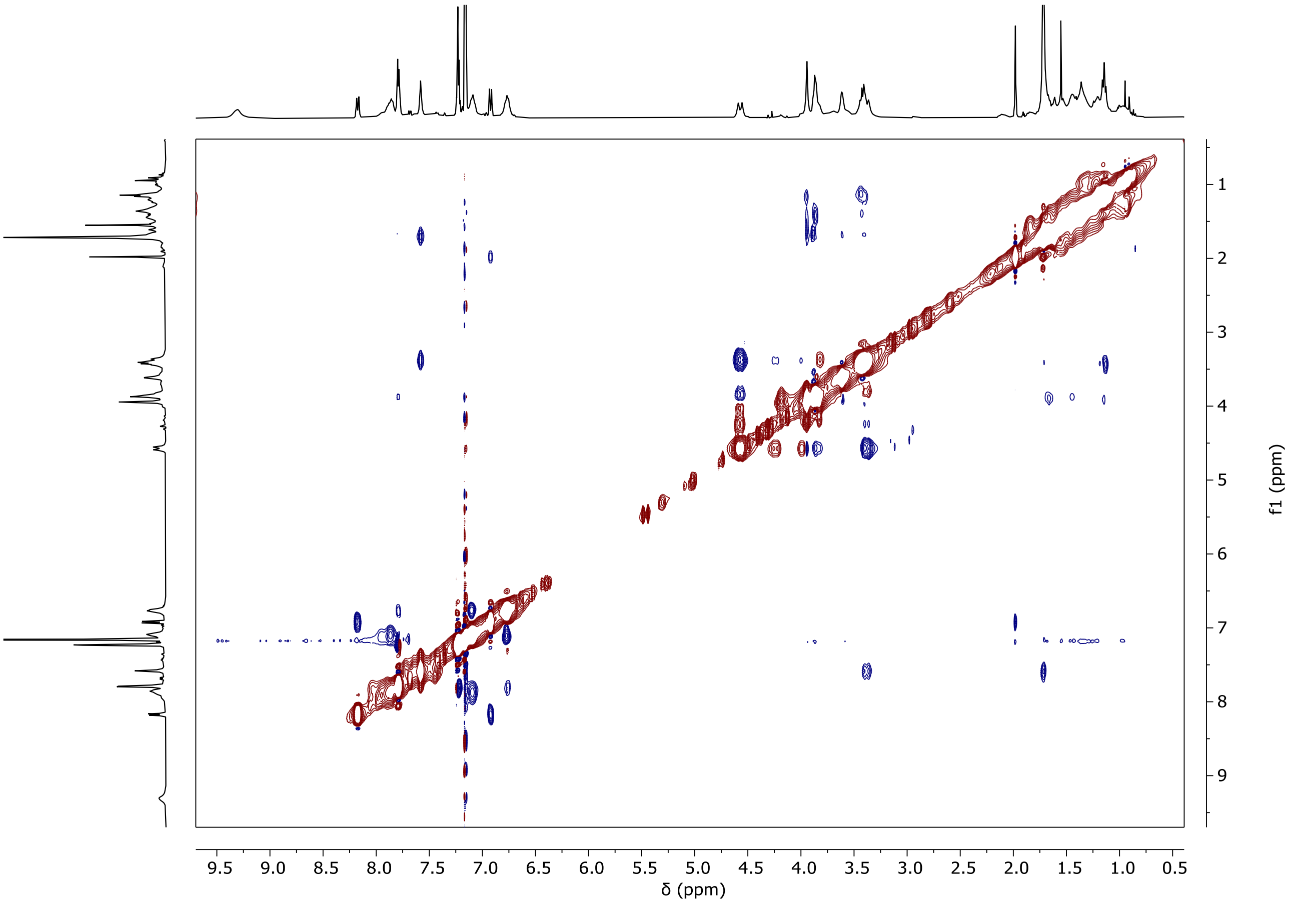

Figure S47: 2D ROESY NMR spectrum (400 MHz, benzene-d$_6$, SL = 200 ms) of [3]rotaxane *R*[(**TPU**)$_2$⊃**8**$_{12}$].

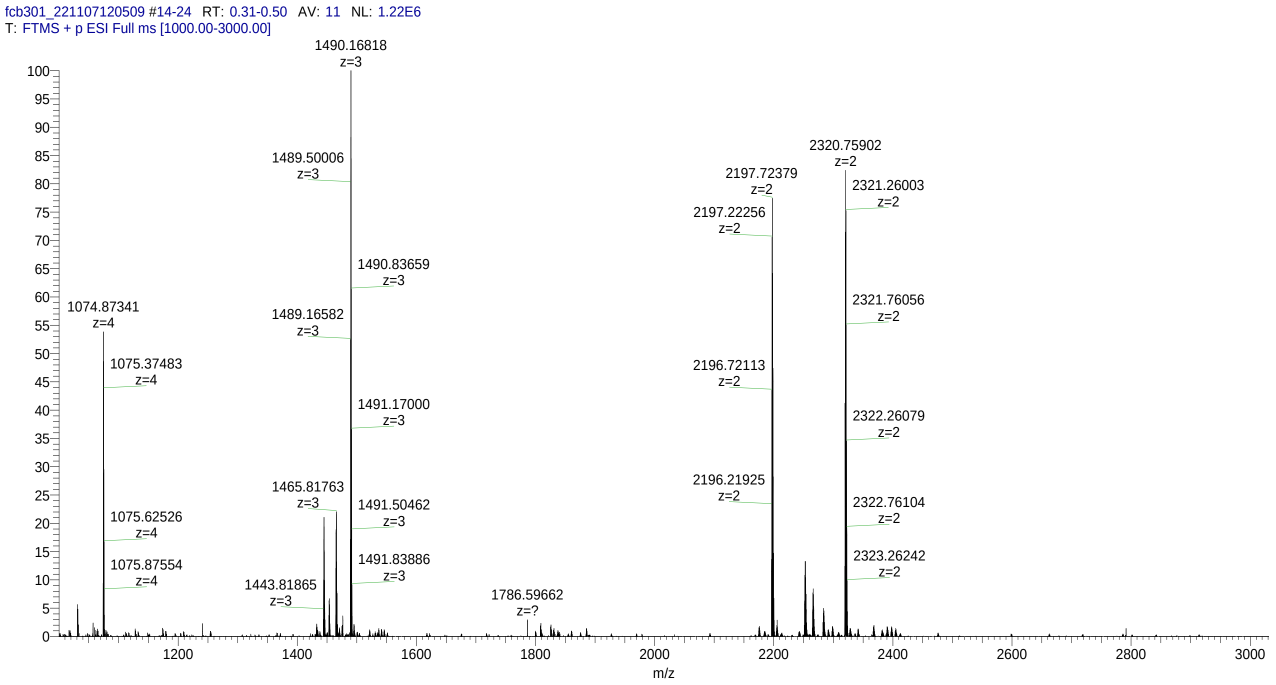

Figure S48: HR-MS (ESI, Orbitrap LQ) spectrum of [*3*]rotaxane R[(TPU)$_2$⊃**8**$_{12}$] showing the quadruple, triply and doubly charged molecular ions.

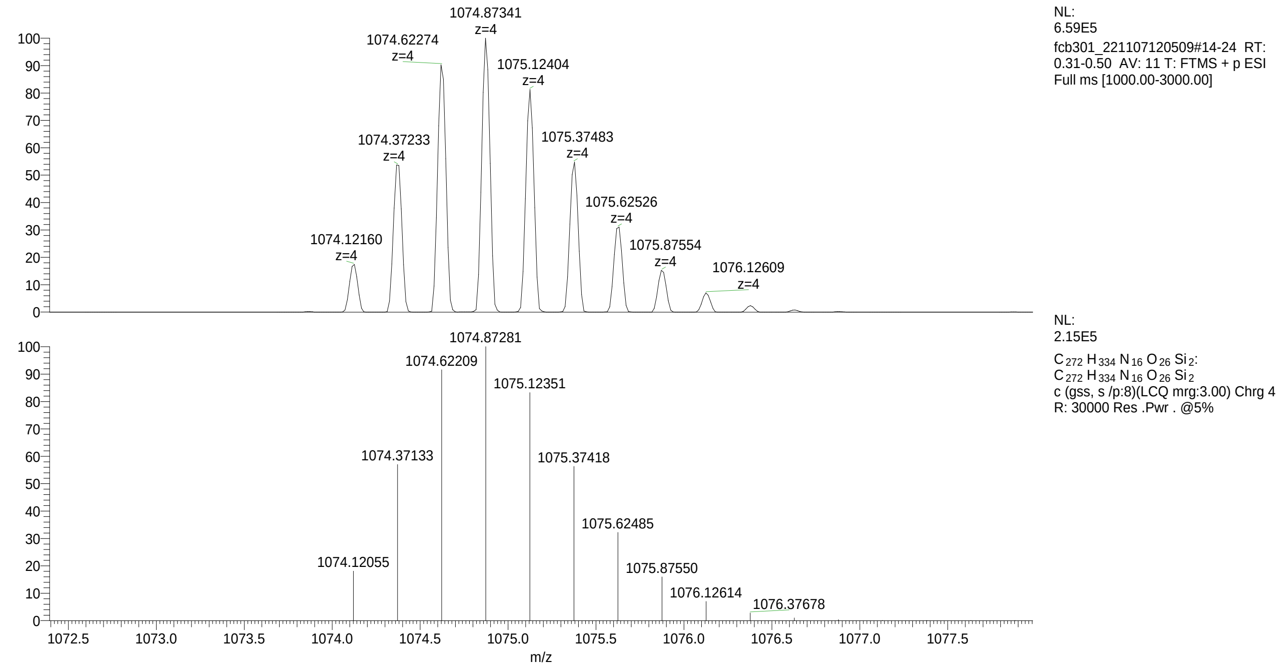

Figure S49: Inset of HR-MS (ESI, Orbitrap LQ) spectrum of compound *R*[(**TPU**)$_2$⊃**8**$_{12}$]: calculated (top) and experimental (down) isotopic distribution for the quadruple charged molecular ion.

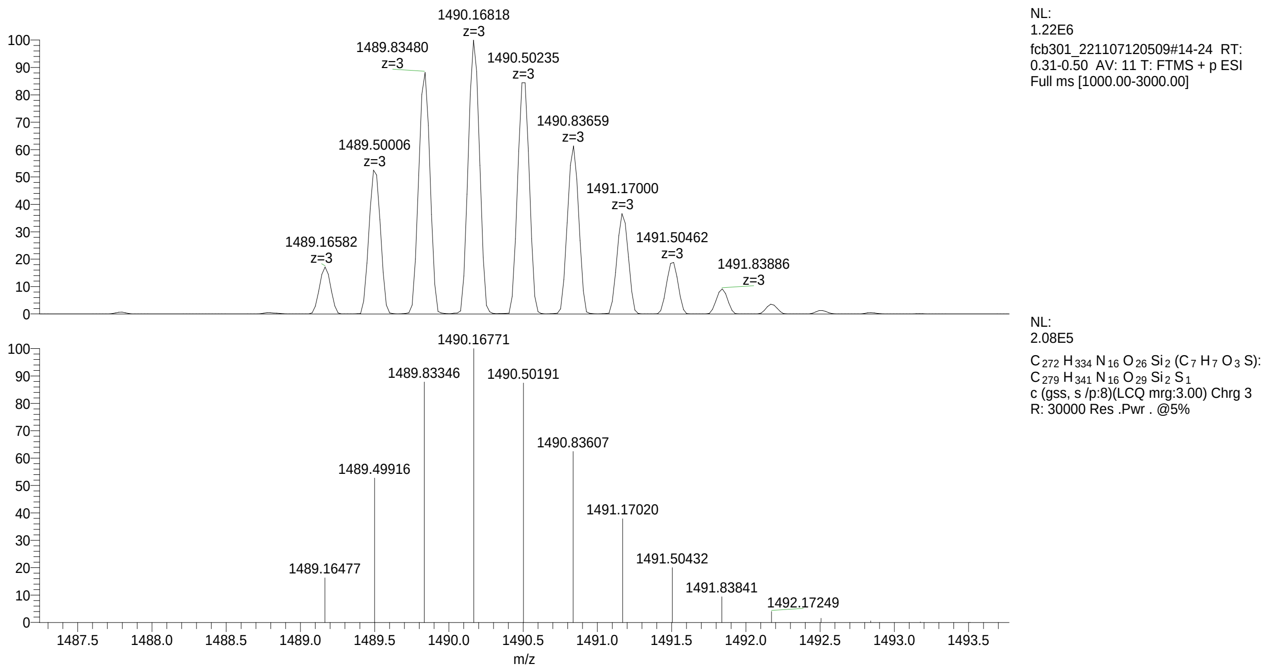

Figure S50: Inset of HR-MS (ESI, Orbitrap LQ) spectrum of compound *R*[(**TPU**)$_2$⊃**8**$_{12}$]: calculated (top) and experimental (down) isotopic distribution for the triply charged molecular ion.

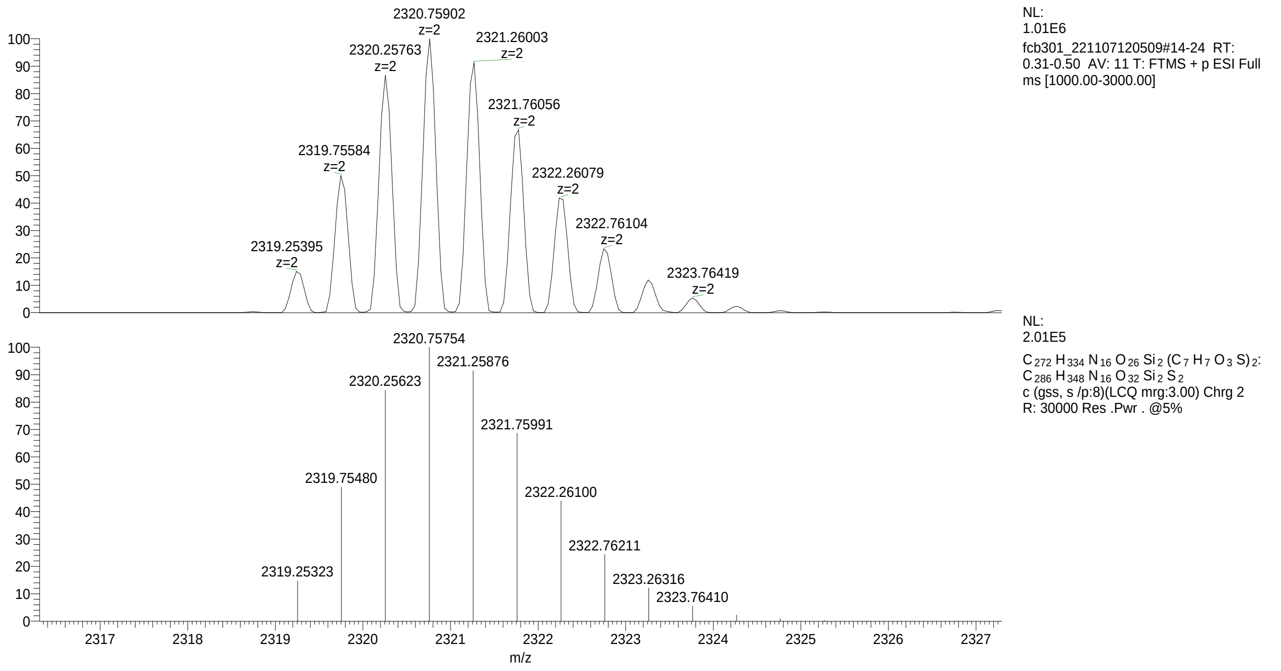

Figure S51: Inset of HR-MS (ESI, Orbitrap LQ) spectrum of compound *R*[(**TPU**)$_2$⊃**8**$_{12}$]: calculated (top) and experimental (down) isotopic distribution for the doubly charged molecular ion.

Characterisation of *P*[(**TPU-ES**)$_2$⊃**5**$_{12}$]

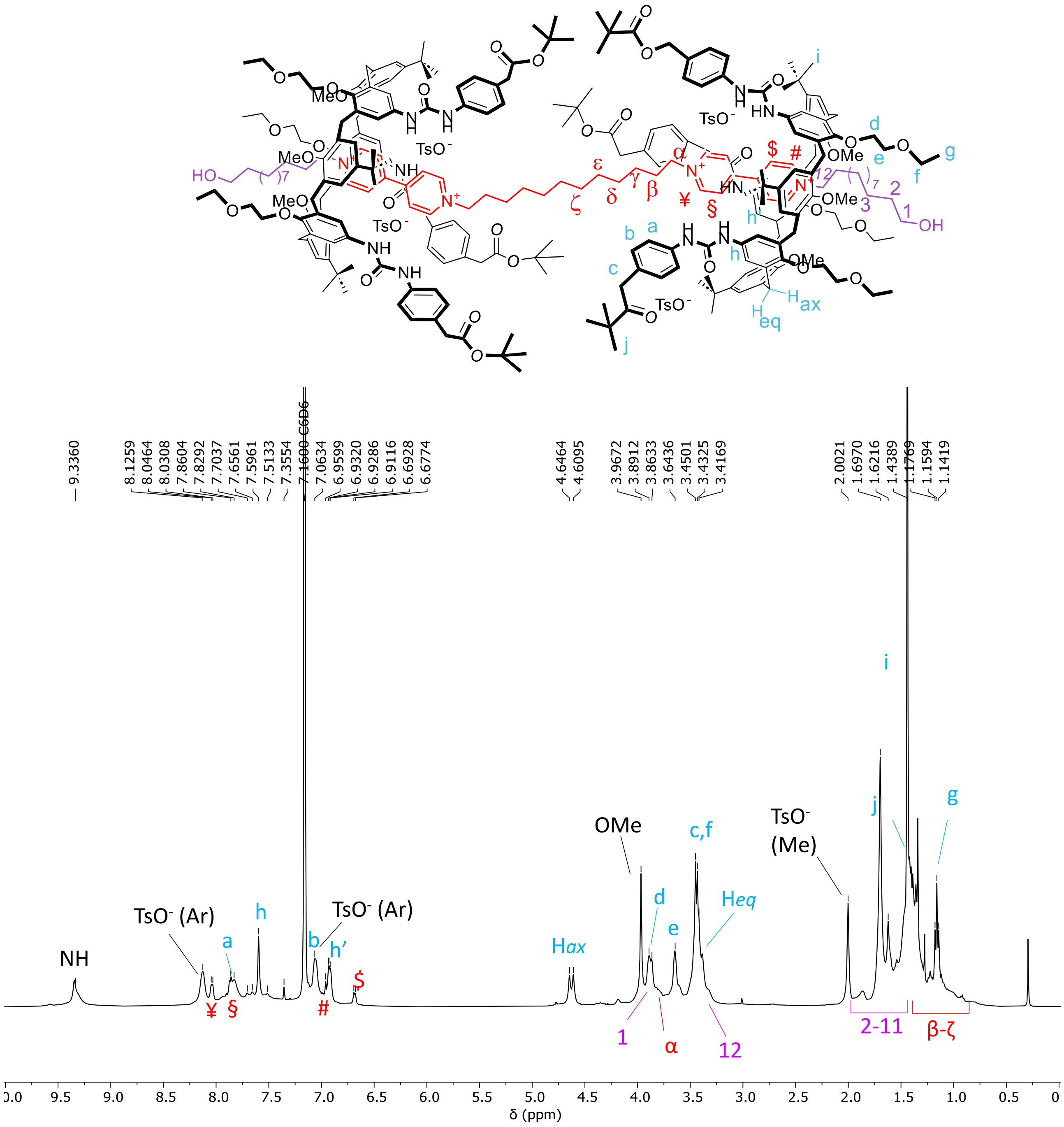

Figure S52: ^{1}H NMR spectrum (400 MHz, benzene-d$_6$) of [3]pseudorotaxane *P*[(**TPU-ES**)$_2$⊃**5**$_{12}$].

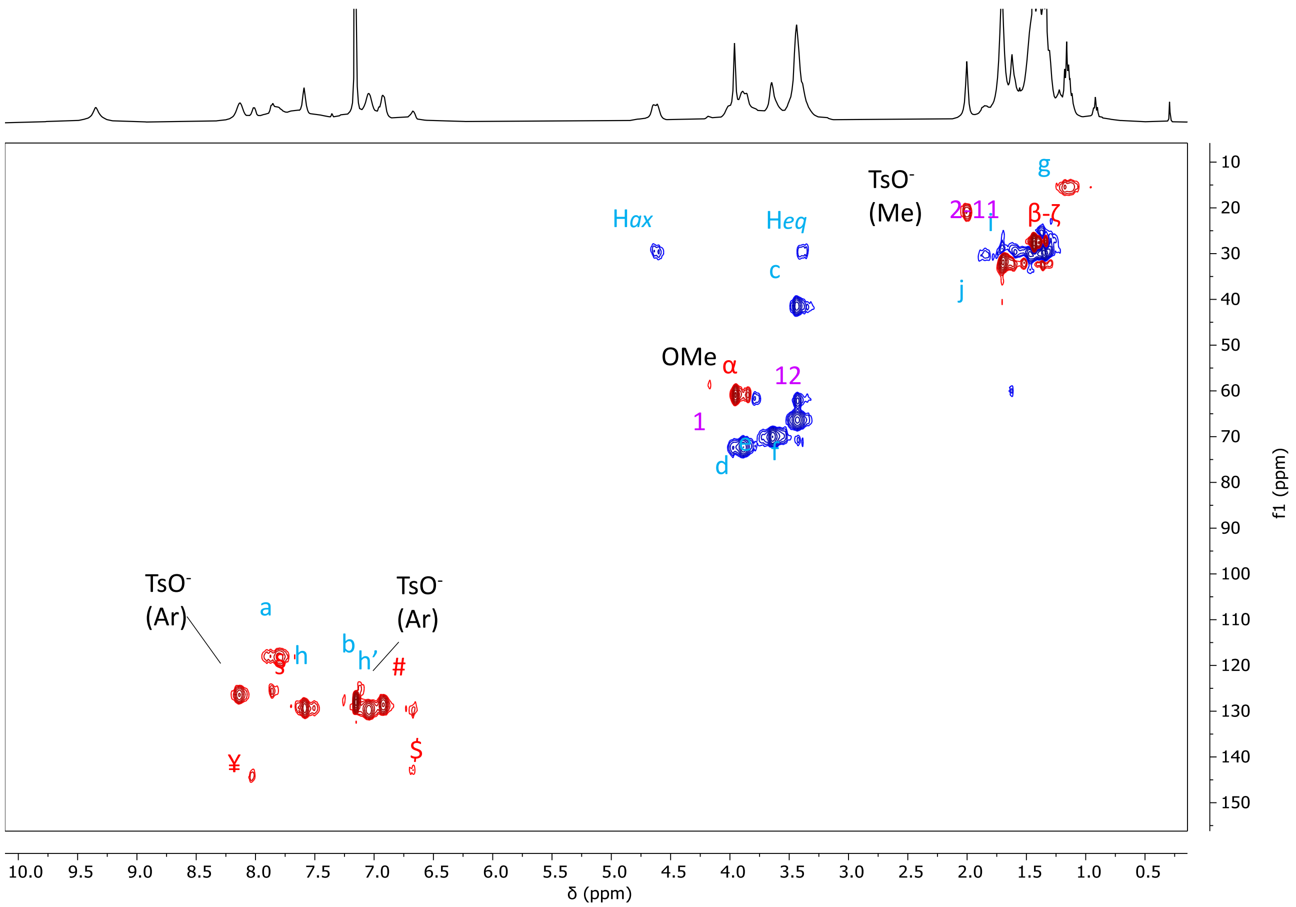

Figure S53: Edited HSQC NMR spectrum (400 MHz, benzene-d_6) of [3]pseudorotaxane *P*[(**TPU-ES**)$_2$⊃**5**$_{12}$]. Positive peaks (CH_3 and CH) are shown in red, while negative ones (CH_2) are in blue.

Characterisation of *R*[(**TPU-ES**)$_2$⊃**7**$_{12}$]

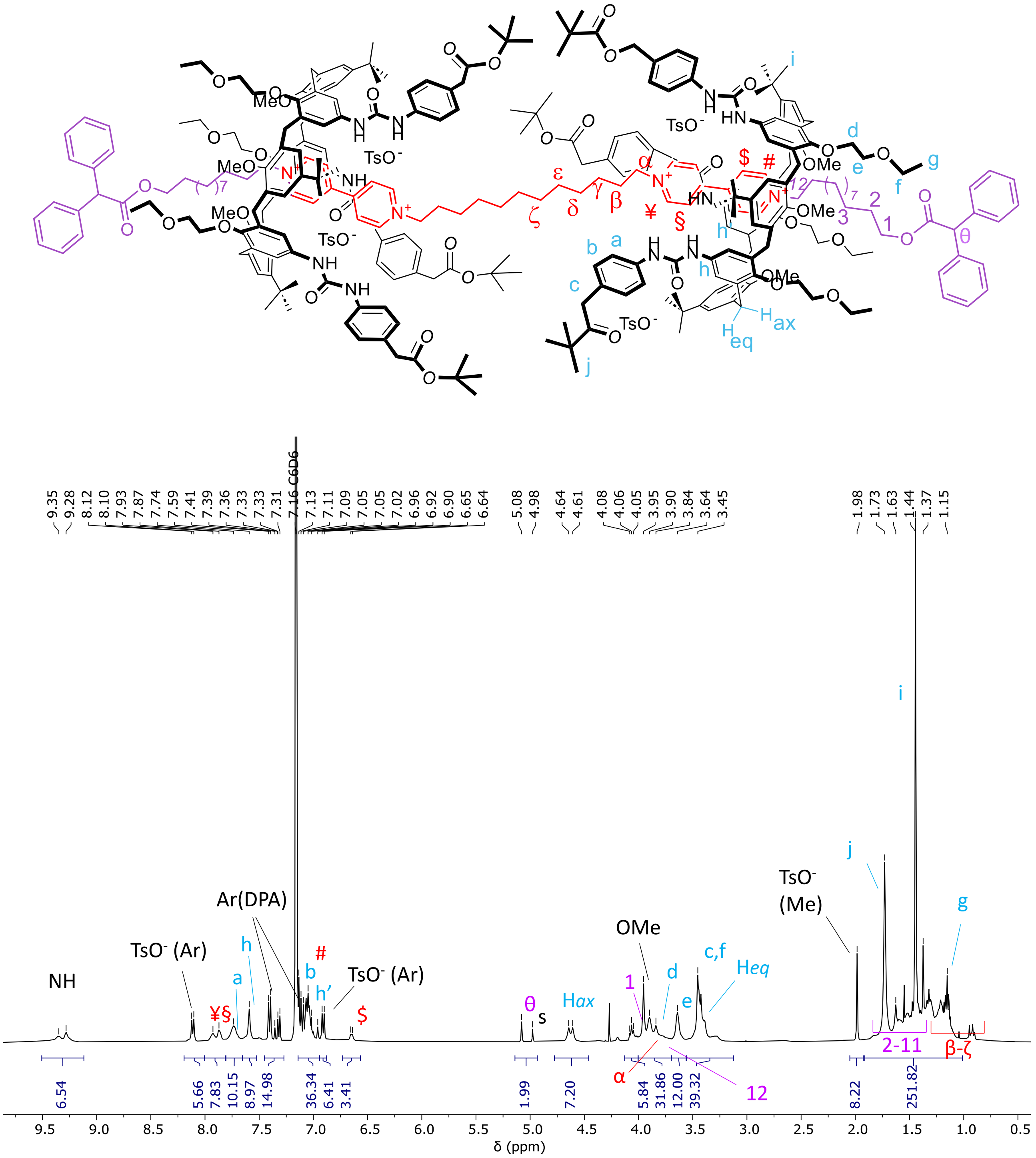

Figure S54: ^{1}H NMR spectrum (400 MHz, benzene-d$_6$) of [3]rotaxane *R*[(**TPU-ES**)$_2$⊃**7**$_{12}$]. The resonance with the S label at 5.02 ppm is associated with the signal of the diphenylacetate methine proton that exchanged the tosylates upon axle stoppering.

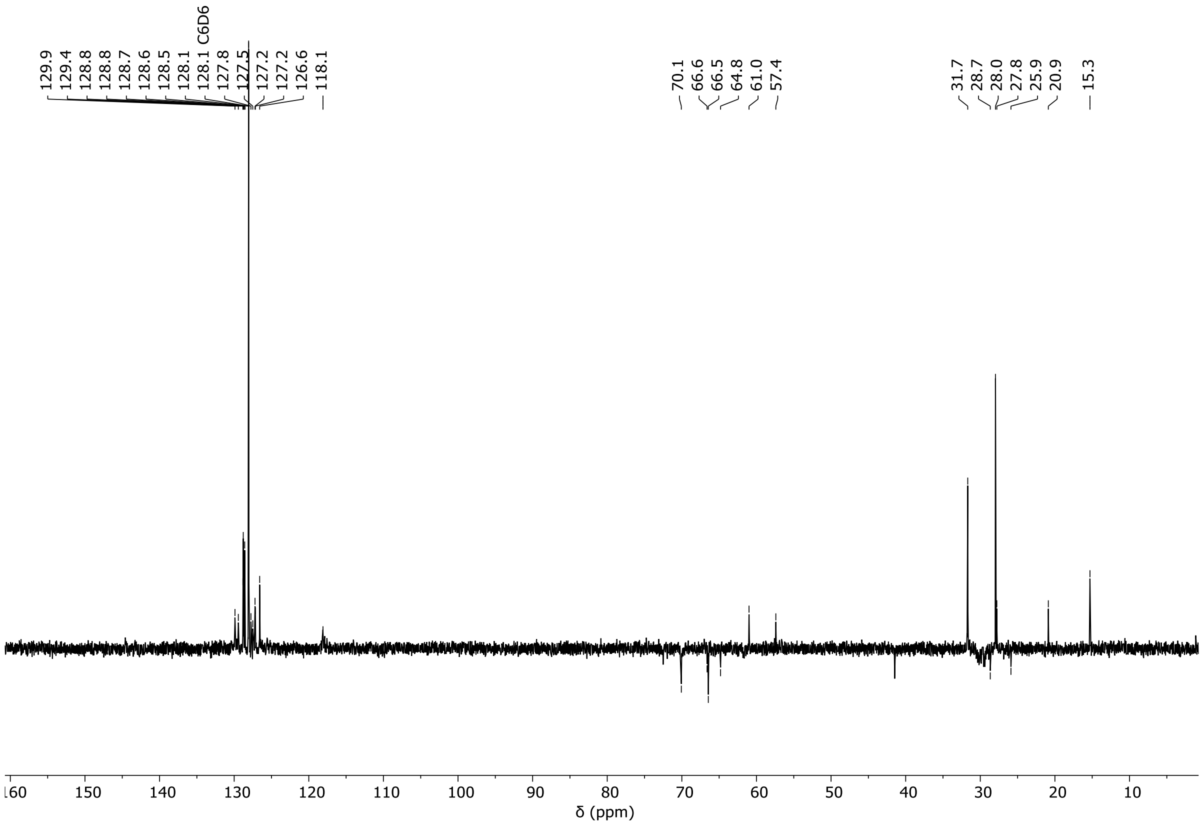

Figure S55: ^{13}C-APT NMR spectrum (100 MHz, benzene-d$_6$) of [3]rotaxane *R*[(**TPU-ES**)$_2$⊃**7**$_{12}$].

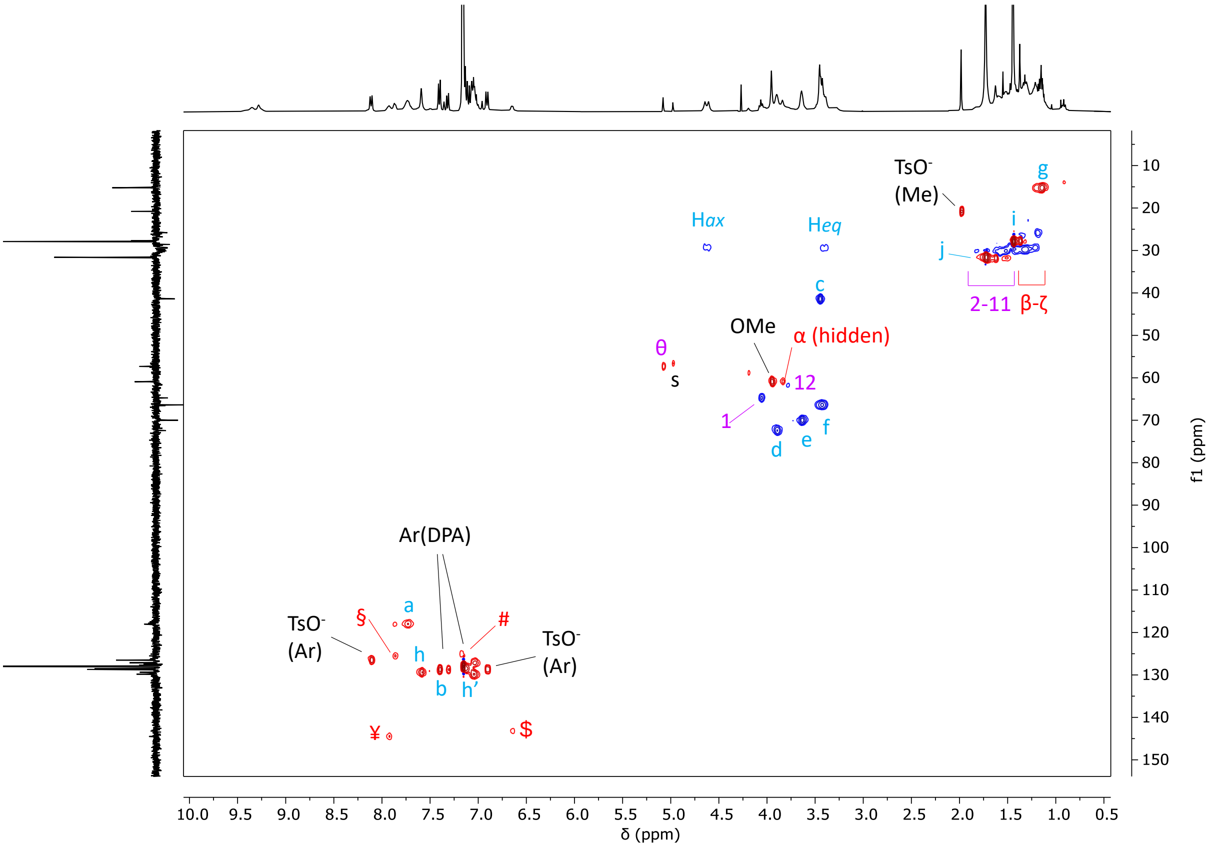

Figure S56: Edited HSQC NMR spectrum (400 MHz, benzene-d$_6$) of [3]rotaxane *R*[(**TPU-ES**)$_2$⊃**7**$_{12}$]. Positive peaks (CH_3 and CH) are shown in red, while negative ones (CH_2) are in blue.

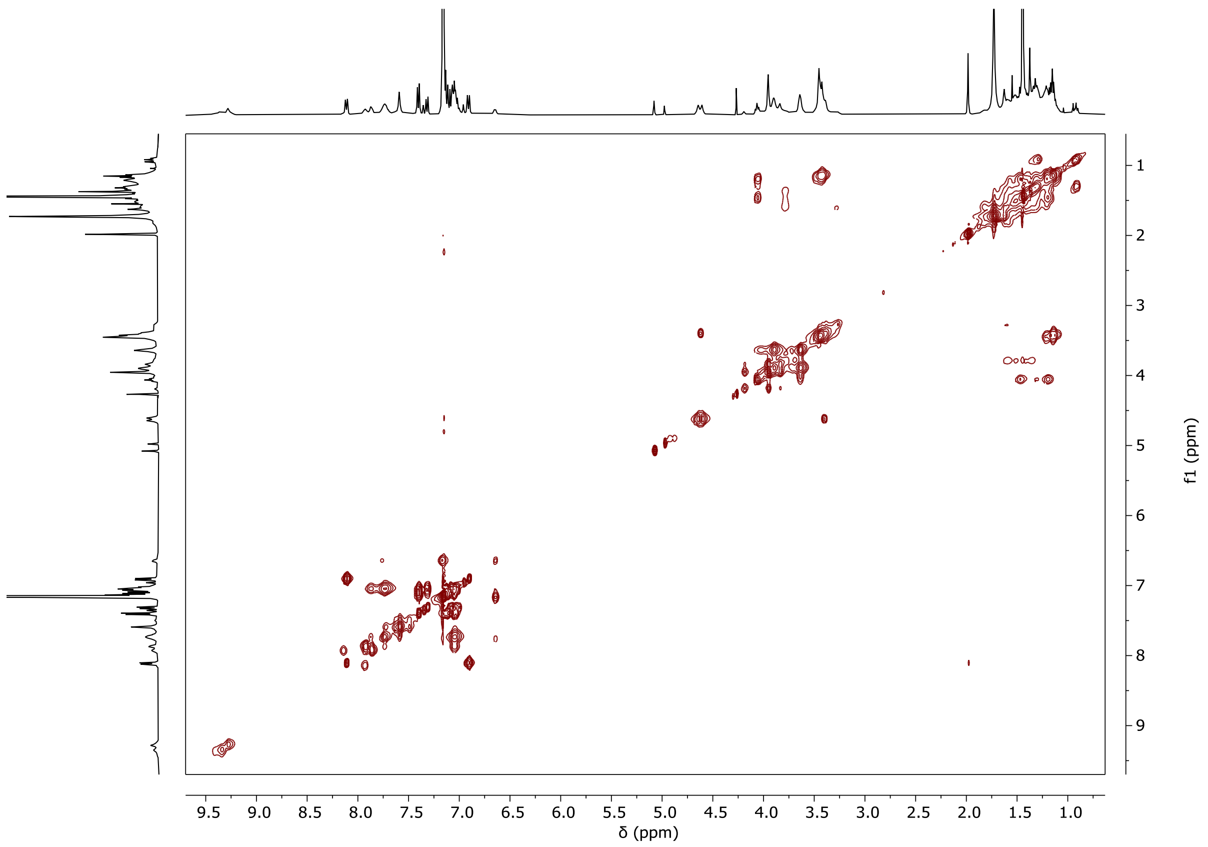

Figure S57: 2D TOCSY NMR spectrum (400 MHz, benzene-d$_6$, MT = 0.06 s) of [3]rotaxane *R*[(**TPU-ES**)$_2$⊃**7**$_{12}$].

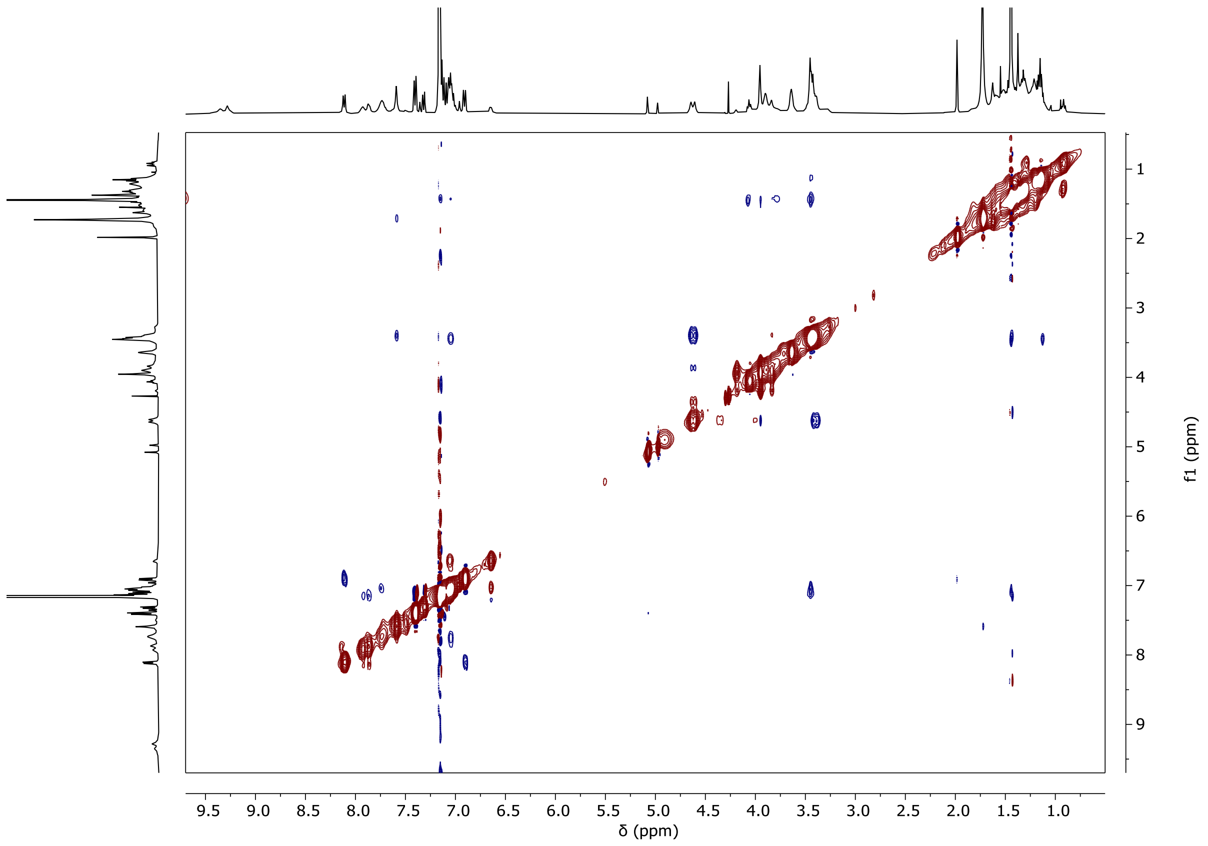

Figure S58: 2D ROESY NMR spectrum (400 MHz, benzene-d$_6$, SL = 200 ms) of [3]rotaxane *R*[(**TPU-ES**)$_2$⊃**7**$_{12}$].

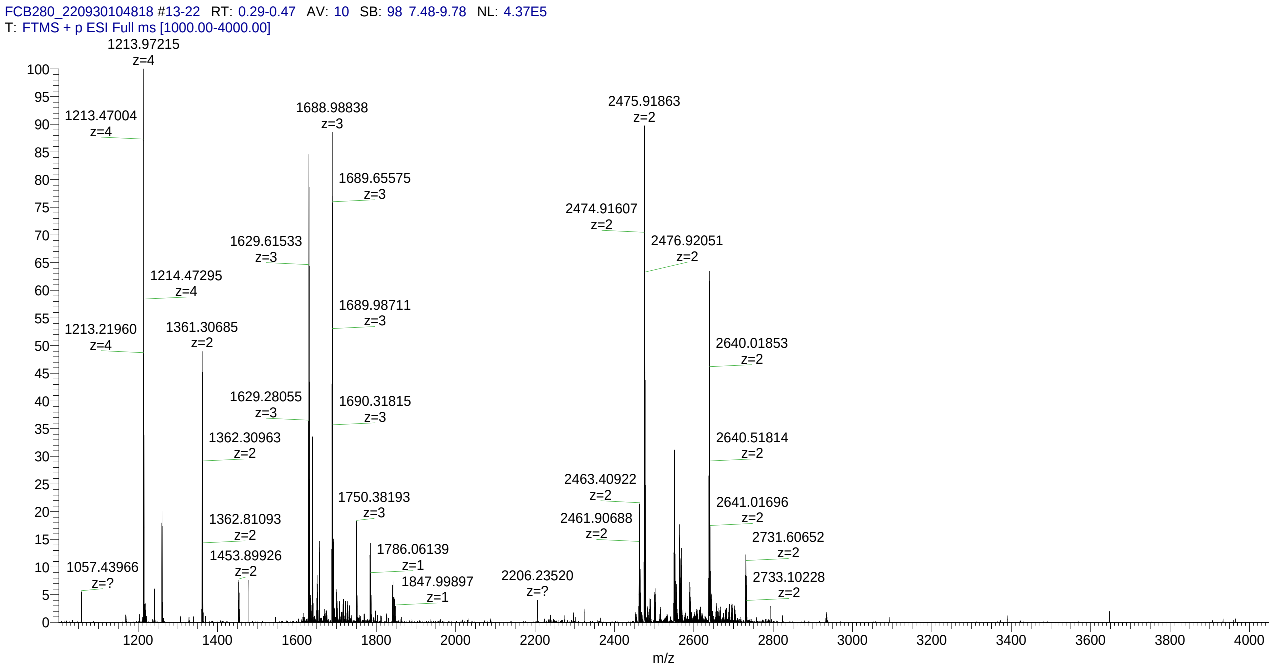

Figure S59: HR-MS (ESI, Orbitrap LQ) spectrum of [*3*]rotaxane *R*[(**TPU-ES**)2⊃**7**12] showing the quadruple, triply and doubly charged molecular.

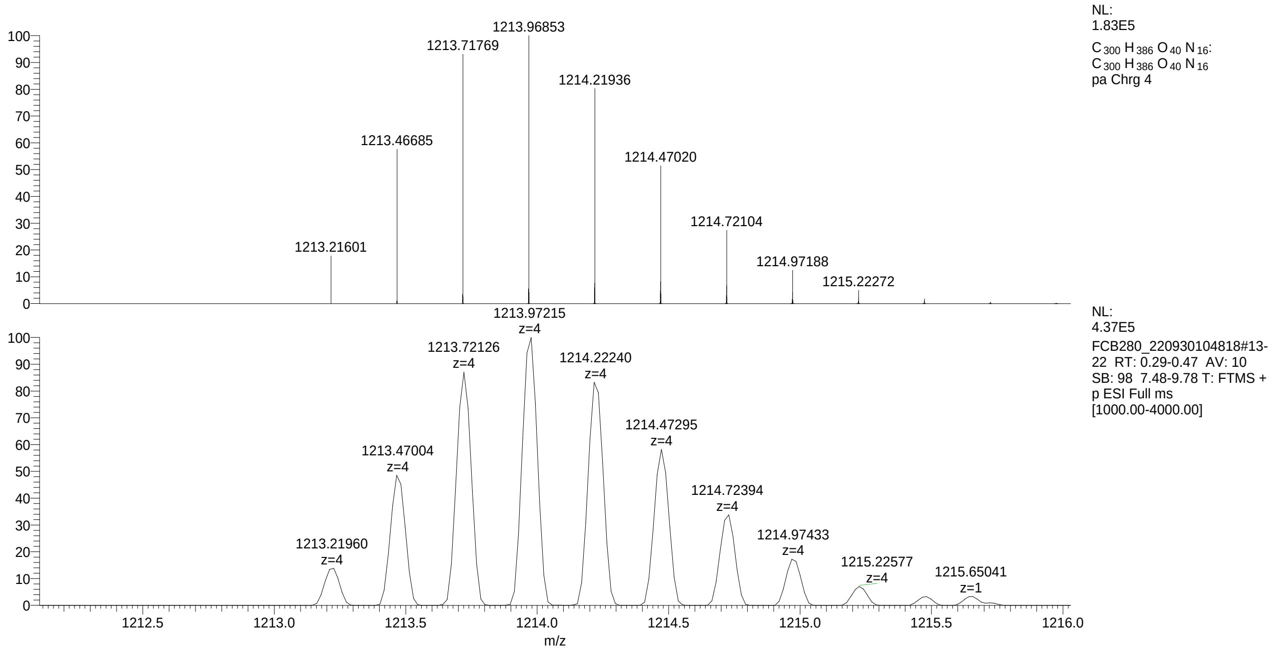

Figure S60: Inset of HR-MS (ESI, Orbitrap LQ) spectrum of compound *R*[(**TPU-ES**)2⊃**7**12]: calculated (top) and experimental (down) isotopic distribution for the quadruple charged molecular ion.

Characterisation of *R*[(**TPU-ES**)$_2$⊃**8**$_{12}$]

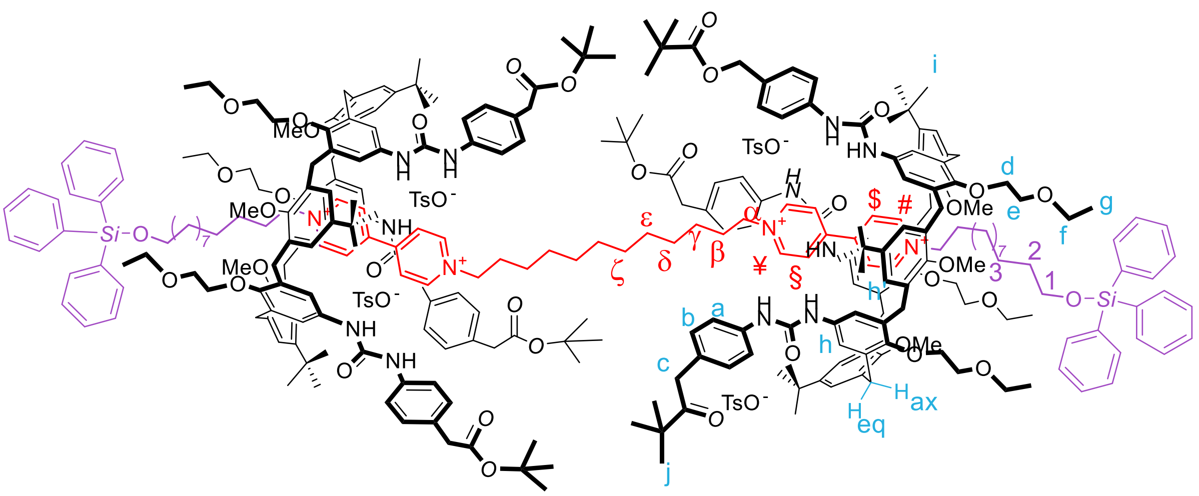

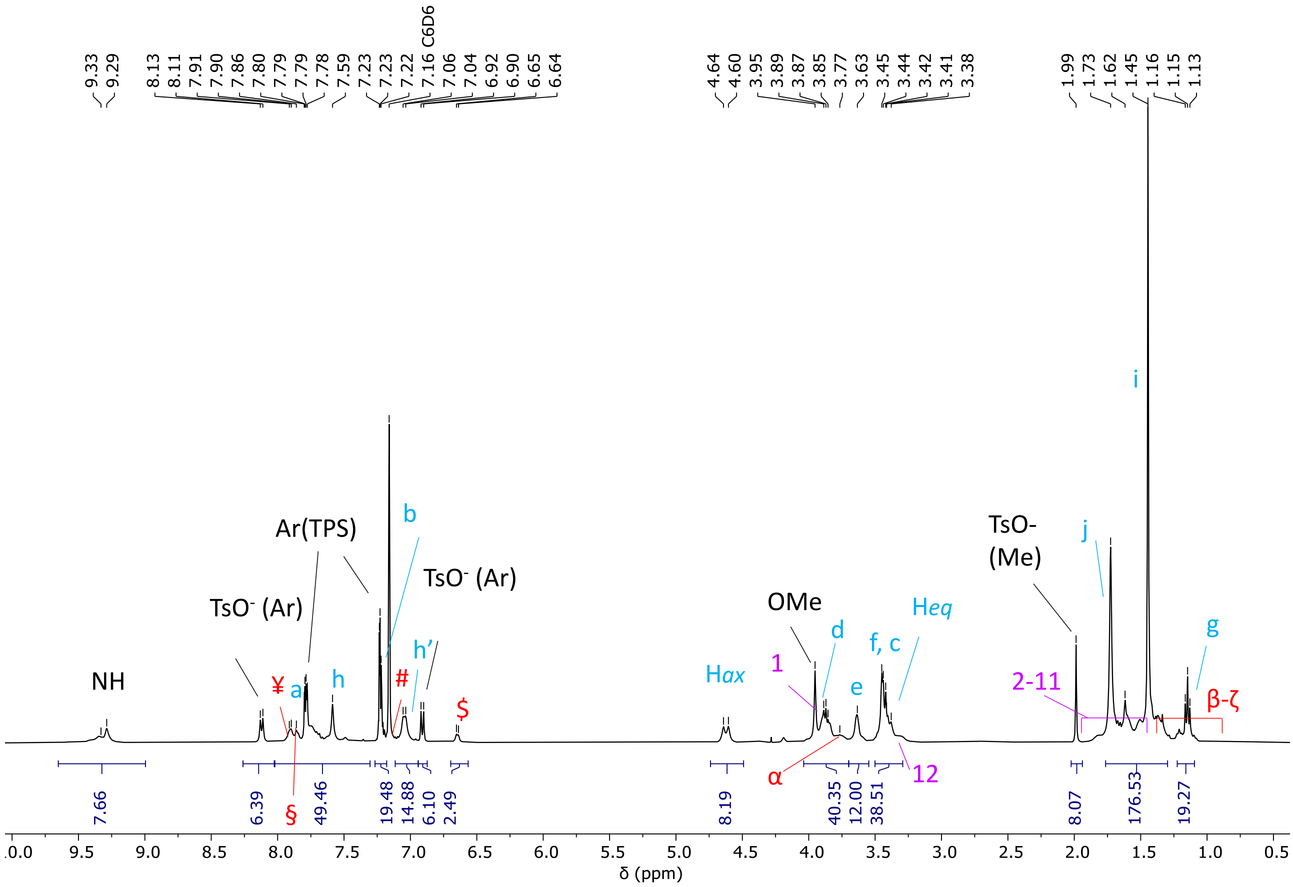

Figure S61: ^{1}H NMR spectrum (400 MHz, benzene-d$_6$) of [3]rotaxane *R*[(**TPU-ES**)$_2$⊃**8**$_{12}$].

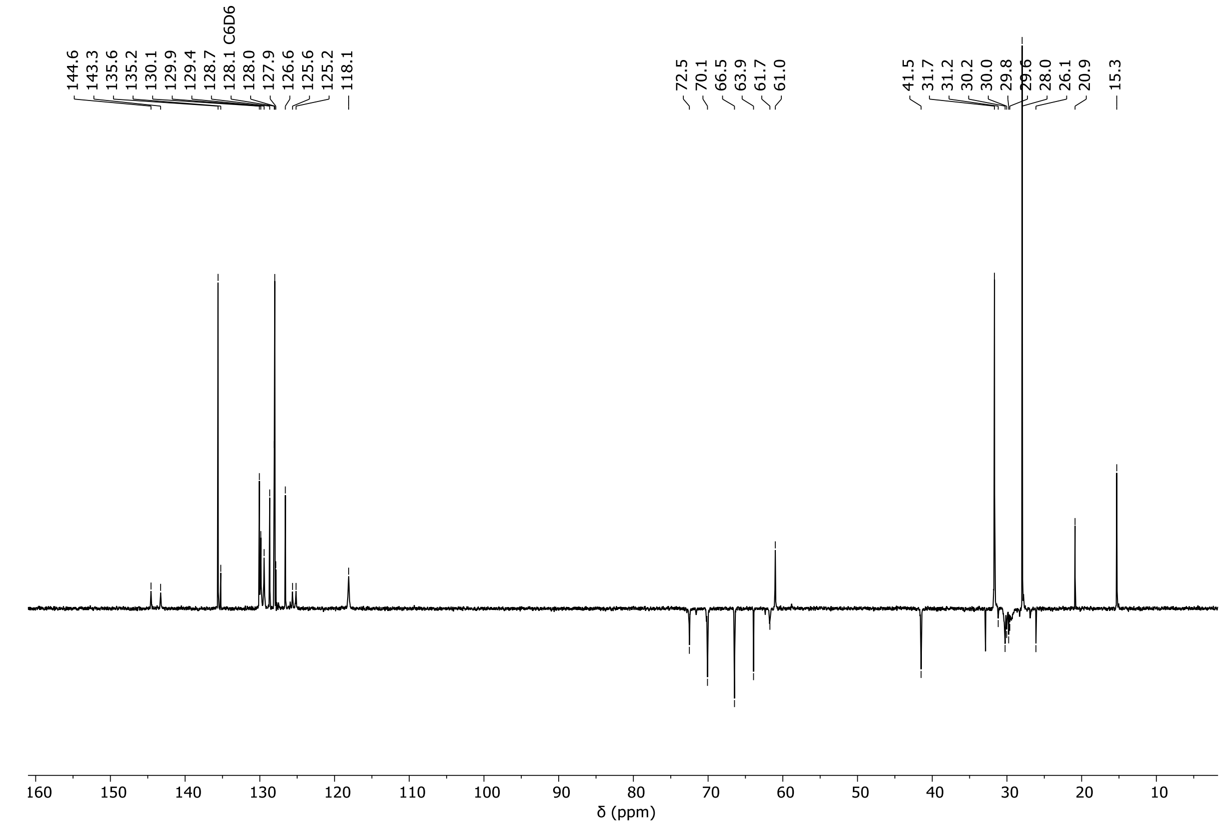

Figure S62: ^{13}C-APT NMR spectrum (100 MHz, benzene-d$_6$) of [3]rotaxane *R*[(**TPU-ES**)$_2$⊃**8**$_{12}$]

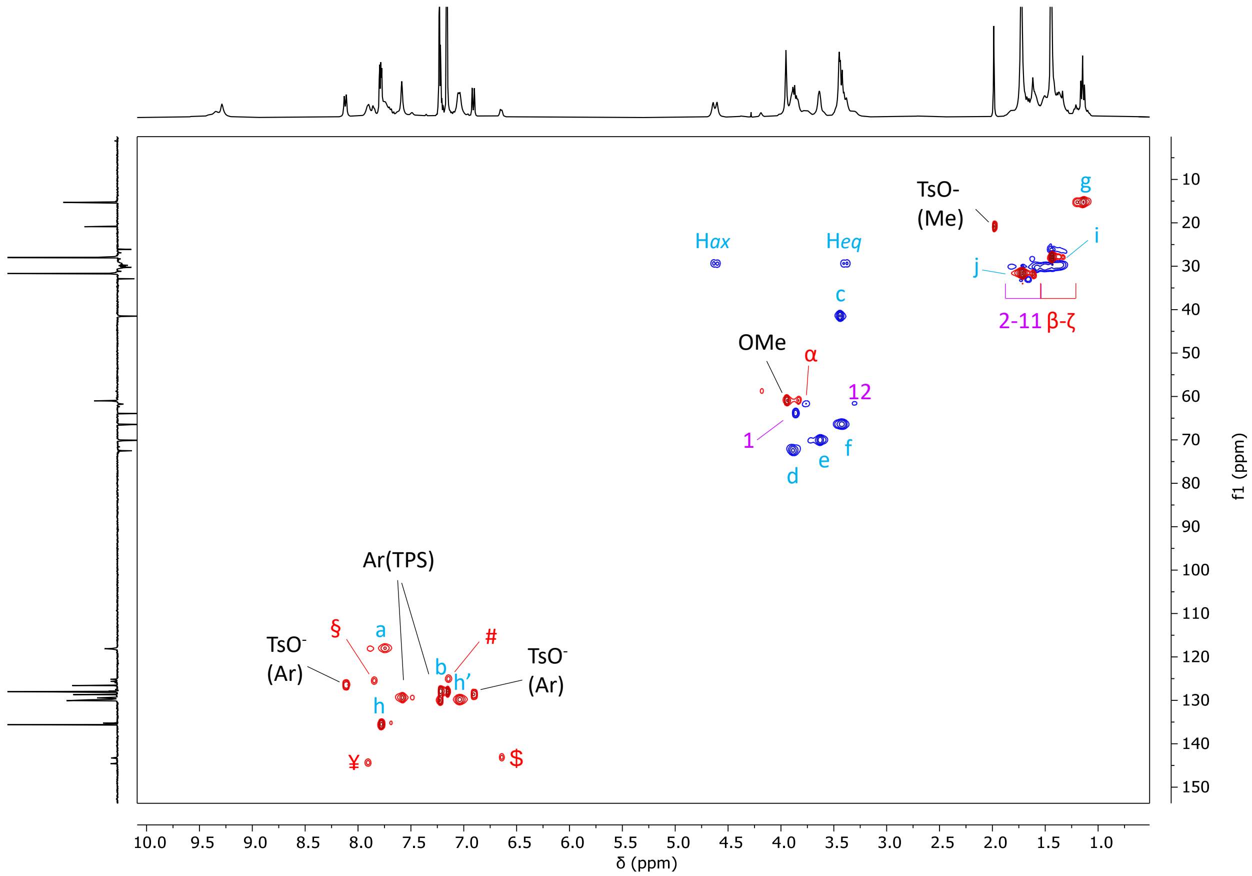

Figure S63: Edited HSQC NMR spectrum (400 MHz, benzene-d$_6$) of [3]rotaxane *R*[(**TPU-ES**)$_2$⊃**8**$_{12}$]. Positive peaks (CH_3 and CH) are shown in red, while negative ones (CH_2) are in blue.

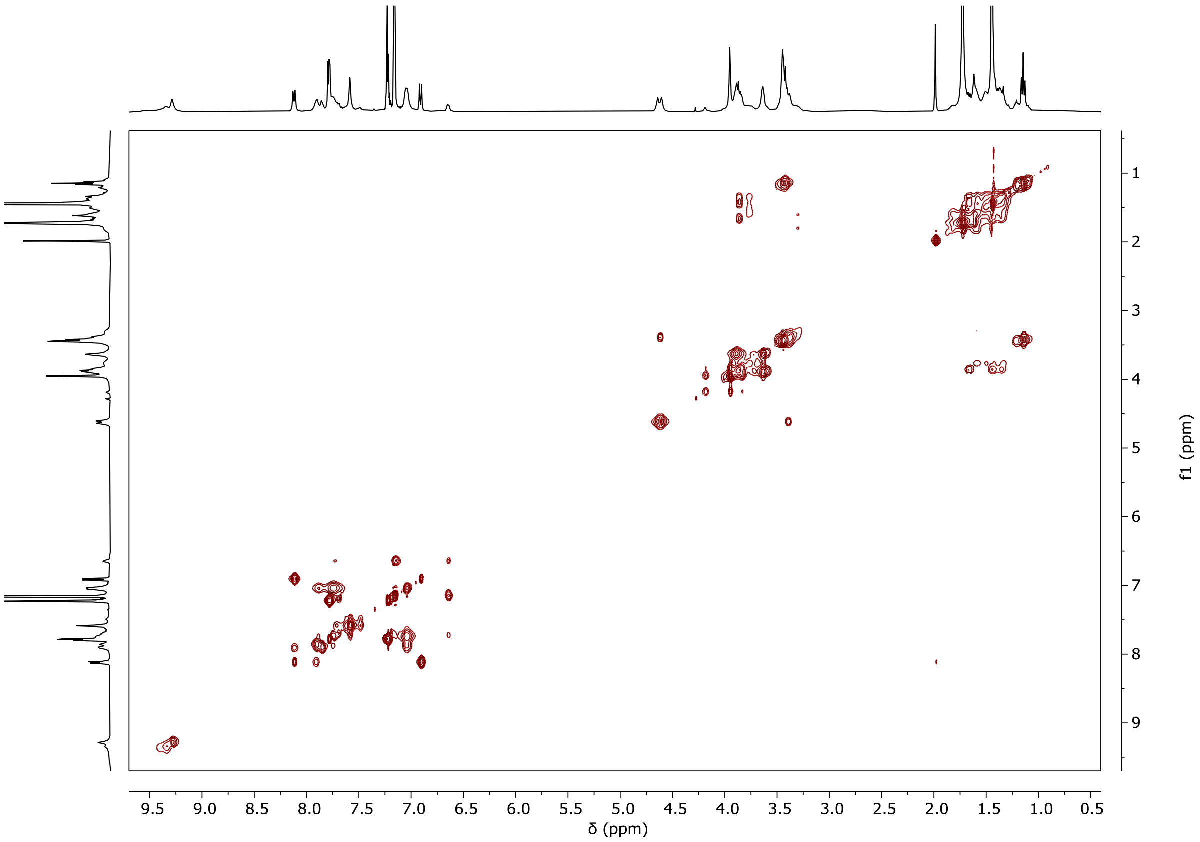

Figure S64: 2D TOCSY NMR spectrum (400 MHz, benzene-d6) of [3]rotaxane *R*[(**TPU-ES**)$_2$⊃**8**$_{12}$].

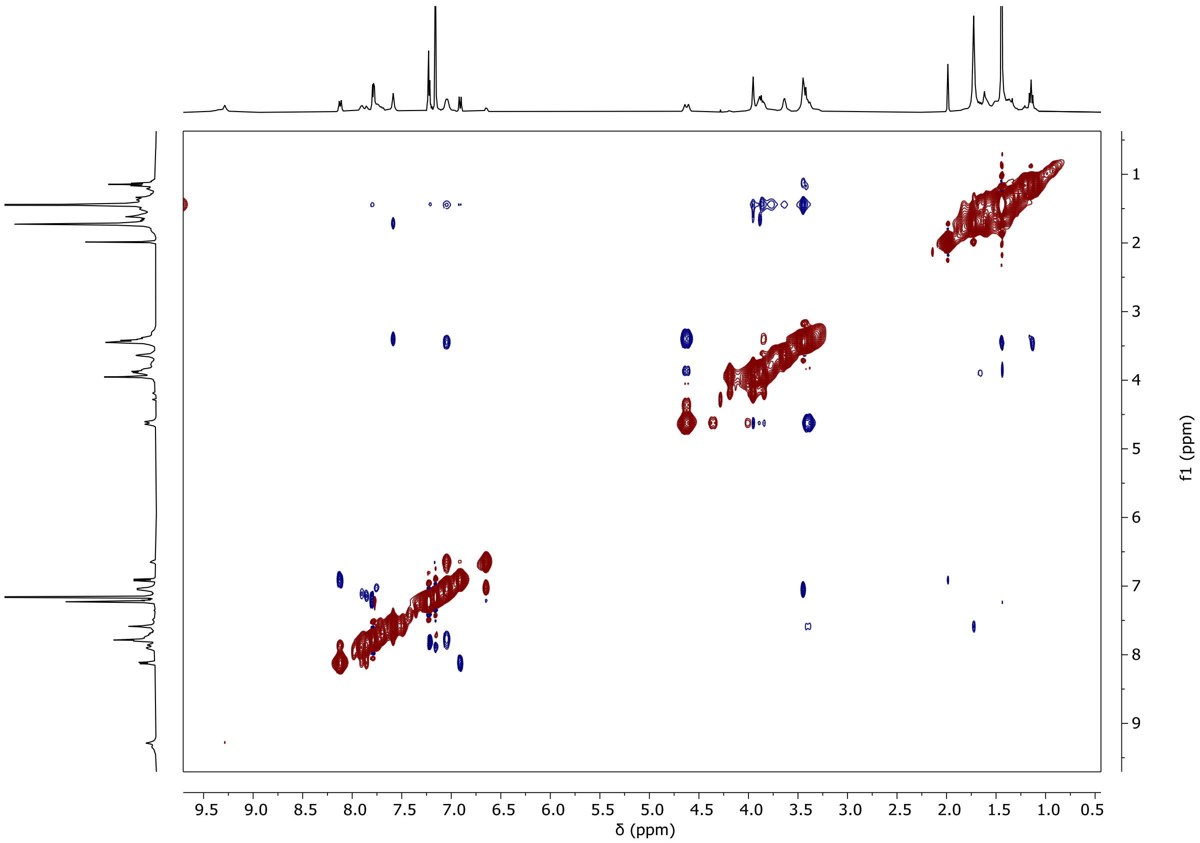

Figure S65: 2D ROESY NMR spectrum (400 MHz, benzene-d6, SL = 200 ms) of [3]rotaxane *R*[(**TPU-ES**)$_2$⊃**8**$_{12}$].

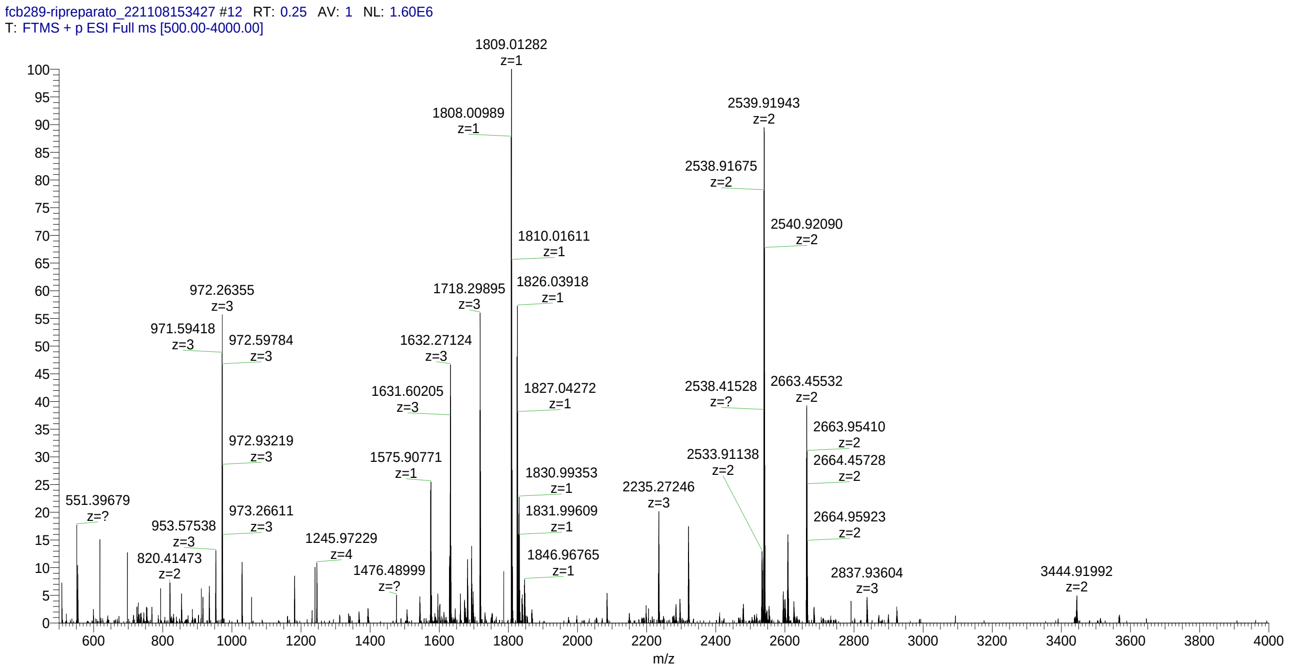

Figure S66: HR-MS (ESI, Orbitrap LQ) spectrum of [3]rotaxane *R*[(**TPU-ES**)$_2$⊃**8**$_{12}$] showing the quadruple, triply and doubly charged molecular.

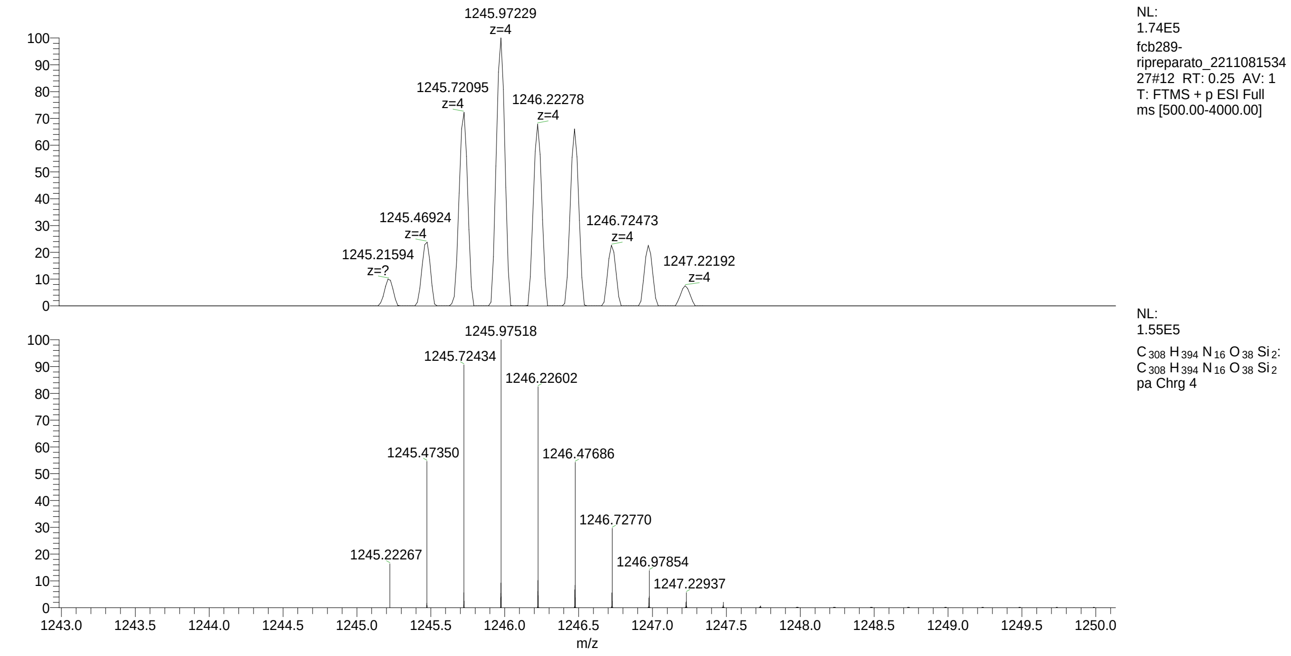

Figure S67: Inset of HR-MS (ESI, Orbitrap LQ) spectrum of compound *R*[(**TPU-ES**)$_2$⊃**8**$_{12}$]: calculated (top) and experimental (down) isotopic distribution for the quadruple charged molecular ion.

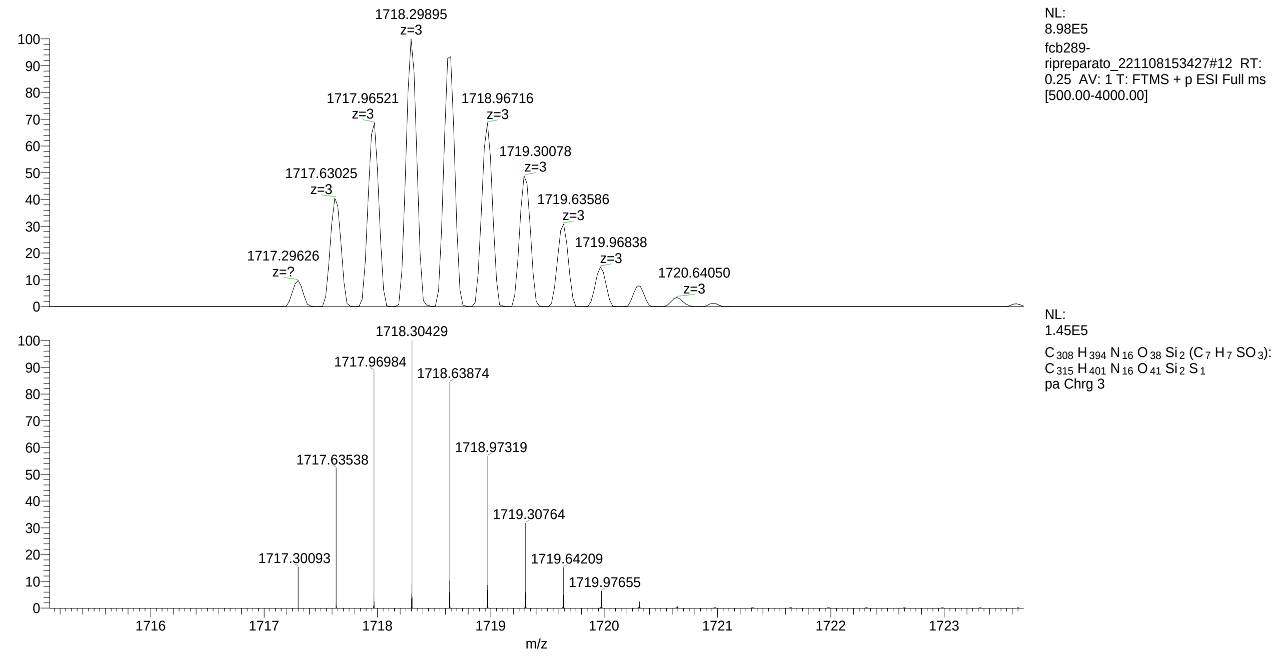

Figure S68: Inset of HR-MS (ESI, Orbitrap LQ) spectrum of compound R[(**TPU-ES**)$_2$⊃**8**$_{12}$]: calculated (top) and experimental (down) isotopic distribution for the triply charged molecular ion.

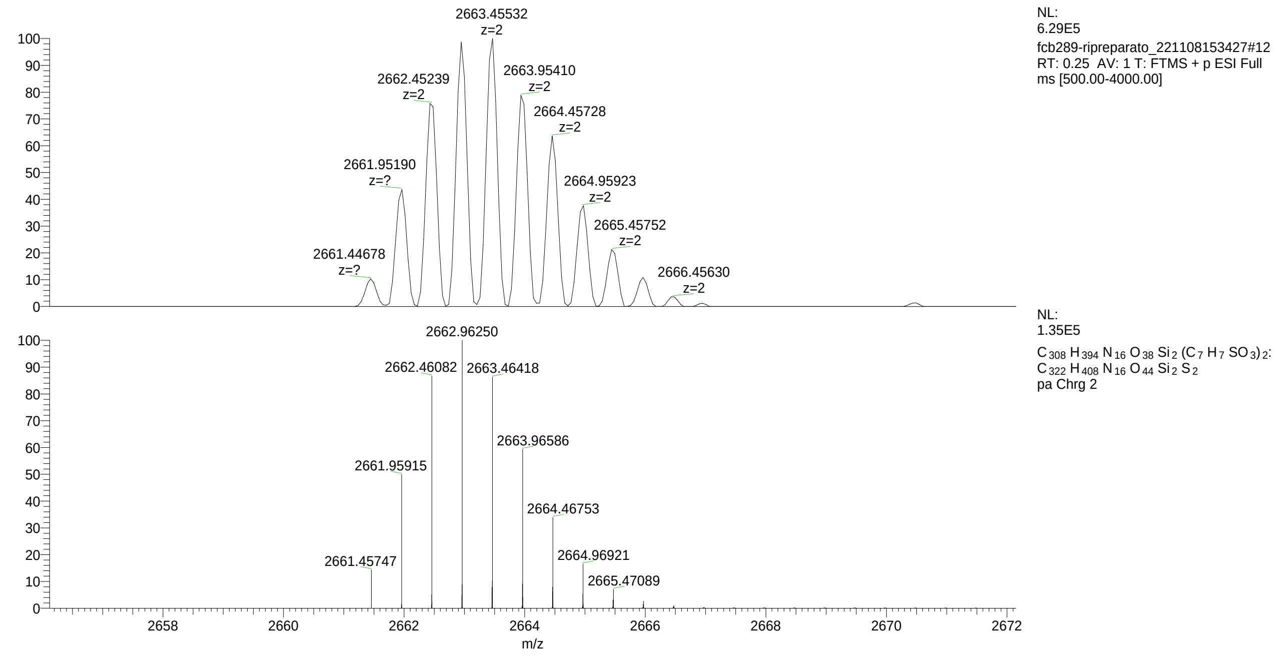

Figure S69: Inset of HR-MS (ESI, Orbitrap LQ) spectrum of compound R[(**TPU-ES**)$_2$⊃**8**$_{12}$]: calculated (top) and experimental (down) isotopic distribution for the doubly charged molecular ion.

Characterisation of *R*[(**TPU-AC**)$_2$⊃**7**$_{12}$]

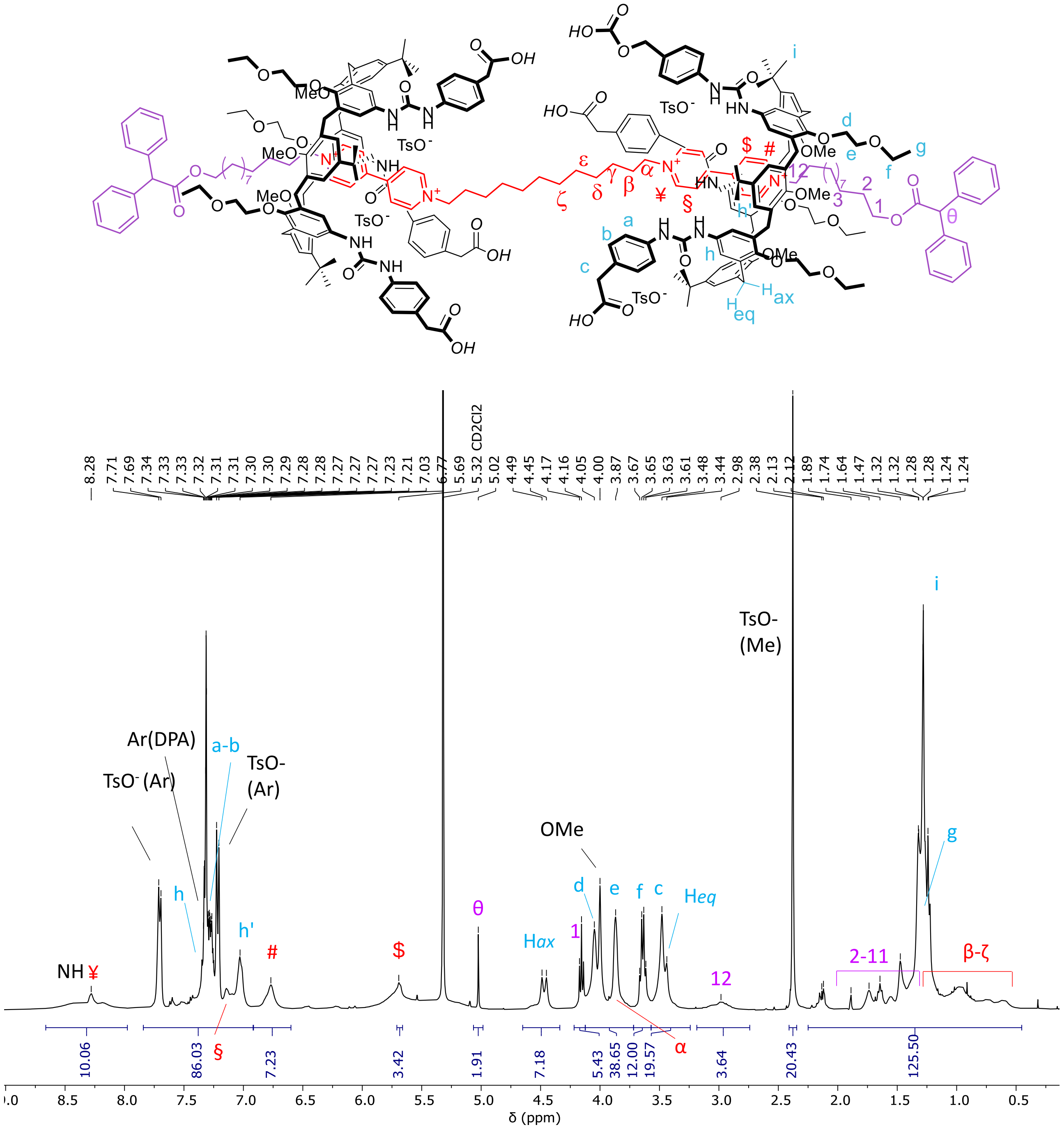

Figure S70: ^{1}H NMR spectrum (400 MHz, CD_2Cl_2) of [3]rotaxane *R*[(**TPU-AC**)$_2$⊃**7**$_{12}$].

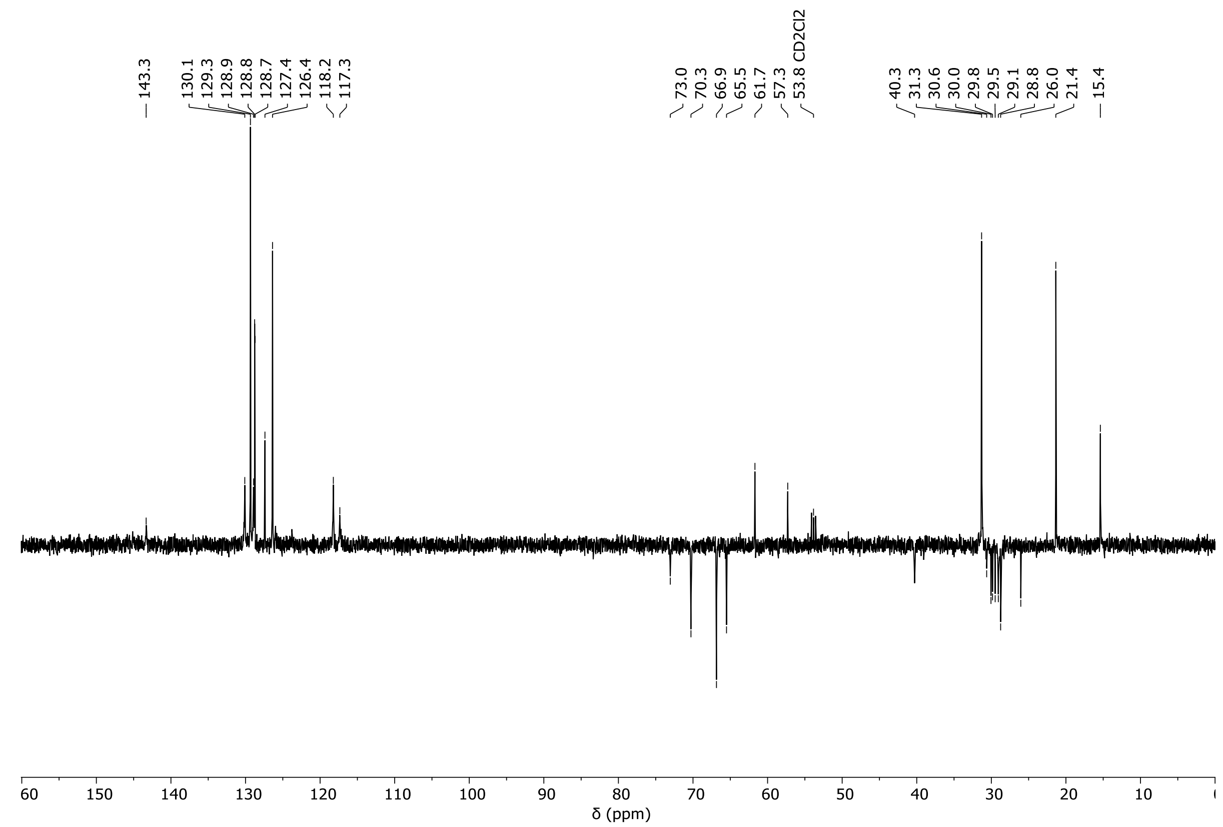

Figure S71: ^{13}C NMR spectrum (100 MHz, CD_2Cl_2) of [3]rotaxane *R*[(**TPU-AC**)$_2$⊃**7**$_{12}$].

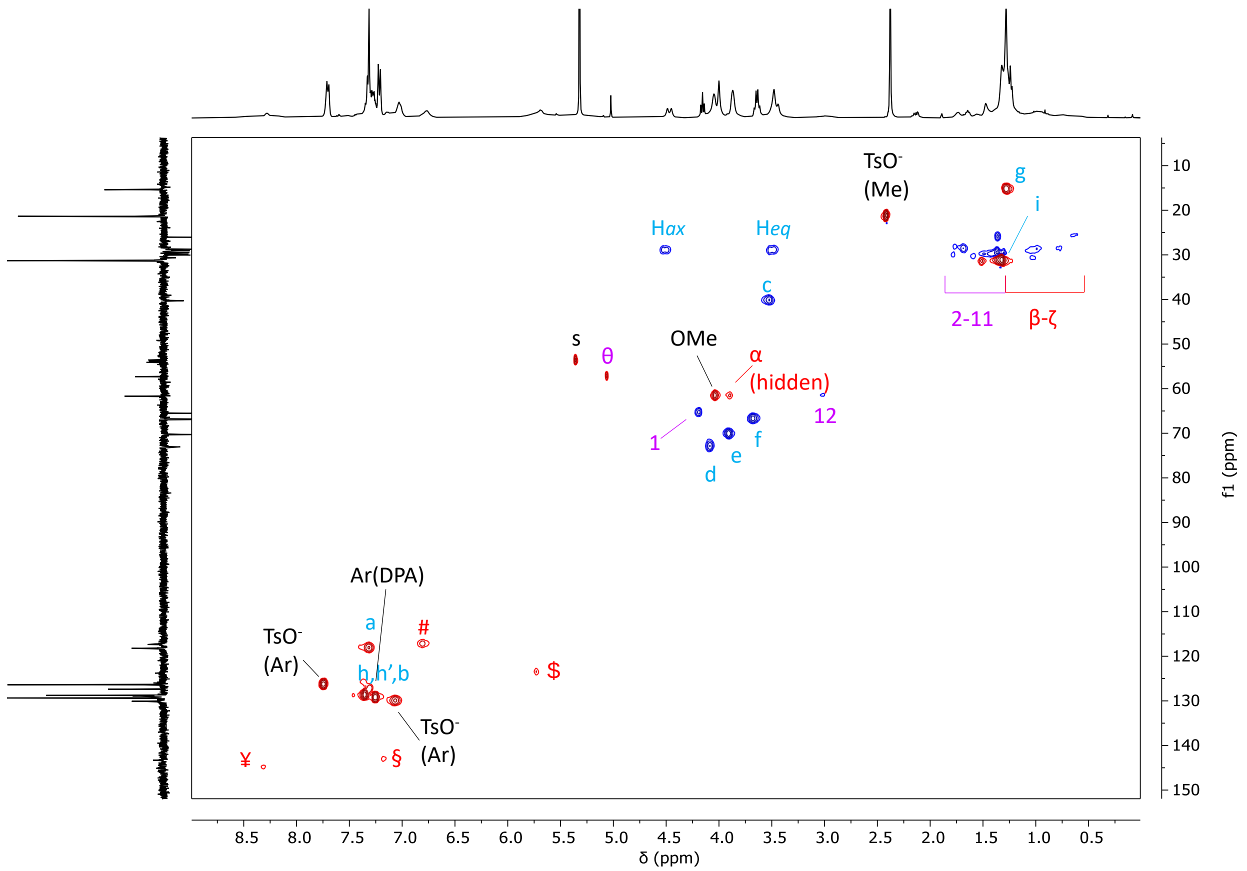

Figure S72: Edited HSQC NMR spectrum (400 MHz, CD_2Cl_2) of [3]rotaxane *R*[(**TPU-AC**)$_2$⊃**8**$_{12}$]. Positive peaks (CH_3 and CH) are shown in red, while negative ones (CH_2) are in blue.

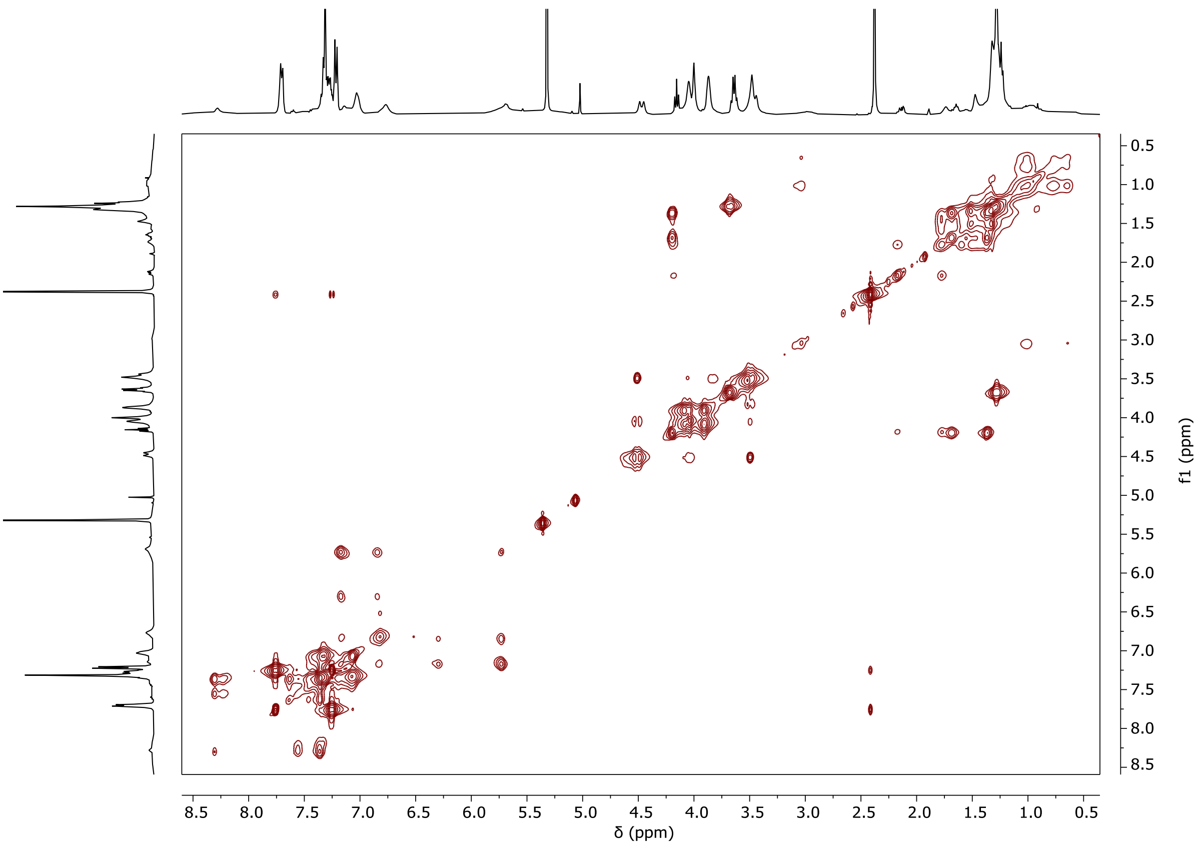

Figure S73:2D TOCSY NMR spectrum (400 MHz, CD_2Cl_2, MT = 0.06 s) of [3]rotaxane *R*[(**TPU-AC**)$_2$⊃**7**$_{12}$].

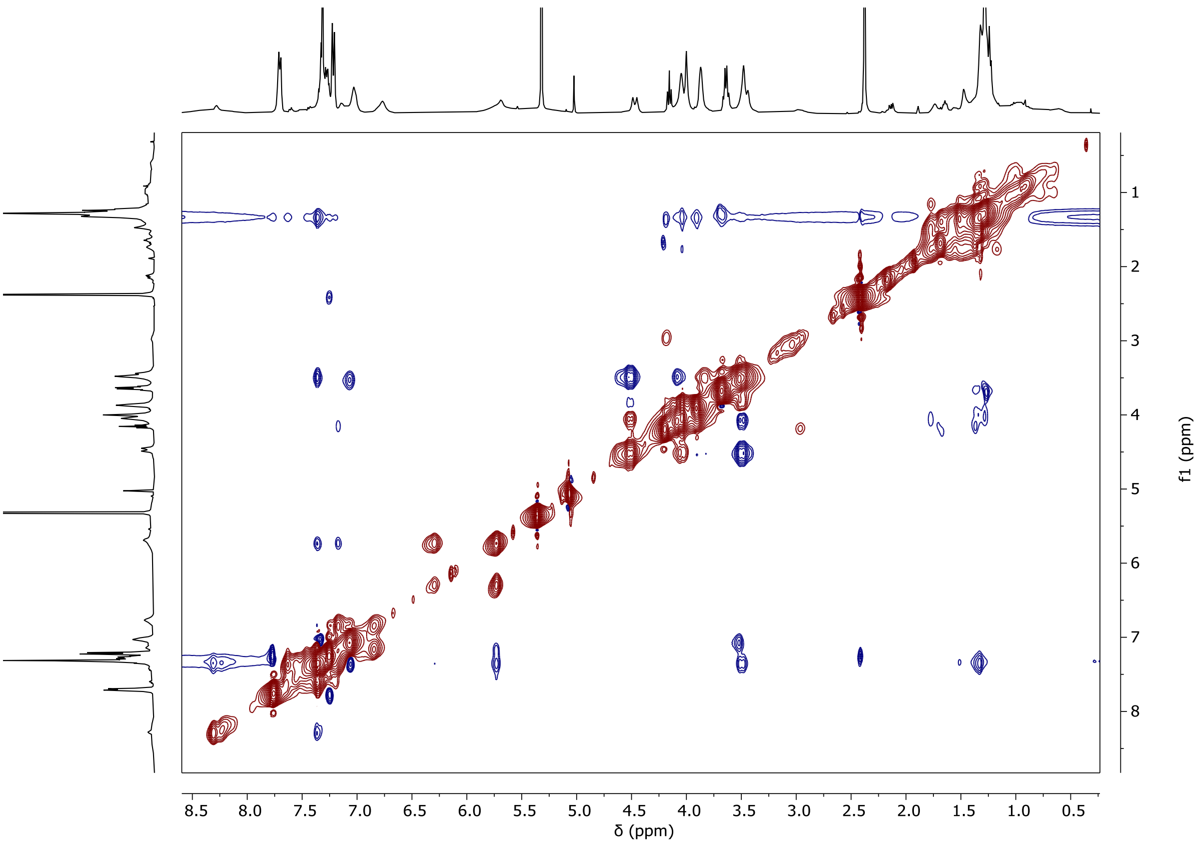

Figure S74: 2D ROESY NMR spectrum (400 MHz, CD_2Cl_2, SL = 200 ms) of [3]rotaxane *R*[(**TPU-AC**)$_2$⊃**7**$_{12}$].

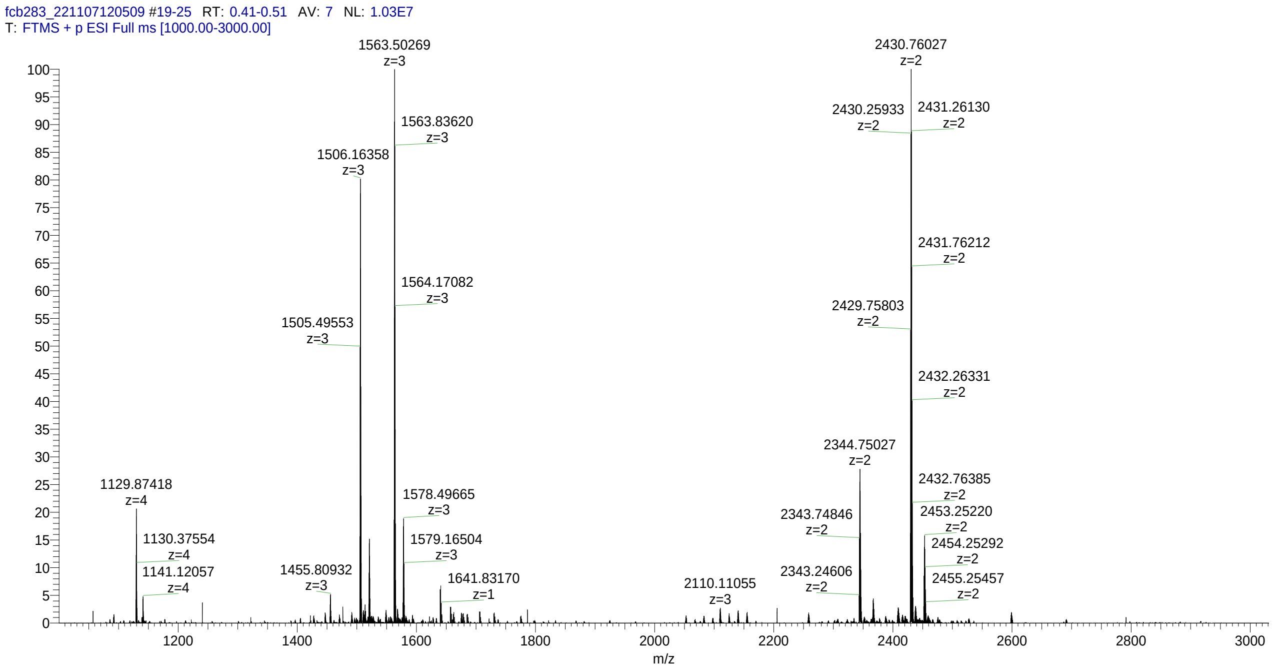

Figure S75: HR-MS (ESI, Orbitrap LQ) spectrum of [3]rotaxane *R*[(**TPU-AC**)$_2$⊃**8**$_{12}$] showing the quadruple, triply and doubly charged molecular.

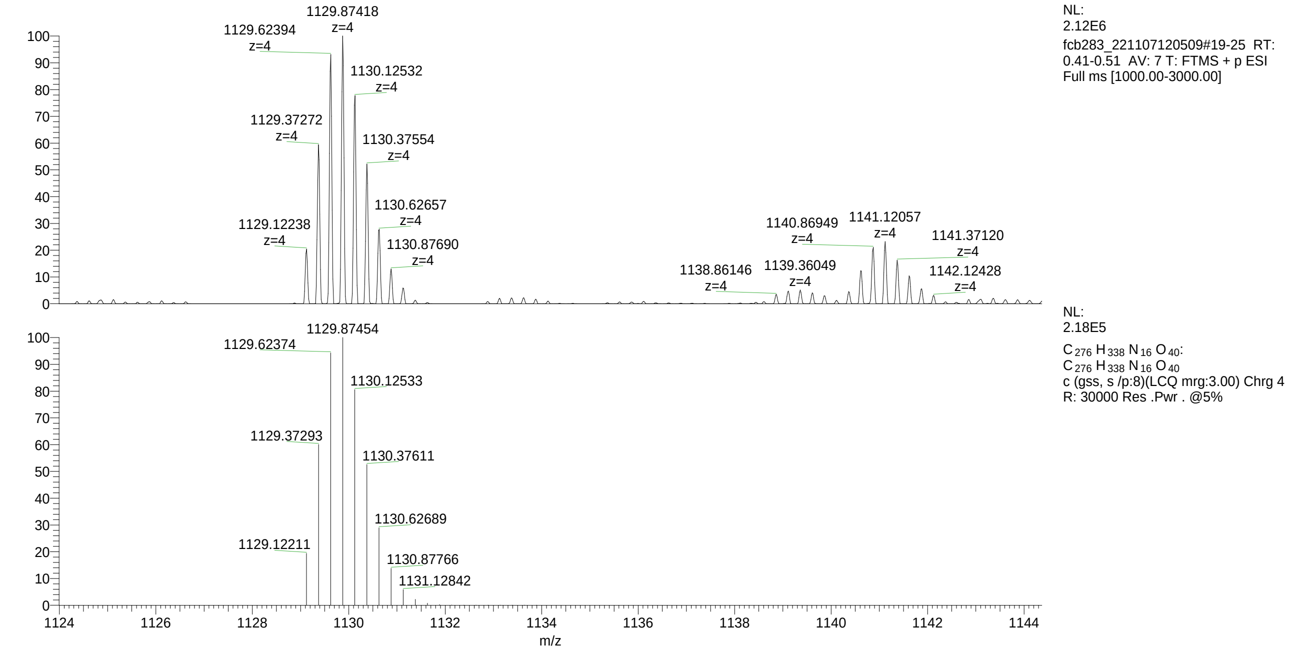

Figure S76: Inset of HR-MS (ESI, Orbitrap LQ) spectrum of compound *R*[(**TPU-AC**)$_2$⊃**8**$_{12}$]: calculated (top) and experimental (down) isotopic distribution for the quadruple charged molecular ion.

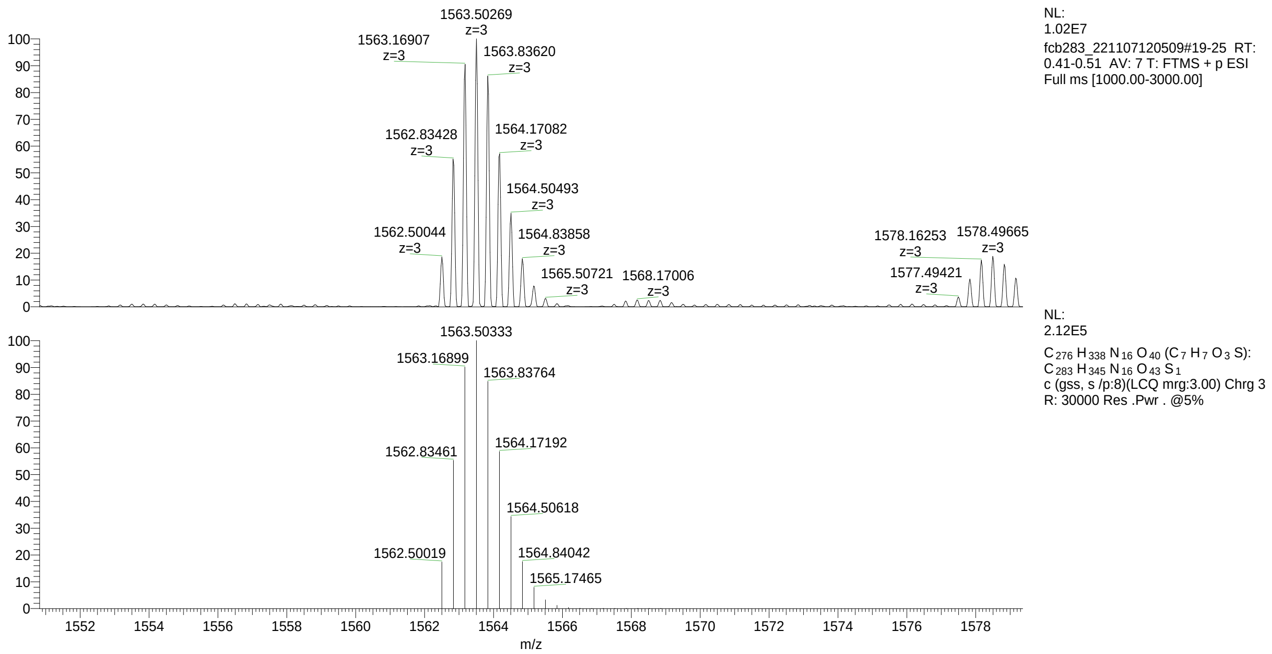

Figure S77: Inset of HR-MS (ESI, Orbitrap LQ) spectrum of compound R[(**TPU-AC**)$_2$⊃**8**$_{12}$]: calculated (top) and experimental (down) isotopic distribution for the triply charged molecular ion.

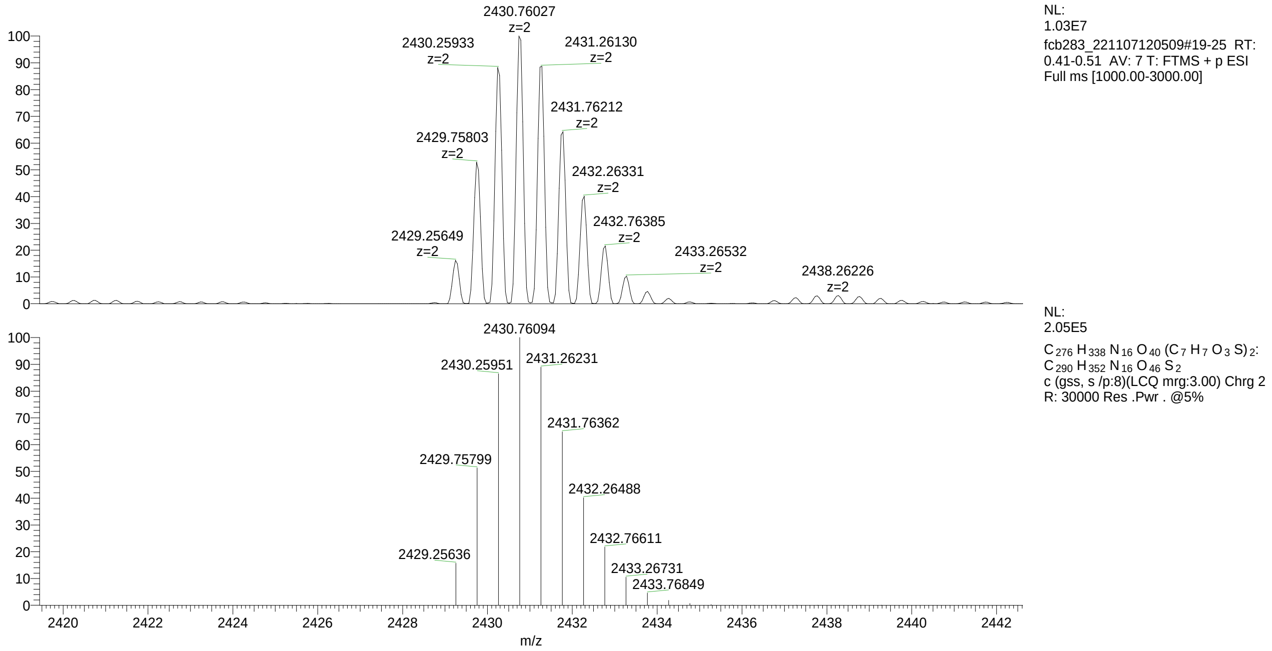

Figure S78: Inset of HR-MS (ESI, Orbitrap LQ) spectrum of compound R[(**TPU-AC**)$_2$⊃**8**$_{12}$]: calculated (top) and experimental (down) isotopic distribution for the doubly charged molecular ion.
